# Parallel pathways for serotonin biosynthesis and metabolism in *C. elegans*

**DOI:** 10.1038/s41589-022-01148-7

**Published:** 2022-10-10

**Authors:** Jingfang Yu, Merly C. Vogt, Bennett W. Fox, Chester J. J. Wrobel, Diana Fajardo Palomino, Brian J. Curtis, Bingsen Zhang, Henry H. Le, Arnaud Tauffenberger, Oliver Hobert, Frank C. Schroeder

**Affiliations:** 1 Boyce Thompson Institute and Department of Chemistry and Chemical Biology, Cornell University, Ithaca, NY 14853, USA.; 2 Department of Biological Sciences, Columbia University, Howard Hughes Medical Institute, NY 10027, USA.

## Abstract

The neurotransmitter serotonin plays a central role in animal behavior and physiology, and many of its functions are regulated via evolutionarily conserved biosynthesis and degradation pathways. Here we show that in *C. elegans*, serotonin is abundantly produced in non-neuronal tissues via phenylalanine hydroxylase (PAH-1), in addition to canonical biosynthesis via tryptophan hydroxylase (TPH-1) in neurons. Combining CRISPR-Cas9 genome editing, comparative metabolomics, and synthesis, we demonstrate that most serotonin in *C. elegans* is incorporated into *N*-acetylserotonin-derived glucosides, which are retained in the worm body and further modified via the carboxylesterase CEST-4. Expression patterns of CEST-4 suggest that serotonin or serotonin derivatives are transported between different tissues. Lastly, we show that bacterial indole production interacts with serotonin metabolism via CEST-4. Our results reveal a parallel pathway for serotonin biosynthesis in non-neuronal cell types and further suggest that serotonin-derived metabolites may serve distinct signaling functions and contribute to previously described serotonin dependent-phenotypes.

## Graphical Abstract

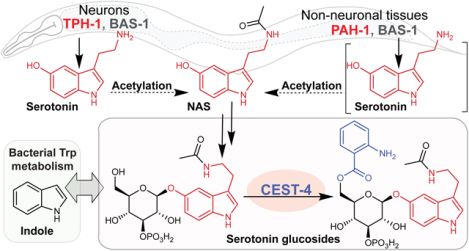

## Introduction

**Fig. 1:**
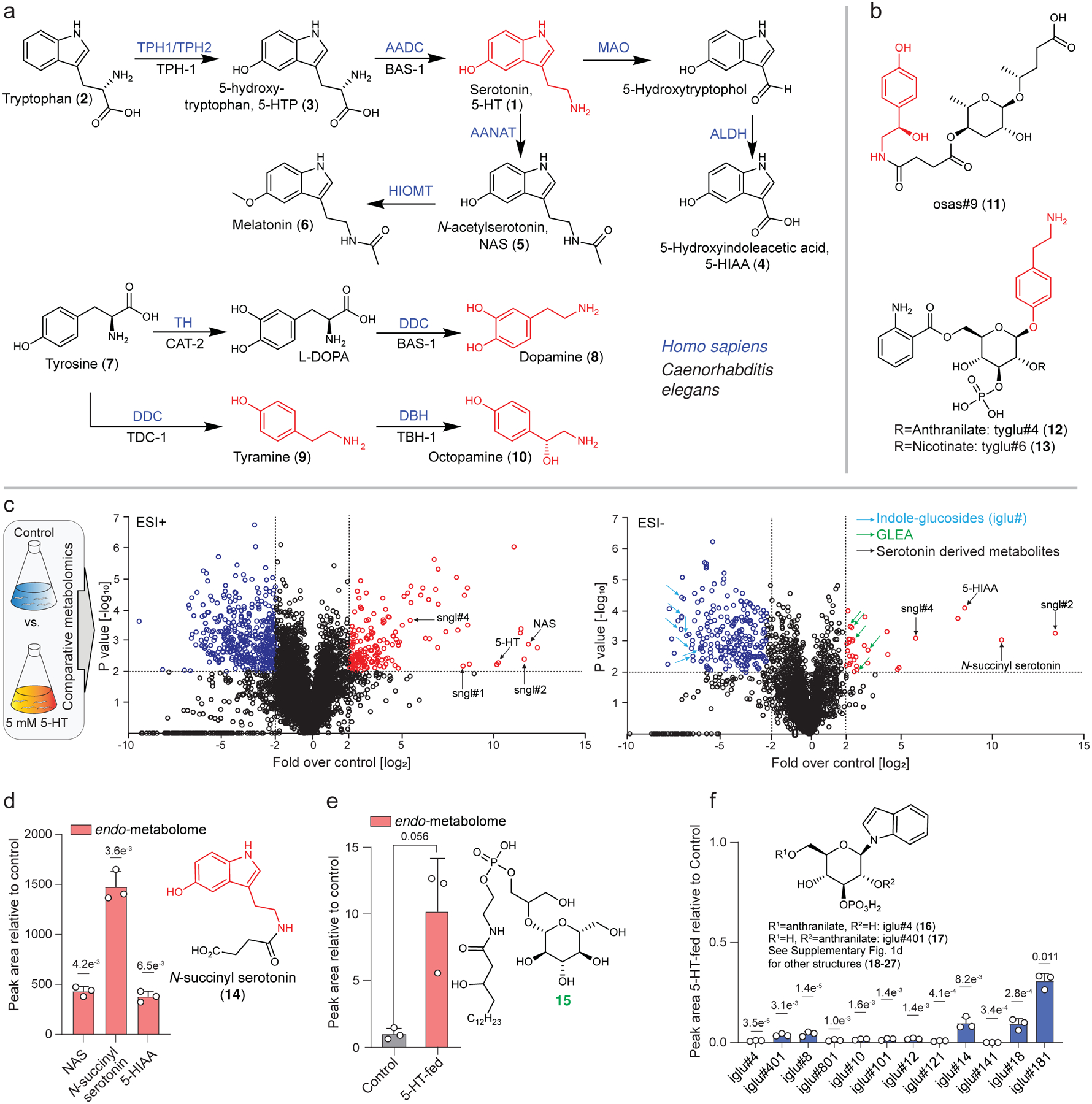
Monoamine neurotransmitter biosynthesis and serotonin supplementation. **a**, Biosynthetic pathways of neurotransmitters in human (blue) and *C. elegans* (black). **b**, Modular metabolites incorporating neurotransmitters previously identified in *C. elegans*. **c**, HPLC-MS-based comparison of the *endo*-metabolomes of untreated controls and animals treated with a high concentration of serotonin (5 mM) reveals pervasive changes. Metabolites belonging to three differential structural families are highlighted, including metabolites containing serotonin, named sngl#1–4. **d**, Abundance of known serotonin-derived metabolites in *endo*-metabolomes (worm bodies) of 5 mM serotonin-treated animals. **e**, **f**, Abundances of endocannabinoid-related phospholipids such as *N*-acyl-glycoglycerophosphoethanolamines (GLEAs, e.g., **15**) (**e**) and the indole-derived iglu-family of MOGLs (**f**) are significantly perturbed in *endo*-metabolomes of 5 mM serotonin-treated animals. Data in **d**-**f** represent 3 biologically independent experiments, and bars indicate mean ± s.d., *p*-values calculated by unpaired, two-tailed *t-*test with Welch correction.

Biosynthesis and metabolism of the neurotransmitter serotonin (5-hydroxytryptamine, 5-HT, **1**) plays a central role in vertebrate and invertebrate biology, regulating food intake^1,2^, sleep^3^, anxiety^4^, and many other physiological processes^5^. In vertebrates, serotonin is primarily produced by neuroendocrine cells in the digestive tract (e.g. the enterochromaffin cells) and serotonergic neurons of the central nervous system^6^. Its highly conserved biosynthetic pathway involves two steps; L-tryptophan (**2**) is hydroxylated by L-tryptophan-5-hydroxylase (TPH1 and TPH2 in humans)^7^, followed by decarboxylation of the resulting 5-hydroxytryptophan (5-HTP, **3**) by aromatic L-amino acid decarboxylase (AADC, Fig. 1a). Serotonin and its metabolites mediate autocrine and paracrine as well as long-range signaling functions^5^. For example, serotonin produced by the enterochromaffin cells in the gut enters the general circulation where it regulates vascular tone and blood clotting^8^. In the brain, serotonin exchange between different cell types appears to mediate its mood- and anxiety-modulating effects^9^.

In mammals, serotonin is metabolized primarily to 5-hydroxyindoleacetic acid (5-HIAA, **4**) in the liver, and, to a lesser degree, acetylated by arylalkylamine *N*-acetyltransferase (AANAT) to form *N*-acetylserotonin (NAS, **5**) in the pineal gland and retina^10^ (Fig. 1a). NAS is subsequently converted into melatonin (**6**) by hydroxyindole-*O*-methyltransferase (HIOMT). Like serotonin, NAS and melatonin have important signaling functions. In vertebrates, melatonin regulates circadian rhythm^10^, whereas NAS promotes neuronal progenitor cell (NPC) proliferation in mice^11^.

The nematode *Caenorhabditis elegans* has been used extensively as an experimentally tractable system to elucidate conserved mechanisms of serotonin signaling^2,12^. In *C. elegans*, serotonin controls locomotion, pharyngeal pumping, egg laying and many other aspects of food-related behaviors and physiology^13^. Serotonin biosynthesis in *C. elegans* has been shown to proceed via hydroxylation of tryptophan by TPH-1^14^, a close homolog of mammalian TPH1/2, and subsequent decarboxylation by aromatic amino acid decarboxylase (BAS-1, Fig. 1a)^15^. TPH-1 is believed to be required for tryptophan hydroxylation, therefore *tph-1* loss-of-function mutants have been employed extensively as a serotonin-deficient control^2,12^. However, *tph-1* mutant worms appear to retain serotonin immunoreactivity^16^, and in some studies *tph-1* mutants failed to show significant differences in pathogen avoidance compared to the wildtype (WT) animals, even though serotonin contributes to regulating the associated behaviors^17,18^. Moreover, the selective serotonin reuptake inhibitor (SSRI) fluoxetine and the tricyclic antidepressant imipramine, still induce egg-laying responses in *tph-1* mutants, suggesting residual serotonin-dependent signaling^19^.

Recent analyses of the metabolomes of *C. elegans* and other nematode species have demonstrated that two of the three tyrosine (**7**)-derived monoamine neurotransmitters (**8**–**10**), tyramine (**9**) and octopamine (**10**), partake in previously unrecognized metabolic pathways^20,21^. For example, in osas#9 (**11**), a dispersal signal produced by starved *C. elegans* L1 larvae, octopamine is combined with succinate and an ascaroside (Fig. 1b). Similarly, tyramine is integrated into a large library of modular glucosides (MOGLs) that combine building blocks from all major primary metabolic pathways, e.g. tyglu#4 (**12**) and tyglu#6 (**13**)^22,23^.

We set out to investigate the origin and metabolic fate of serotonin in *C. elegans* using both chemical and genetic approaches. We first conducted a series of supplementation experiments with serotonin and *N*-acetylserotonin to uncover serotonin-derived metabolites and associated metabolic pathways. We then performed an unbiased comparative metabolomics investigation of *C. elegans* genetic mutants required for serotonin biosynthesis. Our analyses revealed a non-canonical pathway of serotonin biosynthesis via phenylalanine hydroxylase (PAH-1) and led to the identification of a family of serotonin-incorporating modular glucosides as the most abundant serotonin-derived metabolites in *C. elegans*.

## Results

### Modular glucosides are major serotonin metabolites.

To investigate serotonin metabolism in *C. elegans*, we began by supplementing WT animals with a supraphysiological concentration of serotonin (5 mM), which is commonly used to study serotonin-dependent behaviors^2,12,14^. The *endo*-metabolomes (worm bodies) and *exo*-metabolomes (conditioned media) were separately extracted and analyzed by high-performance liquid chromatography coupled to high-resolution mass spectrometry (HPLC-HRMS, Fig. 1c). These analyses revealed dramatic changes in the abundances of several hundred different metabolites in response to serotonin treatment (Fig. 1c). As most abundant serotonin-derived metabolites we identified NAS, *N*-succinyl serotonin (**14**), and 5-HIAA, in addition to smaller amounts of melatonin (Fig. 1d and Supplementary Fig. 1a-b). However, the vast majority of the metabolites affected by 5 mM serotonin treatment were not serotonin-derived; for example, we observed a stark increase in the abundance of a series of endocannabinoid derivatives (e.g., **15**), the recently described *N*-acyl-glycoglycerophosphoethanolamines (GLEAs)^24^, whereas production of diverse indole-derived MOGLs^22^ (**16–27**) was strongly suppressed (Fig. 1c,e,f and Supplementary Fig. 1c,d).

**Fig. 2:**
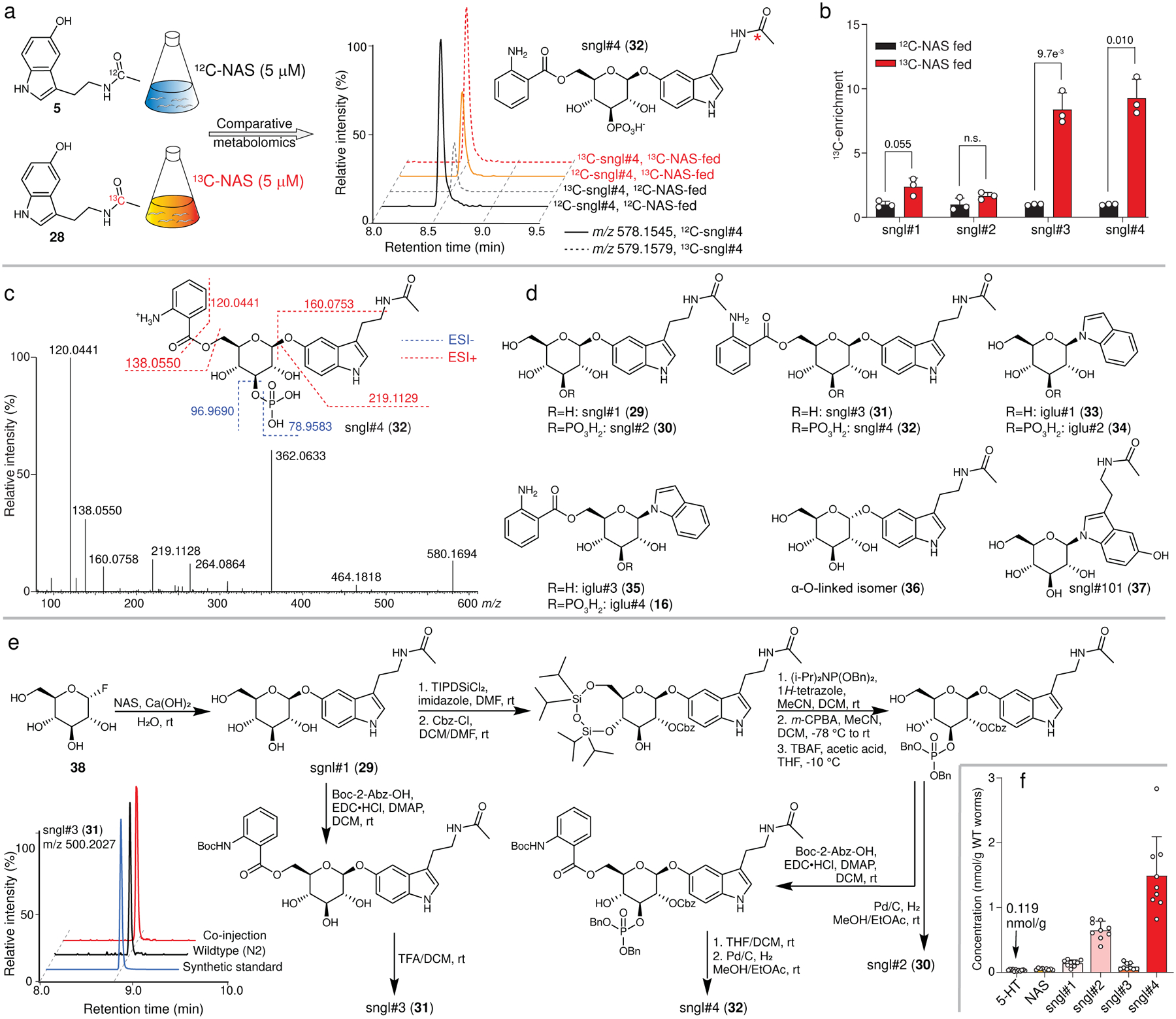
Identification of serotonin derived MOGLs. **a**, Schematic for comparative metabolomics using a low concentration of ^13^C-labeled NAS (5 μM). HPLC traces demonstrate ^13^C-labeling of novel serotonin metabolite, sngl#4. **b**, ^13^C-enrichment of the four most abundant NAS-derived metabolites. **c**, ESI^+^ MS/MS spectrum and proposed fragmentation of sngl#4. **d**, Structures of identified serotonin-derived MOGLs and related MOGLs incorporating indole instead of serotonin. **e**, Synthetic scheme for sngl#1–4 (see Supplementary Information for details) and ESI^+^ ion chromatograms for sngl#3 in *C. elegans*, synthetic sngl#3, and for co-injection of natural and synthetic sample. **f**, Concentrations of free serotonin (5-HT), NAS, sngl#1, sngl#2, sngl#3, and sngl#4 in WT *C. elegans*. Data in **b** (n = 3) and **f** (n = 9, except for 5-HT measurement, where n = 12) represent biologically independent experiments, and bars indicate mean ± s.d., *p-*values calculated by unpaired, two-tailed *t*-test with Welch correction; n.s., not significant.

These dramatic changes in phospholipid and indole metabolism as well as the production of large quantities of *N*-succinyl serotonin, of which only trace amounts were detected in control animals, indicated that treatment with unphysiologically high concentrations of serotonin (e.g., 5 mM, as commonly used^2,12,14^) may confound outcomes. To mitigate such metabolic perturbations, we employed a stable isotope-labeling approach that would enable tracking downstream metabolites even when supplementing with low (micromolar) concentrations (Fig. 2a). For this purpose, we selected NAS, given that serotonin supplementation revealed NAS as the primary serotonin metabolite (Supplementary Fig. 1b), consistent with previous studies^25^. Furthermore, a stable isotope label could be easily integrated into NAS (Supplementary Fig. 1).

Comparative metabolomic analysis of WT animals treated with stable isotope-labelled *N-*acetylserotonin (^13^C-NAS, **28**) or unlabeled NAS (^12^C-NAS) revealed four major downstream metabolites (Fig. 2b). Their MS/MS fragmentation patterns suggested that they represent a series of modular glucosides (MOGLs)^23,26^, which we named sngl#1–4 (**29–32**, *s*erotoni*n gl*ucoside, Fig. 2c,d). The MS/MS fragmentation patterns further indicated that some of these putative serotonin-derived MOGLs incorporate phosphate and anthranilic acid moieties, similar to previously described MOGLs incorporating indole, iglu#1–4 (**33**–**35**, **16**) (Fig. 2d). Because the MS data did not allow distinguishing between the large number of isomeric candidate structures that could be derived from these building blocks, we isolated a sample of sngl#2 (**30**) from WT animals treated with a high concentration of serotonin for further characterization via NMR spectroscopy. Analysis of the NMR spectra of isolated sngl#2 (Supplementary Fig. 2) confirmed the presence of a β-glucoside and indicated 3-*O*-phosphorylation, consistent with the structures of other phosphorylated MOGLs, e.g., iglu#2 and iglu#4^27^. In addition, the NMR spectra of sngl#2 suggested that the NAS moiety is attached via the phenolic hydroxy group instead of the indole nitrogen, in contrast to previously characterized MOGLs such as iglu#2.

To confirm these assignments, we first synthesized the α-*O*-, β-*N*-, and β-*O*-linked isomers of *N*-acetylserotonin glucoside, **36**, **37**, and **29**, respectively (Fig. 2e and Supplementary Fig. 3). Comparison of HPLC retention times on two different columns and MS/MS spectra indicated that sngl#1 corresponds to the β-*O*-linked isomer (**29**) (Supplementary Fig. 2a,b). In addition, careful inspection of the HPLC-HRMS data revealed a small amount of the β-*N*-linked isomer (sngl#101, **37**) in both serotonin-fed and WT worms (Supplementary Fig. 2c-f). To establish the exact structures of the remaining three serotonin-derived MOGLs, sngl#2–4, we synthesized authentic samples using a recently developed strategy^28^ that employs α-D-fluoroglucose (**38**) for selective preparation of the *O*-linked NAS-glucoside (sngl#1), followed by a protection/deprotection sequence to install the phosphate at position 3 and then attach the anthranilic acid moiety at position 6 of the glucose (Fig. 2e). Comparison of HPLC retention times and MS/MS spectra of synthetic samples of sngl#2–4 confirmed their identity with the naturally occurring serotonin metabolites (Fig. 2e and Supplementary Fig. 2g-k). Identification of sngl#4 further allowed to propose the structure of a less abundant derivative, sngl#6 (**39**, Supplementary Fig. 2l). Next, we used authentic standards to quantify serotonin, NAS, and sngl#1–4 levels in WT animals, which revealed that NAS-derived glucosides represent by far most abundant metabolites incorporating serotonin as a building block (Fig. 2f and Supplementary Fig. 4a–g). Notably, sngl#1–4 are retained in the worm body to a much greater extent than the other two abundant serotonin metabolites we detected, NAS and 5-HIAA, which are primarily excreted (Supplementary Fig. 2m), suggesting that serotonin-derived MOGLs may serve specific intra-organismal functions.

### Phenylalanine hydroxylase contributes to serotonin pools.

**Fig. 3:**
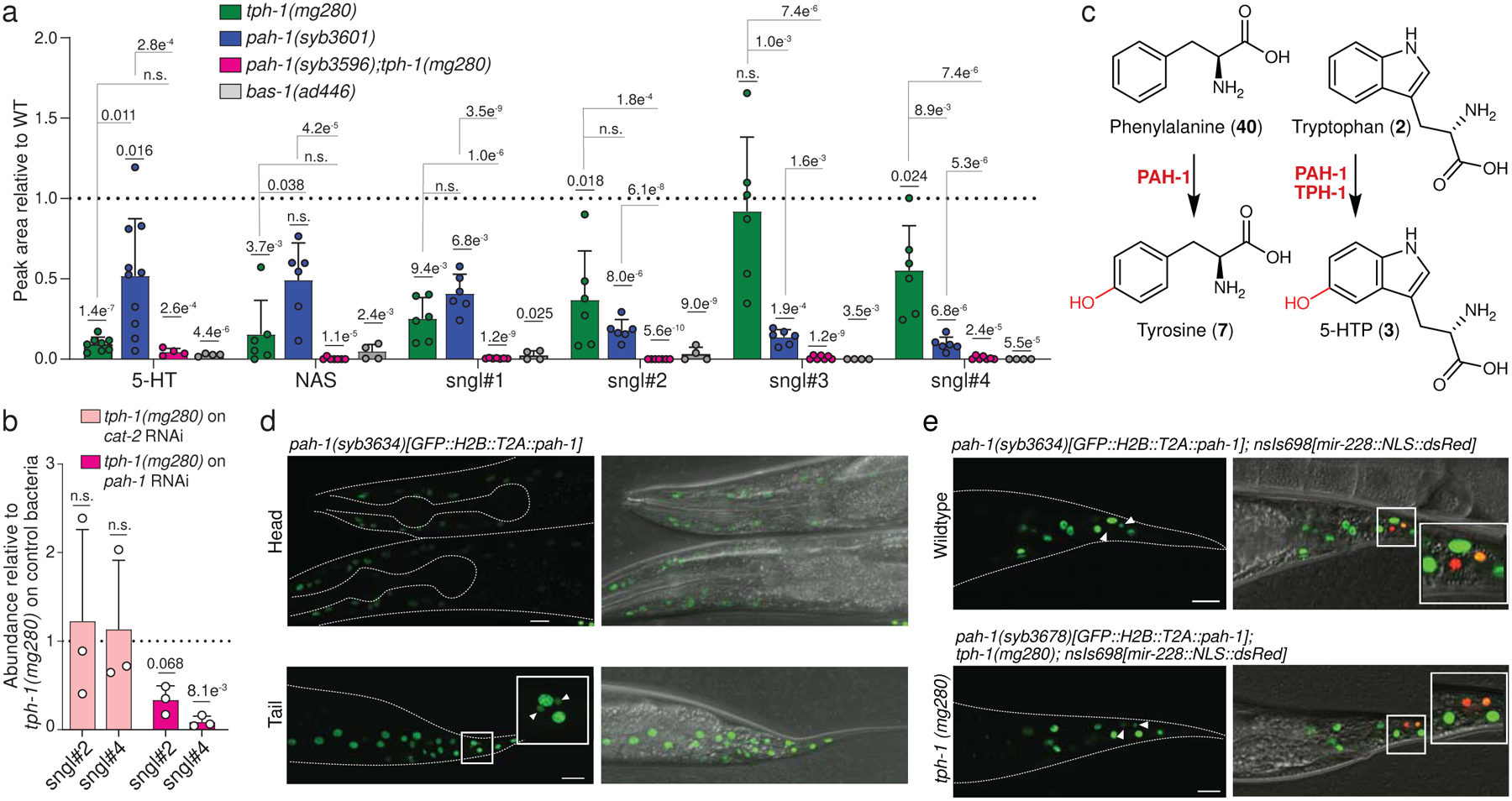
Phenylalanine hydroxylase PAH-1 contributes to serotonin biosynthesis. **a**, Abundance of serotonin metabolites in the *endo*-metabolomes of *tph-1(mg280)*, *pah-1(syb3601)*, and *pah-1(syb3596);tph-1(mg280)* mutant animals relative to WT. **b**, Abundances of sngl#2 and sngl#4 in *endo*-metabolomes of *tph-1(mg280)* mutants fed *pah-1* or *cat-2* dsRNAi bacteria, relative to RNAi control. Data in **a** were collected as biologically independent replicates as follows: *tph-1(mg280)* (n = 8 for 5-HT measurement, n = 6 for NAS and sngl#1–4 measurement), *pah-1(syb3601)* (n = 10 for 5-HT measurement, n = 6 for NAS and sngl#1–4 measurement), *pah-1(syb3596);tph-1(mg280)* (n = 4 for 5-HT measurement, n = 7 for NAS and sngl#1–4 measurement)*, bas-1(ad446)* (n = 4). Data in **b** (n =3) represents biologically independent experiments. Bars in **a** and **b** indicate mean ± s.d., *p*-values calculated by unpaired, two-tailed *t-*test with Welch correction, comparing mutant and WT samples, and between mutant animals; n.s., not significant. **c**, Inferred biosynthetic roles of PAH-1 and TPH-1 in *C. elegans*. **d**, **e**, GFP::H2B::T2A::PAH-1 was strongly expressed in epidermal cells and in glial cells in the tail in both WT (**d**) and *tph-1(mg280)* (**e**) mutants. Panglial microRNA *mir-228*-fused to DsRed was crossed into both WT and *tph-1(mg280)* mutants, revealing *pah-1* expression in a pair of glial cells (indicated by white triangles). Scale bar for all images = 15 μm. For each genotype in **d** and **e**, at least 10 animals were scored at Day 1 of adulthood for GFP expression under well fed conditions on at least 2 different days.

We next measured abundances of free serotonin, NAS, and sngl#1–4 in null mutants of genes of the established serotonin biosynthetic pathway (Fig 1a). As expected, production of all serotonin derivatives was largely abolished in *bas-1(ad446)* null mutants, consistent with BAS-1 serving as the sole amino acid decarboxylase required for converting 5-HTP into serotonin (Fig. 3a and Supplementary Fig. 3a). However, production of serotonin, NAS, and sngl#1–4 was not abolished in null mutants of the canonical tryptophan hydroxylase *tph-1* (Fig. 3a and Supplementary Fig. 3b,c). This was unexpected, because TPH-1 is generally presumed to be required for serotonin biosynthesis in *C. elegans*^14^. Abundances of unmodified serotonin, NAS, and sngl#1–2 were reduced by roughly 50–80% in two independent loss-of-function alleles, *tph-1(mg280)* and *tph-1(n4622)*, relative to WT animals (Fig. 3a and Supplementary Fig. 3b,c). In contrast, abundances of sngl#3 and sngl#4 were WT-like in *tph-1(n4622)* mutants, while *tph-1(mg280)* mutants showed a ~50% reduction of sngl#4. These results indicated that, whereas BAS-1 is required for the biosynthesis of serotonin, TPH-1 is not the only enzyme catalyzing tryptophan hydroxylation, the rate-limiting step in serotonin biosynthesis in *C. elegans*^14^.

As candidates for other enzymes participating in serotonin biosynthesis, we considered PAH-1 (phenylalanine hydroxylase) and CAT-2 (tyrosine hydroxylase), which are the other two iron- and tetrahydrobiopterin (BH4)-dependent aromatic amino acid hydroxylases (AAAHs) in *C. elegans*^13^. Notably, previous studies demonstrated that recombinant PAH-1 can hydroxylate both phenylalanine (**40**) and tryptophan substrates *in vitro*^29,30^, and *pah-1* homologs in *Drosophila* and mouse have been shown to contribute to serotonin biosynthesis^30,31^. In addition, CAT-2 has been reported to exhibit tryptophan hydroxylase activity^32^. To test whether PAH-1 and CAT-2 participate in tryptophan hydroxylation, we measured production of serotonin metabolites in *tph-1(mg280)* null mutants treated with *cat-2* or *pah-1* RNAi (Fig. 3b). We monitored sngl#2 and sngl#4, because these phosphorylated serotonin metabolites ionize well and thus offered the greatest dynamic range for detecting changes in serotonin metabolism via HPLC-HRMS at the smaller scale of RNAi experiments. We found that abundances of sngl#2 and sngl#4 were almost completely abolished by *pah-1* RNAi in *tph-1(mg280)* animals, whereas *cat-2* RNAi had no effect (Fig. 3b). In addition, we found that abundances of NAS and sngl#1–4 were significantly reduced in *pah-1(syb3596)* null mutants (Fig. 3a), to a similar extent as in *tph-1(mg280)* null mutants, but were largely unchanged in *cat-2(e1112)* null mutants (Extended Data Fig. 3d). These results suggested that PAH-1 contributes to 5-hydroxylation of tryptophan, in parallel with TPH-1 (Fig. 3c).

To further corroborate this hypothesis, we generated a *pah-1(syb3596);tph-1(mg280)* double null mutant using CRISPR-Cas9 genome engineering (Supplementary Fig. 5). Production of NAS and sngl#1–4 was completely abolished in the *pah-1;tph-1* double null mutant, indicating that both *pah-1* and *tph-1* contribute to the production of the major serotonin-derived metabolites in *C. elegans* (Fig. 3a, Supplementary Fig. 3a). We also tested to what extent PAH-1 contributes to levels of free serotonin and other monoamine neurotransmitters. The susceptibility to oxidation and *N*-acetylation of free serotonin complicates its accurate detection and quantification in biological samples, especially in whole organism extracts. To measure serotonin levels, we therefore employed a protocol that converts free serotonin into the more stable dansyl derivatives **41** and **42** that can be detected with high sensitivity by HPLC-HRMS^33^ (Supplementary Fig. 3e and Supplementary Fig. 6). We found that free serotonin was strongly reduced, though not fully abolished, in *tph-1(mg280)* mutant worms, whereas free serotonin levels in *pah-1(syb3601)* mutant worms were reduced to a lesser extent (Fig. 3a). Serotonin levels in *pah-1(syb3596);tph-1(mg280)* double mutant worms were on average lower than in *tph-1(mg280)* single mutants, though the difference did not reach statistical significance. In addition, abundances of the tyrosine-derived neurotransmitters, dopamine and tyramine^13^, were decreased in *pah-1(syb3601)* single and *pah-1(syb3596);tph-1(mg280)* double mutant worms, consistent with reduced tyrosine levels resulting from lack of phenylalanine hydroxylation in *pah-1* knockout mutants (Supplementary Fig. 3f,g)^29^. Collectively, these results indicate that TPH-1 is the primary source of free serotonin, whereas TPH-1 and PAH-1 both contribute to the biosynthesis of the downstream serotonin metabolites NAS, sngl#1, and sngl#2. In turn biosynthesis of sngl#3 and sngl#4 is mostly PAH-1-dependent (Fig. 2f, 3a).

Previous immunofluorescence studies detected free serotonin in a subset of *tph-1* expressing neurons in *C. elegans*, including NSM, ADF and HSN^14^. *pah-1* expression has been detected in the epidermis, seam cell, and tail, using immunofluorescence and fluorescent protein reporters^34^. To independently validate and further investigate *pah-1* expression, we used CRISPR-Cas9 genome engineering to insert *gfp:h2b* at the *N*-terminus of the *pah-1* locus, separated by a T2A self-cleaving peptide (GFP::H2B::T2A::PAH-1). This strategy results in separation of GFP::H2B from PAH-1 during translation, and subsequent nuclear translocation of the fluorescent protein, but not PAH-1, allowing for easier identification of *pah-1*-expressing cells without interfering with protein function. We detected GFP fluorescence in epidermal cells, as previously reported, but also in two socket glia cells in the tail (Fig. 3d). Socket glia cell expression was confirmed by co-localization of GFP-H2B with the pan-glial microRNA *mir-228* promoter, driving expression of a nuclear-localized red fluorescent protein (RFP) (*mir-228p*::NLS-RFP, Fig. 3e), as well as with a phasmid socket glia-specific marker (*grl-2p*::CFP, Supplementary Fig. 7a). Comparison of *pah-1* expression in WT and *tph-1(mg280)* background revealed no overt differences, indicating that loss of *tph-1* does not strongly perturb *pah-1* expression patterns, as monitored via GFP::H2B (Supplementary Fig. 7b).

**Fig. 4:**
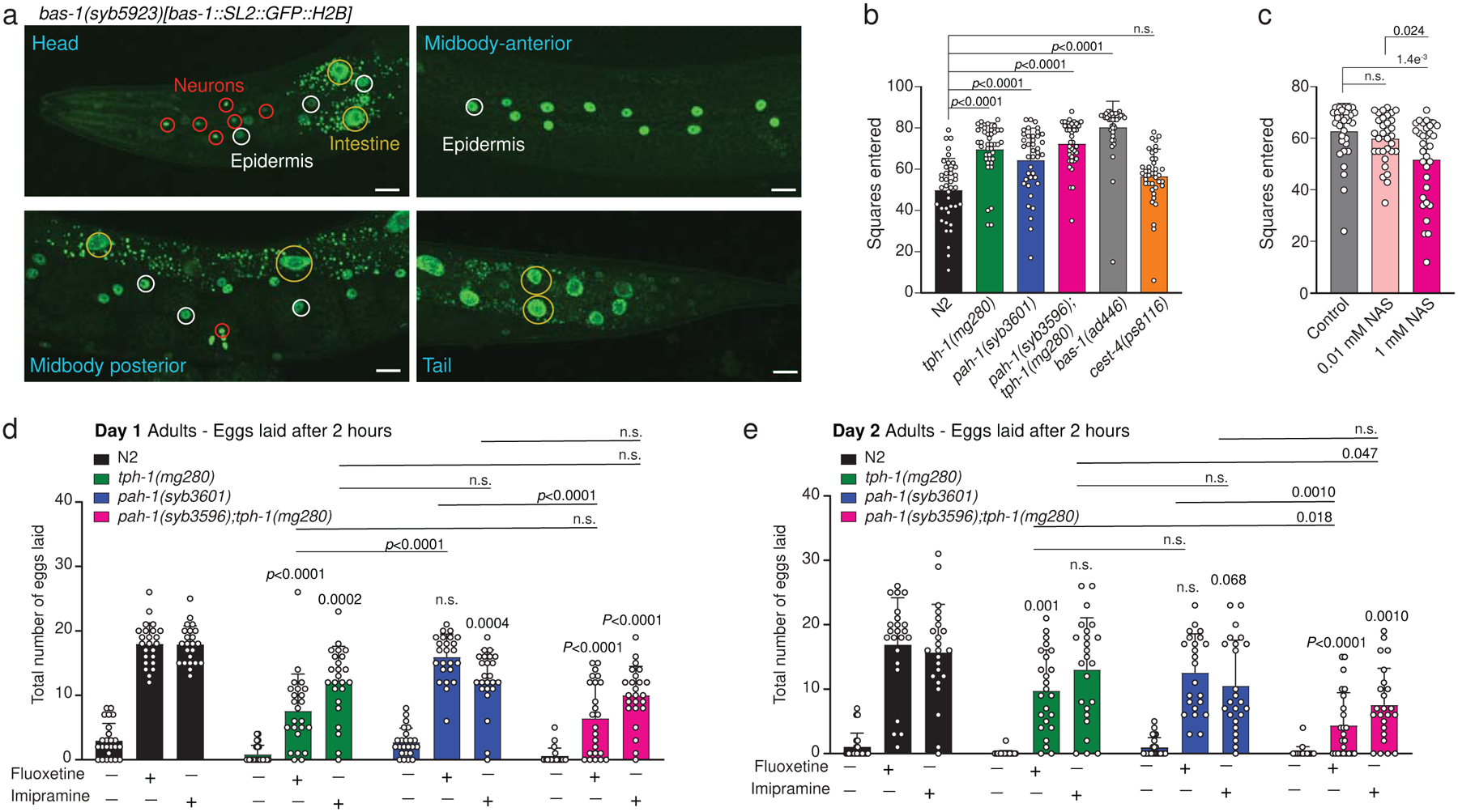
Phenylalanine hydroxylase PAH-1 contributes to serotonin-related behaviors. **a**, BAS-1::SL2::GFP::H2B is expressed in neuronal cells, as well as in the epidermis and intestine. Scale bar for all images = 15 μm. At least 10 animals were scored at Day 1 of adulthood for GFP expression under well fed conditions on at least 2 different days. **b**, Loss of PAH-1 significantly increases exploration behavior after 13.5 h to a similar extent as loss of TPH-1. **c**, 1 mM exogenous NAS partially rescued increased exploration behavior of *bas-1* mutants after 13.5 h. **d**, Total number of eggs laid in response to SSRIs fluoxetine (0.5 mg/mL) and imipramine (0.75 mg/mL) were determined within 2 h of drug exposure in 1-day old (**d**) and 2-day old (**e**) WT (N2; black), *tph-1(mg280)* (green), *pah-1(syb3601)* (blue), and *pah-1(syb3596); tph-1(mg280)* (red) hermaphrodites. Data in **b** (n = 20 per experiment per genotype and treatment) were collected in 2 biologically independent experiments. Data in **c** (n = 10 and n = 11 for the first two experiments and n = 12 for the third experiment), data in **d** (n =8) and **e** (n =8) were collected in 3 biologically independent experiments. Statistical significance (**b**-**e**) was determined using one-way ANOVA with Tukey’s post-hoc test.

A critical step in the production of serotonin is the decarboxylation of 5-HTP by BAS-1 (AADC in humans, Fig. 1a). However, to date, promoter-based fluorescent *bas-1* transgenic reporter strains revealed *bas-1* expression in mono-aminergic neurons^35^, including serotonergic, *tph-1*-expressing neurons, but not in *pah-1*-expressing epidermal or tail glia cells. Thus, to determine whether *pah-1*-dependent serotonin biosynthesis occurs in a *tph-1*-like cell-autonomous fashion, which would depend on co-expression of *bas-1*, we generated an endogenous, *C*-terminally SL2::GFP::H2B-tagged *bas-1* reporter strain using CRISPR/Cas-9. This endogenous reporter confirmed previously reported expression in all monoaminergic neurons, but further revealed robust expression of *bas-1* in the epidermis and intestine (Fig. 4a). These findings establish co-expression of *pah-1* and *bas-1* in the epidermis, supporting the hypothesis that serotonin can be synthesized via PAH-1 cell-autonomously in *C. elegans*. Alternatively, PAH-1-dependent serotonin could be produced cell-nonautonomously, e.g., via tryptophan hydroxylation in glial cells followed by decarboxylation via BAS-1 in the intestine; however, given that 5-HTP could not be detected in either WT or *bas-1* animals (Supplementary Fig. 3h,i) this scenario is perhaps less likely.

### PAH-1 contributes to serotonin-related behaviors.

To understand the role of *pah-1-*dependent serotonin production, we tested *pah-1(syb3601)* single and *pah-1(syb3596);tph-1(mg280)* double mutants for changes in previously reported serotonin-dependent behaviors. Using automated single worm tracking analysis^36^, we did not detect significant gross locomotory defects in *pah-1(syb3601)* mutant animals over a short three-minute tracking period (Extended Data Fig. 4a,b). Moreover, loss of *pah-1* in a *tph-1(mg280)* mutant background did not exacerbate changes in crawling amplitudes, foraging, body bends or backward motion of *tph-1(mg280)* mutant animals (Supplementary Fig. 4a,b). However, *pah-1(syb3601)* mutant animals displayed significantly increased exploratory behavior over 6-h and 13.5-h time periods compared to WT, comparable to the increase in exploration of *tph-1(mg280)* mutants (Fig. 4b, Supplementary Fig. 4c). Thus, our results suggest that PAH-1-dependent serotonin production contributes to regulating exploratory behavior^37^. Given that most serotonin in *C. elegans* is rapidly metabolized, we then asked whether serotonin metabolites play a role for the observed exploratory behavior phenotype. To this end, we measured the effect of NAS supplementation on the exploratory behavior of *bas-1(ad446)* mutants, which are serotonin-deficient (Fig. 3a) and correspondingly show greatly increased exploratory behavior (Fig. 4b). We selected NAS for this supplementation experiment, because our initial supplementation study with stable-isotope labeled NAS showed that this compound is easily taken up by *C. elegans* and converted into the downstream metabolites sngl#1–4. NAS supplementation partially rescued the increased exploratory behavior of *bas-1(ad446)* mutants (Fig. 4c), whereas supplementation of WT animals had no significant effect (Supplementary Fig. 4d), indicating that NAS or the NAS-derived sngl’s contribute to regulating exploratory behavior.

Exposure to exogenous serotonin or to compounds that modulate serotonin signaling, such the SSRI fluoxetine or the tricyclic antidepressant imipramine, can induce egg-laying in *C. elegans*, and this egg-laying response is attenuated in worms with reduced serotonin levels^19^. We confirmed that reduction of serotonin levels in *tph-1(mg280)* mutant animals results in a reduced, but not completely abolished egg-laying response to fluoxetine and imipramine (Fig. 4d,e). The remaining response of *tph-1(mg280)* mutant animals to SSRIs has been attributed to serotonin-independent mechanisms mediated via dopaminergic signaling^31^. However, we hypothesized that this remaining response could in part be due to TPH-1-independent serotonin production via PAH-1. Thus, we tested the effects of fluoxetine and imipramine on egg-laying behavior in *pah-1(syb3601)* and *pah-1(syb3596);tph-1(mg280)* mutant animals. While loss of PAH-1 did not affect the egg-laying response to fluoxetine, *pah-1(syb3601)* mutants displayed a significantly reduced egg-laying response to imipramine in one-day-old adult hermaphrodites (Fig. 4d). Moreover, additional loss of *pah-1* in a *tph-1(mg280)* mutant background exacerbated the egg-laying response to both fluoxetine and imipramine compared to *tph-1(mg280)* mutants in two-day-old adults (Fig. 4e). Reduced induction of egg laying in *pah-1(syb3601)* and *pah-1(syb3596);tph-1(mg280)* mutants in response to SSRI treatment is consistent with decreased serotonin signaling in *pah-1* mutant background. Taken together, our data demonstrate that PAH-1-dependent production of serotonin and/or serotonin-derived metabolites significantly contribute to two well-described functions of serotonin signaling, egg-laying and exploratory behavior, in *C. elegans*.

### Biosynthesis of serotonin-derived MOGLs.

**Fig. 5:**
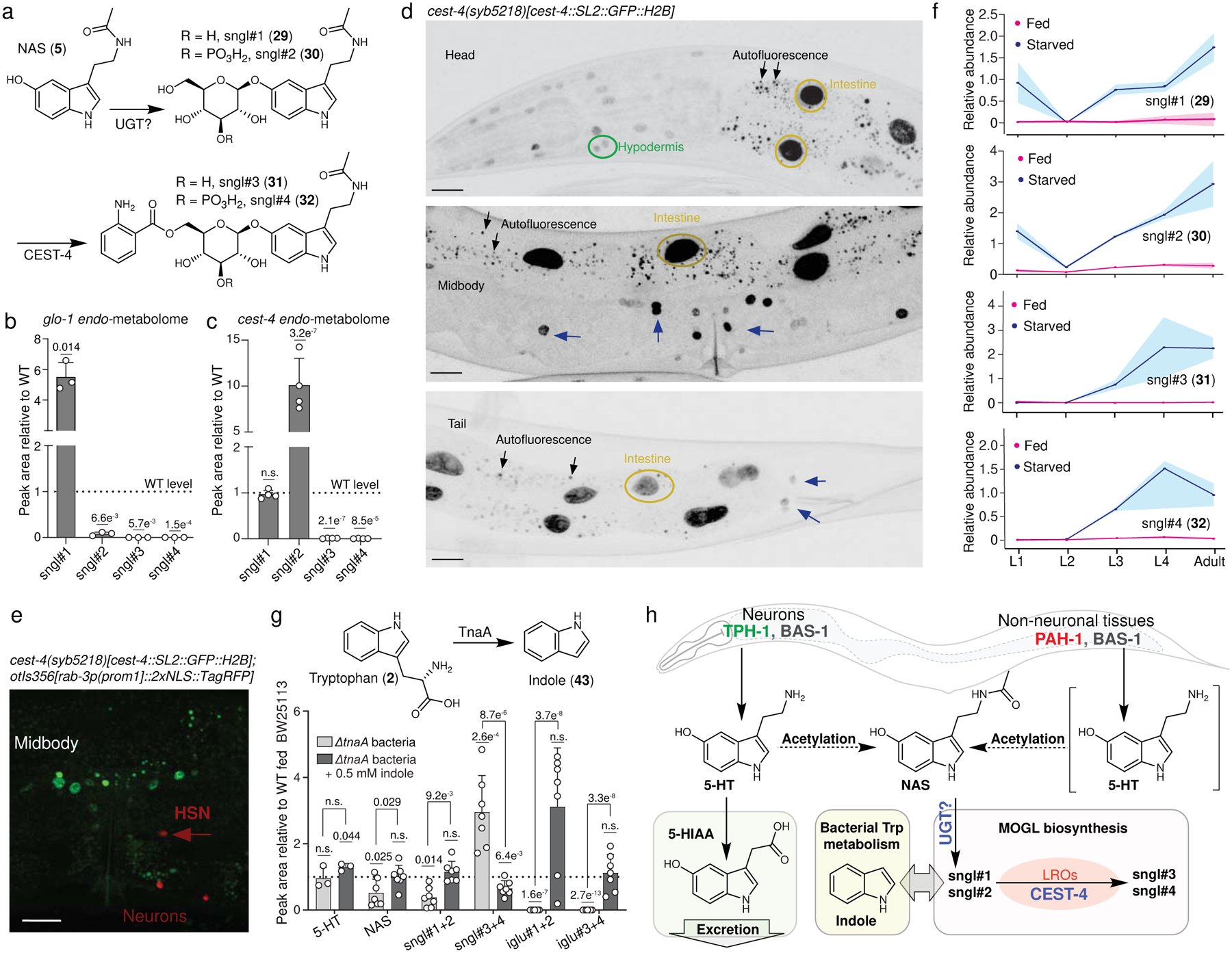
Biosynthesis model for serotonin-derived metabolites. **a**, Proposed biosynthesis of serotonin-derived MOGLs. **b**, **c**, Serotonin-derived MOGLs sngl#3 and sngl#4 are abolished in *endo*-metabolomes of *glo-1* (**b**) and *cest-4* (**c**) animals. **d**, *cest-4::SL2::GFP::H2B* is expressed in *pah-1*-expressing cells. Expression of *cest-4* was determined using an endogenous GFP reporter (*cest-4(syb5218)[cest-4::SL2::GFP::H2B]*). This reporter was highly expressed in the intestine (yellow circles), and in lower levels in cells in the head, including epidermal cells (green circle), as well as in cells around the vulva and in the tail (blue arrows). Scale bar = 15 μm. **e**, *cest-4* is not expressed in any neurons (red pan-neuronal marker *otIs356[rab-3p(prom1]::2xNLS::TagRFP]*), including HSN. Scale bar = 15 μm. **f**, Relative abundances of sngl#1–4 in WT *C. elegans* in different life stages under fed and starved conditions (*endo-*metabolomes). **g**, Indole biosynthesis in *E. coli* affects serotonin metabolism. Relative abundances of 5-HT, sngl#1–4 in WT *C. elegans* fed indole-deficient *ΔtnaA E. coli* bacteria, with or without indole supplementation, relative to WT *C. elegans* fed the indole-producing parent strain, *E. coli* BW25113. **h**, Proposed model for serotonin metabolism and signaling in *C. elegans*. Data in **b** (n =3), **c** (*cest-4*: n = 4, WT: n = 6), **f** (n = 3), and **g** (n = 7, except for 5-HT measurement, where n = 3) represent biologically independent experiments and bars indicate mean ± s.d., *p-*values calculated by unpaired, two-tailed *t*-test with Welch correction; n.s., not significant.

Next, we investigated the origin of the newly discovered serotonin metabolites, the MOGLs sngl#1–4. ^13^C-labeling of the NAS moiety in MOGLs in animals supplemented with ^13^C-labeled NAS (Fig. 2b) suggested that *N*-acetylation precedes *O*-glucosylation, likely by one of the more than 66 annotated UDP-glucuronosyltransferases (UGTs) in *C. elegans*^38^ (Fig. 5a). Subsequent attachment of the anthranilic acid moiety in sngl#3 and sngl#4 appeared to involve a member of the *c*arboxyl*est*erase (*cest*) family of α,β-hydrolases, which recently have been shown to mediate attachment of diverse moieties in MOGLs identified from *C. elegans* and *C. briggsae*^22,23,39^. For example, *cest-4* is reported to be required for 6-*O*-attachment of anthranilic acid in two MOGLs, iglu#3 and iglu#4 (Fig. 2d)^23^, whereas *cest-1.2* is responsible for the biosynthesis of more than 100 different 2-*O*-acylated MOGLs^22^.

Biosynthesis of all known CEST-derived MOGLs is abolished in mutants of *glo-1*^22,23^, which encodes a Rab GTPase required for the formation of lysosome-related organelles (LROs); cellular compartments that are similar to mammalian melanosomes. Analysis of MS data for the *exo*- and *endo*-metabolomes of *glo-1* mutants showed that biosynthesis of sngl#3 and sngl#4 was abolished, and sngl#2 significantly reduced in *glo-1* mutants, whereas the amount of sngl#1 were increased (Fig. 5b, Supplementary Fig. 5a). Next, we screened the metabolomes of 14 available *cest* knockout mutant strains for changes in the production of sngl#1–4. We found that sngl#3 and sngl#4 were absent in *cest-4* mutants, whereas abundances of serotonin-derived MOGLs were not significantly changed in the other tested *cest* mutants (Supplementary Fig. 5b). We confirmed that that production of sngl#3 and sngl#4 was fully abolished in *cest-4* mutant worms using larger cultures (Fig. 5c). In contrast, sngl#1 levels were largely unchanged and levels of sngl#2, representing a plausible biosynthetic precursor of sngl#3 and sngl#4, were greatly increased in *cest-4* mutant animals compared to WT (Fig. 5c). These results indicate that CEST-4 is required for attachment of the anthranilate moiety to the 6-position of the glucose moieties in sngl#3 and sngl#4, consistent with its previously described role in the biosynthesis of iglu#3 and iglu#4 (Fig. 2d)^23^.

To determine the expression pattern of *cest-4*, we used CRISPR-Cas9 genome engineering to tag the *cest-4* locus with *gfp* (*cest-4*::SL2::GFP::H2B). We observed strong GFP:H2B fluorescence in the intestine, lower levels in head epidermal cells, as well as fluorescence in cells around the vulva and in the tail (Fig. 5d). Given that *pah-1*, *bas-1*, and *cest-4* are all expressed in epidermal cells in the head, biosynthesis of sngl#3 and sngl#4 from PAH-1-derived serotonin could occur cell-autonomously in these tissues, although it is unclear whether *glo-1*-dependent LROs exist in these cells. In contrast, we did not detect *cest-4* expression in any neurons, including TPH-1-dependent serotonin-producing neurons (i.e., NSM, ADF, HSN)^14^ (Fig. 5e). Thus, residual TPH-1-dependent production of sngl#3 and sngl#4 (Fig. 3a) in *pah-1* mutants likely relies on uptake of neuronally-produced serotonin, NAS, or sngl#1/2 by *cest-4*-expressing tissues (e.g. intestine, epidermis).

### Life stage and bacterial food affect serotonin metabolism.

Previous studies showed that the abundances of MOGLs and other neurotransmitter-derived metabolites in *C. elegans* are strongly dependent on life stage and nutritional conditions^20,22^. Examples include the octopamine-derived ascaroside osas#9, a dispersal signal that is primarily produced by starved L1 larvae^20^, and *cest-1.2*-dependent MOGLs incorporating tyramine, which are particularly abundant in starved L3-stage larvae^22^. Comparing the metabolomes of fed and starved animals from the four larval stages and adults, we found that biosynthesis of sngl#1–4 is highly stage-specific and strongly increased under starvation. The *cest-4*-dependent sngl#3 and sngl#4 were nearly undetectable in the L1 and L2 stages and most abundant in L4 larvae and adults, whereas sngl#1 and sngl#2 were also abundant in starved L1 larvae^22^ (Fig. 5f). Levels of the most abundant serotonin glucoside, sngl#4, continued to increase through adulthood (Extended Data Fig. 5c,d).

Lastly, we asked whether microbial tryptophan degradation interacts with the biosynthesis of serotonin metabolites in *C. elegans*, based on our initial finding that supplementation with high concentrations of serotonin starkly affects biosynthesis of indole-derived MOGLs (Fig. 1f). The *Escherichia coli* strain OP50 commonly used as food for *C. elegans* produces large amounts of indole^40^, which is converted into the corresponding glucoside, iglu#1 and then elaborated into the MOGLs iglu#3 and iglu#4 via CEST-4, the same enzyme required for the biosynthesis of the serotonin metabolites sngl#3 and sngl#4, suggesting that serotonin and indole metabolism may interact. First, we confirmed that NAS and sngl#1–4 are not present in bacteria, via targeted HPLC-HRMS analysis of the OP50 metabolome (Supplementary Fig. 5e). Next, we tested whether bacterial indole affects production of serotonin-derived MOGLs. For this purpose, we compared the metabolomes of animals fed with mutant *E. coli* JW3686-7, harboring a disruption in the gene encoding tryptophanase (*ΔtnaA*), which is required for indole (**43**) production (Fig. 5g), and the corresponding indole-producing parent strain, *E. coli* BW25113^41^. HPLC-HRMS analysis showed that abundances of the *cest-4*-dependent MOGLs iglu#3 and iglu#4 were greatly reduced in WT animals grown on *ΔtnaA* bacteria, as expected given the lack of bacterial indole (Supplementary Fig. 5f). In contrast, abundances of *cest-4*-dependent MOGLs incorporating serotonin (sngl#3 and sngl#4) were significantly increased in *C. elegans* fed with a *ΔtnaA* bacteria diet, whereas abundances of putative upstream precursors, NAS, sngl#1, and sngl#2 were reduced (Fig. 5g). Supplementation of worms grown on *ΔtnaA* bacteria with exogenous indole restored levels of NAS and sngl#1–4 to levels observed in worms fed the indole-producing parent strain (Fig. 5g and Supplementary Fig. 5f). These results indicate that bacteria-derived indole modulates *C. elegans* serotonin metabolism, and that, in the absence of indole-derived glucosides, CEST-4 primarily functions in the biosynthesis of the serotonin-derived sngl#3 and sngl#4 (Fig. 5h).

## Discussion

Our results demonstrate that serotonin in *C. elegans* is produced via PAH-1, in addition to canonical hydroxylation of tryptophan via TPH-1. Further, we show that most TPH-1- and PAH-1 derived serotonin is converted into NAS and a novel family of NAS-containing MOGLs, sngl#1–4 (Fig. 5h), which together are far more abundant than free serotonin (Fig. 2f). While the *pah-1*- and *tph-1-*dependent pathways are partially redundant and contribute similar amounts of NAS, sngl#1 and sngl#2, we find that most free serotonin is *tph-1-*derived, whereas *pah-1* contributes more strongly to the further modified metabolites sngl#3 and sngl#4. This result suggests that PAH-1-derived serotonin is converted more rapidly into NAS and sngl#1–4 than TPH-1-derived serotonin. Given that *pah-1*, *bas-1*, and *cest-4* are co-expressed in epidermal cells, PAH-1-dependent serotonin could be converted into sngl#3 and sngl#4 within this tissue. Conversely, because there is no overlap between *tph-1*- and *cest-4*-expressing cells, TPH-1-dependent sngl#3 and sngl#4 likely results from intercellular transport of serotonin, NAS, or sngl#1/2.

In vertebrates, serotonin is produced via tissue-specific tryptophan hydroxylases, TPH1 and TPH2. TPH1 is primarily expressed in a variety of non-neuronal cells, such as the enterochromaffin cells of the gut and the pineal gland, whereas TPH2 is exclusively expressed in neurons in the central nervous system (CNS)^7^. Human phenylalanine hydroxylase, PAH, has been shown to hydroxylate tryptophan *in vitro*, and a recent study reports that PAH contributes to serotonin biosynthesis in mice, suggesting that serotonin biosynthesis via PAH may be widely conserved^30^. We show that *pah-1* and *bas-1* expression overlap in the epidermis, consistent with a model in which *pah-1*-dependent serotonin is produced cell-autonomously in this tissue. However, serotonin biosynthesis could also proceed cell-nonautonomously, e.g. via tryptophan hydroxylation by PAH-1 in glial cells followed by decarboxylation via BAS-1 in the intestine, paralleling vertebrate intestinal serotonin production. A potential role of glial cells in *C. elegans* serotonin production would be interesting given that in vertebrates, glia intimately interact with neurons to regulate neurotransmitter uptake, e.g. serotonin, in the intestine^42^. The *pah-1-*expressing glial cells in *C. elegans* are similar to vertebrate glia and have been shown to affect neuronal development and function^43,44^; however, it is unknown whether glial cells regulate neurotransmitter levels.

Because most endogenously produced serotonin is detected as NAS and MOGLs, we hypothesize that serotonin-dependent phenotypes may be partially mediated by NAS or sngl#1–4. In mice, NAS has been suggested to have anti-depressant^45^, neuroproliferative and neuroprotective^45^ functions. Functions of NAS in *C. elegans* are less well understood; however, the effect of NAS supplementation on exploratory behavior of *bas-1* mutants suggests that NAS or NAS-derived sngl’s contribute to regulating serotonin-dependent behavioral phenotypes (Fig. 4c). In addition, loss of PAH-1 affects egg-laying responses to imipramine. These effects may be mediated by PAH-derived serotonin, although we cannot exclude a possible role of NAS, sngl#1–4, or other PAH-1-dependent metabolites, e.g., dopamine and tyramine.

Given that glycosylation is often reversible^46^, serotonin-derived MOGLs could play a role in trafficking NAS or serotonin between tissues, similar to the proposed role of serotonin glucosides found in plants^47^. Notably, NAS and sngl#1–4 are chemically much less susceptible to oxidative degradation than serotonin, which quickly decomposes under biological conditions^48^. The finding that the carboxylesterase CEST-4 is specifically required for the biosynthesis of sngl#3 and sngl#4 further suggests that these MOGLs may function as secondary messengers of their own. Such novel signaling roles of modular metabolites derived from neurotransmitters have been described previously. For example, osas#9 (Fig. 1b), a modular ascaroside integrating octopamine, is involved in inter-organismal signaling, mediating dispersal of L1 larvae^20^. Like the serotonin derivatives sngl#3 and sngl#4, biosynthesis of osas#9 proceeds via a specific carboxylesterase, CEST-8^39^. Similarly, CEST-1.2 is involved in the biosynthesis of a library of MOGLs integrating tyramine, e.g. tyglu#4 (Fig. 1b)^22^. In contrast to the pheromone osas#9, which is mostly excreted, the serotonin-derived MOGLs sngl#3 and sngl#4 are mostly retained in the worm body, suggesting intra-organismal functions.

Notably, glucosides derived from bacterial indole appear to compete with production of serotonin-derived MOGLs via CEST-4, suggesting that bacterial tryptophan degradation may interact with serotonin-related pathways in *C. elegans*. Similarly, production of *N*-acetylserotonin glucuronide^49^, a mammalian serotonin metabolite reminiscent of sngl#1, has been shown to be influenced by gut microbiota in mice^50^. These results strengthen the notion that microbiota interact with host neurotransmitter metabolism at multiple levels. Taken together, our study reveals unexpected complexity of serotonin biosynthesis and metabolism in *C. elegans*, providing a basis for further exploration of neurotransmitter signaling in this model system.

## ONLINE METHODS

### Nematode and bacterial strains.

Unless indicated otherwise, worms were maintained on Nematode Growth Medium (NGM) 6 cm diameter Petri dish plates with *E. coli* OP50 (http://www.wormbook.org/methods). See Supplementary Table 1 and Supplementary Table 2 for *C. elegans* and *E. coli* strains used in this work.

### Metabolite nomenclature.

All newly detected metabolites for which a structure could be proposed were named using SMIDs. SMIDs (Small Molecule IDentifiers) have been introduced as a search-compatible naming system for metabolites newly identified from *C. elegans* and other nematodes. The SMID database (www.smid-db.org) is an electronic resource maintained in collaboration with WormBase (www.wormbase.org). A complete list of SMIDs can be found at www.smid-db.org/browse.

### Nematode cultures.

Cultures were started by picking *C. elegans* onto 10 cm NGM plates (each seeded with 700 μL of OP50 *E. coli* grown to stationary phase in Lennox Broth). Plates were incubated at 22 °C until most of the food was consumed. Each plate was washed with 25 mL S-complete media into a 125 mL Erlenmeyer flask, with 1 mL of OP50 *E. coli* added (*E. coli* cultures were grown to stationary phase in Lennox Broth, pelleted and resuspended as 1 g wet mass per 1 mL M9 buffer), incubated at 180 RPM and 22 °C for 60 hours. Each liquid culture was transferred to a 50 mL centrifuge tube, added with 6 mL bleach and 900 μL 10 M NaOH, and the mixture was shaken for 3 minutes to prepare eggs. Eggs were centrifuged at 1000 x g for 30 seconds, then the supernatant was removed, and the egg pellet was washed with 25 mL M9 buffer for three times and then suspended in a final volume of 5 mL M9 buffer in a 15 mL centrifuge tube, which would be placed on a rocker and shaken overnight at 22 °C. 100,000 hatched L1 were counted and seeded in 25 mL S-complete media in a 125 mL Erlenmeyer flask, with 1 mL of OP50 *E. coli* added, incubated at 180 RPM and 22 °C. After 70 hours, cultures were centrifuged at 1000 x g for 5 minutes, supernatant media were separated from the worm pellets, which were both snap frozen over liquid nitrogen and lyophilized. Three to seven biological replicates were grown for each strain.

### Metabolite extraction.

Lyophilized pellet samples were homogenized by shaking with 2.5 mm steel balls at 1200 RPM for 3 minutes in 30 seconds pulses, during which samples were cooled with liquid nitrogen (SPEX sample prep miniG 1600). Crushed pellets and media samples were added with 20 mL methanol in 50 mL centrifuge tubes, vortexed vigorously for 30 seconds and then placed on a rocker and shaken overnight at 22 °C to extract metabolites. Extraction mixtures were centrifuged at 5000 x g for 10 minutes at 4 °C, and supernatants were transferred into 20 mL scintillation vials and dried via an SC250EXP SpeedVac (Thermo Fisher Scientific) vacuum concentrator. Dried materials were resuspended in 500 μL methanol, vortexed for 30 seconds and sonicated for 20 minutes. Suspensions were transferred into 1.7 mL Eppendorf tubes and centrifuged at 18,000 x g for 10 minutes at 4 °C. 200 μL extraction supernatant was transferred to HPLC vials and stored at −20 °C until analysis.

### Nematode cultures with Δ*tnaA* bacteria.

25,000 hatched L1 were counted and seeded in 5 mL S-complete media in a 50 mL Erlenmeyer flask, with 1 mL of BW25113 (K12) *E. coli* or JW3686-7 (*ΔtnaA*) *E. coli* added (*E. coli* cultures were grown to stationary phase in Lennox Broth, pelleted and resuspended as 1 g wet mass per 1 mL M9 buffer). The liquid cultures were supplemented with 2 μL of 1.25 M stock of indole in methanol to reach a final concentration of 0.5 mM and incubated at 180 RPM and 20 °C. Control cultures were supplemented with 2 μL of methanol only. After 72 hours, cultures were centrifuged at 1000 x g for 5 minutes, supernatant media were separated from the worm pellets, which were both snap frozen over liquid nitrogen and lyophilized. Seven biological replicates were grown for each strain. Metabolite extraction was performed as in the “Metabolite extraction” section, except for using one fifth of the volumes of extraction solvents.

### Serotonin feeding experiment.

70,000 synchronized wildtype N2 L1 larvae were seeded in 125 mL Erlenmeyer flasks containing 25 mL S-Complete media, with 1 mL of OP50 *E. coli* added, incubated at 180 RPM and 22 °C for 24 hours. The liquid cultures were supplemented with 2 μL of a 12.5 M solution of serotonin hydrochloride in methanol for a final concentration of 5 mM. Control cultures were supplemented with 2 μL of methanol. Worm cultures were incubated at 180 RPM and 22 °C for another 46 hours. Worm cultures were centrifuged at 1000 x g for 5 minutes, and supernatant media were separated from the worm pellets, which were both snap frozen over liquid nitrogen and lyophilized. Dried samples were extracted as above.

### ^13^C-NAS isotope tracing experiment.

70,000 synchronized wildtype N2 L1 larvae were seeded in 125 mL Erlenmeyer flasks containing 25 mL S-Complete media, with 1 mL of OP50 *E. coli* added, incubated at 180 RPM and 22 °C for 24 hours. The liquid cultures were supplemented with 2 μL of a 12.5 mM solution of either NAS in methanol or ^13^C-NAS in methanol, for a final concentration of 5 μM. Control cultures were supplemented with 2 μL of methanol. Worm cultures were incubated at 180 RPM and 22 °C for another 46 hours. Worm cultures were centrifuged at 1000 x g for 5 minutes, supernatant media were separated from the worm pellets, which were both snap frozen over liquid nitrogen and lyophilized. Dried samples were extracted as above.

### Preparation of *endo*-metabolome samples from staged starved and fed *C. elegans*.

Samples were prepared following a previously reported procedure^22^. 40,000 synchronized L1 larvae were added to 125 mL Erlenmeyer flasks containing 30 mL of S-complete medium. Worms were fed with 4 mL of concentrated OP50 and incubated at 20 °C with shaking at 160 RPM for: 12 hours (L1), 24 hours (L2), 32 hours (L3), 40 hours (L4) and 58 hours (gravid adults). For preparation of starved samples, each of the larval and adult stages was starved for 24 hours after reaching their desired developmental stage in S-complete media without OP50. After incubation for the desired time, liquid cultures were centrifuged (1000 x g, 22 °C, 1 min) and supernatants were collected. Intact OP50 were removed from the supernatant by centrifuging (3000 x g, 22 °C, 5 min), and the resulting supernatants (containing the *exo*-metabolomes) were lyophilized. Lyophilized samples were homogenized with a dounce homogenizer in 10 mL methanol and extracted on a stirring plate (22 °C, 12 h). The resulting suspension was centrifuged (4000 x g, 22 °C, 5 min) to remove any precipitate and then carefully transferred to HPLC vials. Three biological replicates were started on different days.

### Preparation of *endo*-metabolome samples from *C. elegans* at different adult ages.

For all adult samples, 90,000 synchronized L1 animals of wildtype *C. elegans* were seeded in 125 mL Erlenmeyer flasks containing 25 mL S-Complete media with added *E. coli* OP50 and incubated at 20 °C, 180 RPM. Day 1 adult animals were extracted 72 hours after initial seeding. Worm cultures were centrifuged at 1000 x g, room temperature for 1 minute, then 1/3 of the pellet (approximately 30,000 animals) were frozen on dry ice and stored at −20 °C until further process. The remaining 2/3 of worm culture was placed back in liquid culture. Day 5 and day 7 animals (30,000 animals each, half of the pellet) were harvested similarly to day 1 animals. To avoid larval contamination due to egg laying, larvae were removed daily. For this purpose, cultures were centrifuged at 1000 x g and 20 °C for 1 min. The supernatant was removed, and the pellet was washed with M9 media until clear of larvae. The pellet was placed back in S-complete supplemented with fresh *E. coli* OP50. Worm samples were collected and extracted as above. Samples were dissolved in 75 μL of methanol for MS analysis. Three biological replicates were started on different days.

### Mass spectrometric analysis.

High resolution LC-MS analysis was performed on a Thermo Fisher Scientific Vanquish Horizon UHPLC System coupled with a Thermo Q Exactive hybrid quadrupole-orbitrap high-resolution mass spectrometer equipped with a HESI ion source, using Thermo Scientific Xcalibur (v4.3.73.11). 1 μL of extract was injected and separated using a water-acetonitrile gradient on a Thermo Scientific Hypersil GOLD C18 column (150 mm × 2.1 mm, 1.9 μm particle size, 175 Å pore size, Thermo Scientific) and maintained at 40 °C unless otherwise stated. Solvents were all purchased from Fisher Scientific as Optima grade. Mass spectrometer parameters: 3.5 kV spray voltage, 380 °C capillary temperature, 300 °C probe heater temperature, 60 sheath flow rate, 20 auxiliary flow rate, 2.0 spare gas; S-lens RF level 50.0, resolution 240,000, *m/z* range 150–1000, AGC target 3e6. Instrument was calibrated with positive and negative ion calibration solutions (Thermo Fisher) Pierce LTQ Velos ESI^+^ and ESI^−^ calibration solutions. Peak areas were determined using Xcalibur 4.1 QualBrowser (v4.1.31.9, Thermo Scientific) using a 5 ppm window around the *m/z* of interest. MS Excel (v2112) and Graphpad PRISM (v8.4.0) were used to plot and perform statistical analysis. HPLC-MS peak areas were normalized to the measured abundance of ascr#3 (https://smid-db.org/detail/ascr#3/), in each sample for all graphs in this study, except for the measurement of neurotransmitters via derivatization. For quantitative analyses, compound loss during sample processing (see below) and ion suppression in the worm metabolome matrix was taken into account. For measurement of NAS (**5**), the peaks for the sodium adduct at *m/z* 241.0947 in ESI+ mode were used, because of interference of co-eluting metabolitesthat have the same *m/z* 219.1128 as the proton adduct. For analysis with the Hypersil GOLD C18 column (for HILIC conditions, see further below), two different methods were used, as follows:

28-minute mass spectrometric analysis method: Solvent A: 0.1% formic acid in water; solvent B: 0.1% formic acid in acetonitrile. A/B gradient started at 1% B for 3 min, then from 1% to 100% B over 20 min, 100% for 5 min, then down to 1% B for 3 min.

73-minute mass spectrometric analysis method: Solvent A: 0.1% formic acid in water; solvent B: 0.1% formic acid in acetonitrile. A/B gradient started at 1% B for 3 min, then from 1% to 100% B over 20 min, 100% for 5 min, then down to 1% B for 3 min.

### Feature detection and characterization.

LC-MS RAW files from each sample were converted to mzXML (centroid mode) using MSConvert (ProteoWizard, v3.0.18250-994311be0), followed by analysis using the XCMS analysis feature in METABOseek (metaboseek.com, v0.9.7)^24^. Peak detection was carried out with the centWave algorithm, values set as: 4 ppm, 320 peakwidth, 3 snthresh, 3100 prefilter, FALSE fitgauss, 1 integrate, TRUE firstBaselineCheck, 0 noise, wMean mzCenterFun, −0.005 mzdiff. XCMS feature grouping values were set as: 0.2 minfrac, 2 bw, 0.002 mzwid, 500 max, 1 minsamp, FALSE usegroup. METABOseek peak filling values set as: 5 ppm_m, 5 rtw, TRUE rtrange. Resulting tables were then processed with the METABOseek Data Explorer. Molecular features were filtered for each particular null mutant against all other mutants. Filter values were set as: 10 to max minFoldOverCtrl, 15000 to max meanInt, 120 to 1500 rt, 0.95 to max Peak Quality as calculated by METABOseek. Features were then manually curated by removing isotopic and adducted redundancies. Remaining masses were put on the inclusion list for MS/MS (ddMS/MS) characterization. Positive and negative mode data were processed separately. In both cases we checked if a feature had a corresponding peak in the opposite ionization mode, since fragmentation spectra in different modes often provide complementary structural information. To acquire MS/MS spectra, we applied method on a Thermo QExactive-HF mass spectrometer with MS1 resolution 60,000, AGC target 1e6, maximum IT (injection time) 50 ms, MS/MS resolution 45,000, AGC target 5e5, maximum IT 80 ms, isolation window 1.0 m/z, stepped NCE (normalized collision energy) 10, 30, dynamic exclusion 3 s.

### HILIC-MS analysis of sngl#1 and sngl#3 using a HILIC column.

Analyses were performed using the instrumentation described in the ‘Mass spectrometric analysis’ section. 1 μL of synthetic samples or metabolome extract were separated using a water-acetonitrile gradient on an Agilent Zorbax Hilic Plus column (150 mm × 2.1 mm, particle size 1.8 μm) maintained at 40 °C with a flow rate of 0.3 mL/min. Solvent A: 0.1% formic acid in water; Solvent B: 0.1% formic acid in acetonitrile. Analytes were separated using a gradient profile as follows: 2 min (95% B) → 20 min (50% B) →20 min (50% B) lvent A: 0.1% formic acid in water; Solvent B: 0.1% Samples was analyzed in ESI+ mode, *m/z* range 100 to 700.

### HPLC-MS analysis of sngl#2 and sngl#4 using an amide HILIC column.

Analyses were performed using the instrumentation described in the ‘Mass spectrometric analysis’ section. 1 μL of synthetic sample or metabolome extract were separated using a water-acetonitrile gradient on a Waters Xbridge Amide column (150 mm × 2.1 mm, particle size 3.5 μm) maintained at 40 °C. Solvent A: 0.1% ammonium formate in 90% acetonitrile-10% water; Solvent B: 0.1% ammonium formate in 30% acetonitrile-70% water. Analytes were separated using a gradient profile as follows: started at 1% B for 3 min, linearly increasing to 25% B over 17 min, 100% B over 3 min, holding at 100% B for 4.9 min, then down to 1% B in 0.1 min and holding at 1% B for 2 min. Samples were analyzed in ESI− mode, *m/z* range 70 to 1000.

### sngl#2 isolation and characterization.

300,000 synchronized wildtype (N2) L1 larvae were seeded in a 500 mL Erlenmeyer flask containing 100 mL S-complete media, with 1 mL of OP50 *E. coli* added, and incubated at 180 RPM and 22 °C for 72 hours. The liquid culture was transferred into a 2 L Erlenmeyer flask containing 400 mL S-complete media, with 10 mL of OP50 *E. coli* and 53 mg of serotonin hydrochloride added, resulting in a final concentration 0.5 mM of serotonin in the liquid culture. After incubated at 180 RPM and 22 °C for 70 hours, the culture was centrifuged at 1000 x g for 5 min, and the worm pellets were separated from the supernatant media and lyophilized to dryness. Dried worm pellet samples were ground and extracted as above. After extraction using methanol overnight, extraction mixtures were centrifuged at 5000 x g for 10 min at 4 °C. Extracts were combined into a 500 mL round bottom flask, 2.1 g celite was added, and the resulting suspension was concentrated to dryness *in vacuo*, resulting in a beige powder, representing worm *exo*-metabolome absorbed on celite. This sample was fractionated using C18 reversed-phase flash chromatography using a gradient of 0–100% ACN in H_2_O with 0.1% formic acid. Fractions were checked by HPLC-MS, for the presence of sngl#2, and sngl#2-containing fractions were combined and concentrated to dryness *in vacuo*. The residue was further fractionated on a Thermo Fisher Scientific Vanquish Horizon UHPLC System coupled with a Thermo Q Exactive hybrid quadrupole-orbitrap high-resolution mass spectrometer equipped with a HESI ion source, using a Phenomenex Luna^®^ C18 column (250 × 4.6 mm, 5 μm particle diameter, 100 Å pore size), using a 0.1% aqueous formic acid-acetonitrile solvent gradient at a flow rate of 4.0 mL/min; solvent A: 0.1% formic acid in water; solvent B: 0.1% formic acid in acetonitrile. The gradient started at 1% B for 13.83 min, then from 1% to 50% B over 140 min, then from 50% to 98% B over 0.5 min, holding at 98% for 18 min, then back down to 1% B in 1 min, holding for 9 min. Fractions containing sngl#2 were concentrated *in vacuo*, and the residue was subjected to NMR spectroscopic analysis (CD_3_OD, Bruker AVANCE III HD, 800 MHz).

### Recovery experiment for quantitation of serotonin-derived metabolites.

Batches of 70,000 synchronized L1 of *pah-1(syb3596);tph-1(mg280)* animals were counted and seeded in 25 mL S-complete media in a 125 mL Erlenmeyer flask, with 1 mL of OP50 *E. coli* added, and incubated at 180 RPM and 22 °C. After 70 hours, cultures were centrifuged at 1000 x g for 5 minutes, supernatant media were separated from the worm pellets. 50 μL of 10 μM stock solution of synthetic NAS, sngl#1, sngl#2, sngl#3, and sngl#4 in methanol were added to pellet samples, which were then snap frozen over liquid nitrogen, lyophilized, extracted, and analyzed by HPLC-HRMS in the same manner as described above, which, assuming 100% recovery, would result in metabolome extract containing the added synthetic compounds at a concentration of 1 μM. Percent recovery was assessed based on comparison of MS peak intensities of this sample to a control sample of unsupplemented *pah-1(syb3596);tph-1(mg280)* extract, to which, immediately prior to MS analysis, the same set of synthetic serotonin metabolites were added to reach a concentration of 1 μM. Ion suppression due to the matrix of co-eluting worm metabolites was assessed separately by comparing MS peak intensities obtained for a 1 μM solution of synthetic serotonin metabolites (without added worm metabolome) with peak intensities obtained for the sample of unsupplemented *pah-1(syb3596);tph-1(mg280)* extract, to which, immediately prior to MS analysis, synthetic serotonin metabolites were added to reach a concentration of 1 μM. Quantitative data for the abundances of serotonin metabolites (Figure 2f) were calculated taking into account average percent recovery and ion suppression (Supplementary Fig. 4a,b).

### Derivatization for serotonin detection.

100,000 synchronized L1 larvae were seeded in 125 mL Erlenmeyer flasks containing 25 mL S-complete media, with 1 mL of OP50 *E. coli* added, and incubated at 180 RPM and 22 °C. After 70 hours, cultures were centrifuged at 1000 x g for 5 minutes, and supernatant media were discarded. Worm pellets were washed three times with 1 X PBS, then transferred to 2.5 mL Eppendorf tubes and washed with 0.1 X PBS, snap frozen over liquid nitrogen, and stored at −80 °C until further sample preparation. Four to eight biological replicates were grown for each strain. Control samples were prepared by adding 20 μL of 1 mg/mL serotonin hydrochloride methanol stock to serotonin-deficient *pah-1(syb3596);tph-1(mg280)* worm pellet samples (to assess percent recovery, as described above for serotonin metabolites) or 200 μL 1 X PBS buffer (to assess the effect of additional ion suppression due to worm metabolome background) before snap freezing. Worm pellets were lyophilized to dryness, then homogenized by shaking with 2.5 mm steel balls at 1200 G for 3 minutes in 90 seconds pulses, during which samples were cooled with liquid nitrogen (SPEX sample prep miniG 1600). Carbonate buffer (Na_2_CO_3_/NaHCO_3_, 100 mM, pH 12, adjusted using 10 mM NaOH) and 14.8 mM dansyl chloride solution (in acetone, prepared and stored in the dark) were freshly prepared. To each pellet sample was added 150 μL carbonate buffer, the pH was adjusted with 1 M NaOH until the mixture reached pH 12, and 150 μL dansyl chloride solution was added. Mixtures were vortexed vigorously for 30 s and incubated in a water bath at 60 °C. After 15 minutes, to each tube was added 5 μL 15% formic acid to adjust pH to 7, and the samples were centrifuged at 15,000 x g for 5 minutes at 4 °C. Reaction mixtures were transferred to HPLC vials and lyophilized. Dried samples were resuspended in 80 μL methanol and ethyl acetate (1:1 v/v), vortexed for 30 seconds, and sonicated for 15 minutes. Samples were transferred to appropriated HPLC inserts and centrifuged at 2,000 x g for 1 minute. Supernatants were transferred to new HPLC vials and analyzed on the same day. 3 μL of extract was injected and separated using a water-acetonitrile gradient on a Thermo Scientific Hypersil GOLD C18 column. Mass spectrometric analysis was conducted using the 28-min HPLC method described above. Both mono- and di-dansyl derivatized serotonin were quantified in each sample.

### RNAi experiments.

Bacteria expressing *cat-2*, *tph-1*, *pah-1* dsRNA (Ahringer library) and control vector (*L4440*) was grown on LB/carbenicillin (50 μg/mL) /tetracycline (50 μg/mL) agar plates at 37 °C overnight. A single colony of bacteria was inoculated in LB/ampicillin (50 μg/mL) liquid media and grown for 8–10 hours. RNAi plates were prepared by supplementing NGM plates with IPTG (1 mM)/ampicillin (50 μg/ml), and 500 μL of the previously grown RNAi bacteria was added to each plate. Seeded plates were dried at room temperature for 24 hours. L4 animals (P_0_) were placed onto RNAi plates (control (*L4440*) and treatment (*cat-2*, *tph-1*, *pah-1*)) and kept at 20 °C. 10 L4 F_1_ worms were transferred to fresh 10 cm RNAi plates (5 plates for each treatment), and worms were grown for 4–5 days until obtaining a mixed population of F_2_ worms. Each set of 5 plates was washed twice with 6 mL of M9 buffer, and the plate washes were centrifuged, the supernatants discarded, and the worm pellets washed twice with M9 twice and then frozen at −80 °C. Three biological replicates were grown for each condition. To the lyophilized pellet samples was added 1 mL methanol, and the samples were homogenized by sonication (2s pulse, 2s off) for 3 minutes. Samples were placed on a rocker and shaken for 1 hour and then centrifuged at 10,000 x g for 10 minutes at 4 °C. The supernatants were transferred into 4 mL scintillation vials. Another 1 mL methanol was added and the extraction procedure was repeated. Combined extractions were dried using an SC250EXP SpeedVac (Thermo Fisher Scientific) vacuum concentrator. Dried materials were resuspended in 100 μL methanol, vortexed for 30 seconds and sonicated for 20 minutes. Suspensions were transferred into 1.7 mL Eppendorf tubes and centrifuged at 18,000 x g for 10 minutes at 4 °C. 100 μL extraction supernatant was transferred to HPLC vials, evaporated again, and resuspended in 20 μL methanol for further concentration. Metabolite extract samples were stored at −20 °C until HPLC-MS analysis.

### RT-PCR.

Samples of around 500 worms from each RNAi treatment were collected and frozen. Total RNA was extracted and purified using Trizol and RNA Clean & Concentrator^™^-5 kit (ZYMO Research). cDNA was synthetized using the SuperScript III First-Strand kit (Invitrogen). qPCR was performed using SYBR green dye (Thermo Fisher Scientific) on a BIO-RAD C1000^™^ Thermal Cycler. Relative gene expression levels were calculated using the 2^−ΔΔCt^ method with *ama-1* as a house keeping gene^51^. Two technical replicates were performed for each strain and treatment, per biological replicate. See Supplementary Table 3 for primer sequences.

### Microscopy.

One day old hermaphrodites were anesthetized using 25 mM sodium azide and mounted on 5% agarose pads on glass slides. Images were acquired at 40x using a confocal laser scanning microscope (Zeiss LSM 880) and processed using ImageJ software^52^. For each strain, representative maximum projections are shown.

### Egg laying assay.

Egg laying assays performed in this study were based on previously described assays^19^. Briefly, individual one-day, or two-day old adults were each transferred into a 96 well microtiter plate containing 100 μL of solution. Number of eggs released per animal were scored after 120 minutes at room temperature. Concentration of drugs in M9 buffer were as follows: 0.5 mg/mL fluoxetine hydrochloride and 0.75 mg/mL imipramine hydrochloride^53^. Controls in M9 Buffer alone were performed on each strain every time. Each assay tested eight animals/strain/treatment and was repeated three independent times. Animals injured during transfer were excluded from analysis.

### Locomotory behavior.

Automated single worm tracking was performed using the WormTracker v2.0 system^36^ at room temperature. One day old, fed adult hermaphrodites were recorded for three minutes on an NGM plate fully coated with a thin layer of OP50. Data were collected on three independent days. All strains were recorded on each experimental day. Data were combined and analyzed using the Worm Tracker v2.0 software (https://www.mrc-lmb.cam.ac.uk/wormtracker/). In addition to statistical testing performed by Worm Tracker v2.0 software, significant features against wildtype N2 (q-value(Wilcoxon)≤0.05) were further corrected for multiple comparisons using One Way ANOVA, Tukey’s posthoc test.

### Exploratory behavior assay.

To measure exploratory behavior, we employed a previously described assay setup^37^. Briefly, 35 mm NGM plates were fully covered with OP50 and allowed to dry to for 2–3 days. One young L4 animal was transferred to each plate. After either 6 or 13.5 hours, the worms were removed from the plates and the plates were superimposed on a grid containing 3.5 mm squares, and the number of squares entered (max. 88) by each worm was manually counted. All worms were incubated at 20 °C. For each assay 20 animals per group and 2 replicates were analyzed. Statistical significance was determined using One Way ANOVA with posthoc Tukey’s.

### Exploratory behavior assay with *N*-acetylserotonin supplementation.

We employed the same assay setup as above, with the following modifications. 400 μM and 40 mM NAS stock solutions were freshly prepared using DMSO/H_2_O (v/v = 1:1). 35 mm NGM plates were covered with 100 μL NAS stock solution and allowed to diffuse overnight into the whole plate. Control plates were covered with 100 μL DMSO/H_2_O solution (v/v = 1:1). Then plates were fully covered with OP50 and allowed to dry overnight. One young L4 *bas-1(ad446)* animal was transferred to each plate and incubated at 20 °C. After 13.5 hours, the worm was removed and the plates were superimposed on a grid containing 4.0 mm squares, and the number of squares entered (max. 72) by each worm was manually counted. For each assay 10–12 animals per group were analyzed, for each of the three independent replicates. Statistical significance was determined using One Way ANOVA with posthoc Tukey’s.

### Statistical analysis.

HPLC-MS peak areas were normalized to the measured abundance of ascr#3 (https://smid-db.org/detail/ascr#3/), in each sample for all graphs in this study, except for the measurement of neurotransmitters via derivatization. Peak integration data from HPLC-MS analysis were then normalized to WT (N2) average from each batch, and log-transformed prior to statistical analysis. Significance of differences between peak areas were assessed using unpaired, two-tailed *t*-tests, except for data presented in Figure panels 4b-e and Supplementary Figure 4, which were analyzed using one-way ANOVA with Tukey’s post-hoc test.

## Extended Data

**Extended Data Fig. 1 |:**
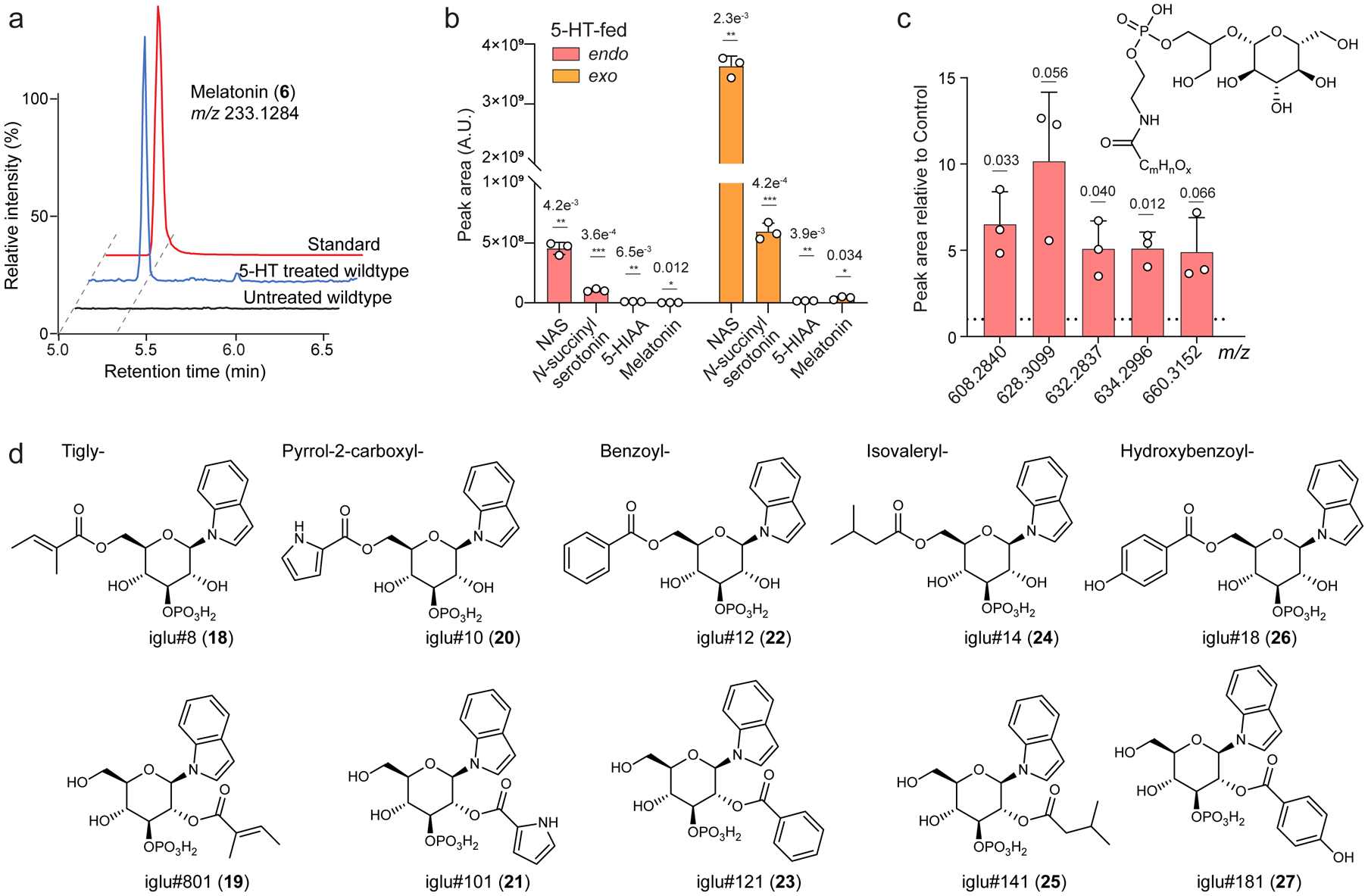
Metabolic changes with 5 mM serotonin treatmentin *C. elegans*. **a**, ESI+ ion chromatograms for melatonin synthetic standard and WT *C. elegans endo*-metabolome extracts. Melatonin was only detected in WT treated with an unphysiologically high concentration (5 mM) of serotonin but was not detected in untreated WT animals. **b**, Relative abundances of known serotonin metabolites in *exo*- and *endo*-metabolome samples of WT treated with 5 mM serotonin. **c**, Abundances of *N*-acyl-glycoglycerophosphoethanolamines (GLEAs) in *endo*-metabolomes of WT treated with 5 mM serotonin. 15 ≤ m ≤ 19, 26 ≤ n ≤ 30, x = 0 or 1. **d**, Structures of indole-derived MOGLs whose abundances were decreased in WT treated with 5 mM serotonin. Data in **b** and **c** represent 3 biologically independent experiments and bars indicate mean ± s.d., *p*-values calculated via unpaired, two-tailed *t*-test with Welch correction.

**Extended Data Fig. 2 |:**
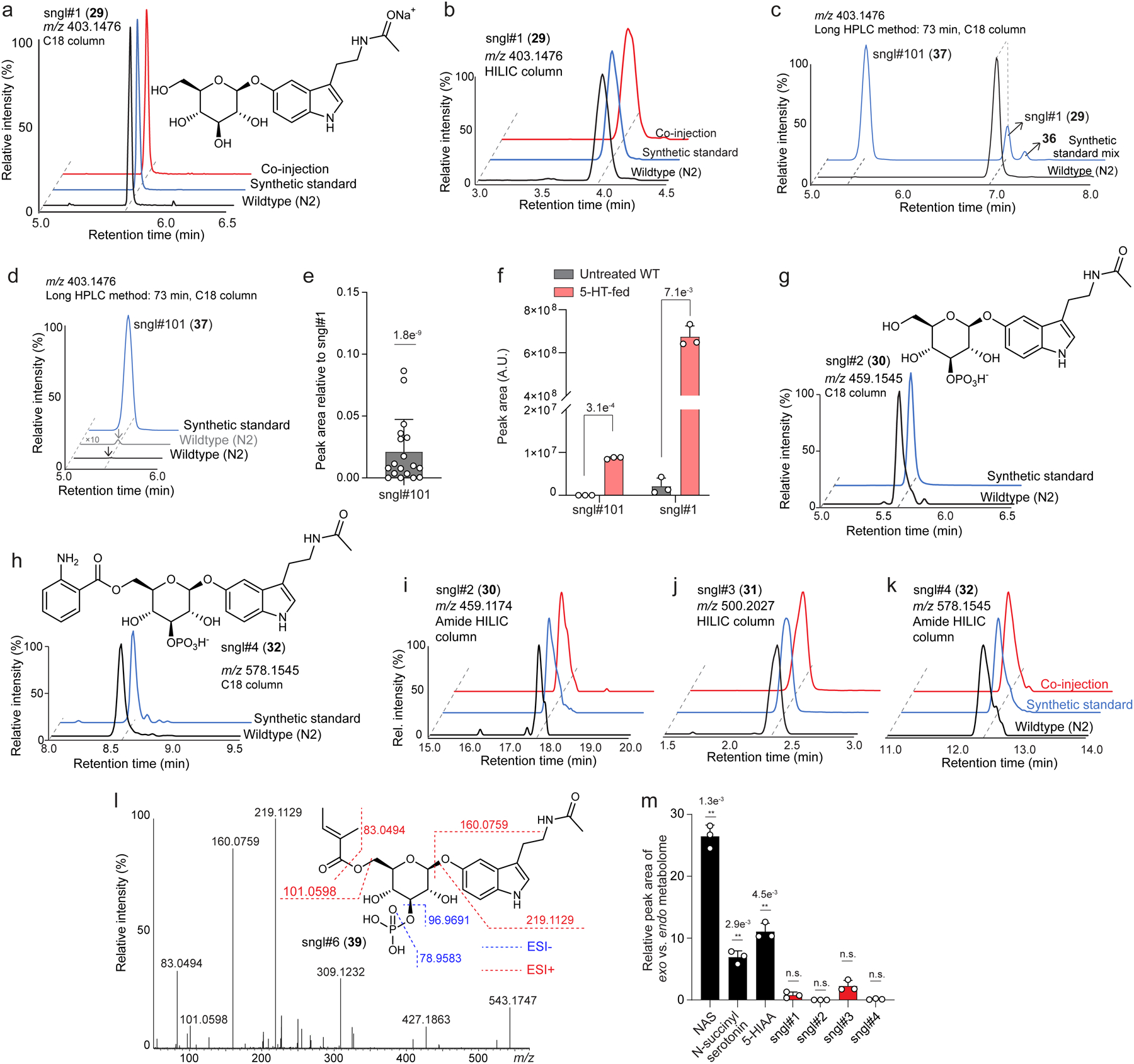
**Identification of serotonin-derived glucosides. a**,ESI+ ion chromatograms for sngl#1 in WT, synthetic standards, and for co- injections of natural and synthetic samples. **b**, ESI+ ion chromatograms for sngl#1 (HILIC column) in *endo*-metabolome of WT and synthetic standard. **c**, ESI+ ion chromatograms for sngl#101, sngl#1 and **36** in WT and synthetic standards, indicating that the β-*O*-linked serotonin glucoside (sngl#1) was the by far major isomer, compared to α-*O*-linked **36**. **d**, ESI+ ion chromatogram for sngl#101 in WT and synthetic standard. Y-axis for *m/z* 403.1476 was scaled 10-fold relative to chromatogram shown in panel **c** to highlight presence of trace amounts of sngl#101. **e**, Abundance of β-*N*- linked sngl#101 relative to β-*O*-linked sngl#1 in WT. **f**, Abundances of sngl#101 and sngl#1 in WT treated with 5 mM serotonin. **g**, **h**, ESI− ion chromatograms for sngl#2 (**g**) and sngl#4 (**h**) in *endo*-metabolomes of WT and synthetic standards using a C18 column. **i**-**k**, Chromatograms for sngl#2 (Amide HILIC column, ESI−), sngl#3 (HILIC column, ESI+) and sngl#4 (Amide HILIC column, ESI−) in *endo*-metabolomes of WT and synthetic standards. **l**, ESI+ MS/MS spectrum and proposed fragmentation of sngl#6. **m**, Relative abundance of serotonin derived metabolites in *endo*- and *exo*-metabolomes of unsupplemented WT. Data in **e** (n = 18), **f** (n = 3) and **m** (n = 3) represent biologically independent experiments and bars indicate mean ± s.d., *p*-values calculated by unpaired, two-tailed *t*-test with Welch correction.

**Extended Data Fig. 3 |:**
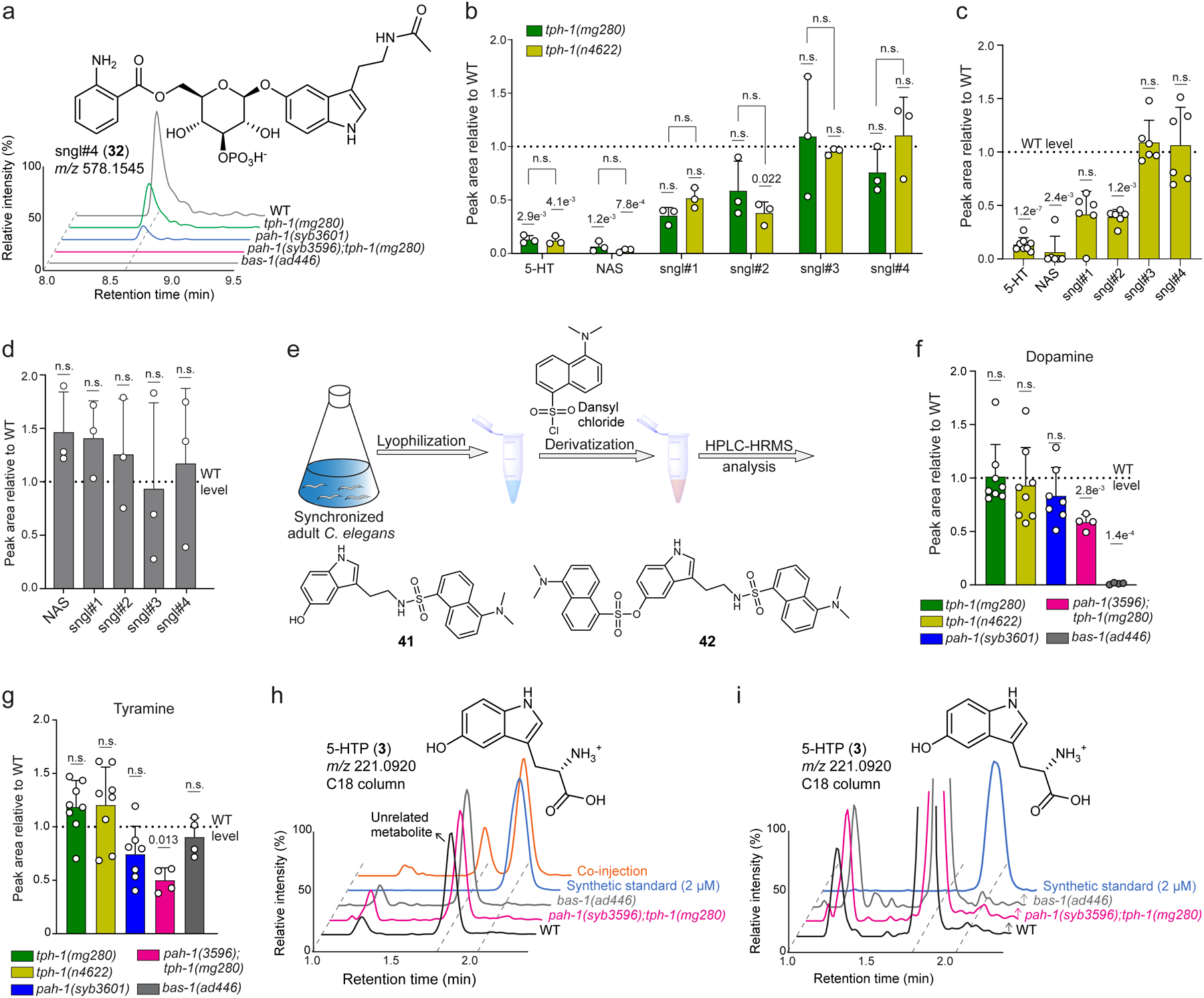
Metabolomic analysis of serotonin biosynthesis mutants. **a**, sngl#4 biosynthesis was abolished in *pah-1(syb3596);tph- 1(mg280)* and *bas-1(ad446)* but not *tph-1(mg280)* or *pah-1(syb3601)* mutants. **b**, Abundances of serotonin derivatives in two *tph-1* alleles, *tph-1(mg280)* and *tph-1(n4622)*, grown in parallel replicates. **c**, Relative abundances of serotonin derivatives in *tph-1(n4622) endo*-metabolome samples. **d**, Relative abundances of serotonin derivatives in *cat-2(e1112) endo*-metabolome samples. **e**, Scheme for quantitation of free serotonin via derivatization. **f**, **g**, Relative abundances of neurotransmitters dopamine (**f**) and tyramine (**g**) in WT, *tph-1(mg280)*, *tph- 1(n4622)*, *pah-1(syb3601)*, and *pah-1(syb3596);tph-1(mg280) endo*-metabolomes. Data in **b** (n = 3), **c** (n = 6), **f** and **g** (*tph-1(mg280)*: n = 8, *tph-1(n4622)*: n = 8, *pah-1(syb3601)*: n = 7, *pah-1(syb3596);tph-1(mg280)*: n = 4, *bas-1(ad446)*: n = 4) represent biologically independent experiments and bars indicate mean ± s.d., *p-*values calculated via unpaired, two-tailed *t*-test with Welch correction, comparing mutant animals and WT. **h**, ESI+ ion chromatograms for *m/z* 221.0920 for *bas-1(ad446)* mutant, *pah-1(syb3596);tph-1(mg280)* double mutant, and WT *endo*-metabolomes as well as a 2 μM 5-HTP synthetic standard and co-injection with WT *endo*-metabolome. **i**, Enlargement of ESI+ ion chromatograms for *m/z* 221.0920 for *bas-1(ad446)*, *pah-1(syb3596);tph-1(mg280)* double mutant, and WT *endo*-metabolomes as well as 50 nM synthetic standard of 5-HTP. Neither of the shown *endo*-metabolome chromatograms shows a significant peak for 5-HTP.

**Extended Data Fig. 4 |:**
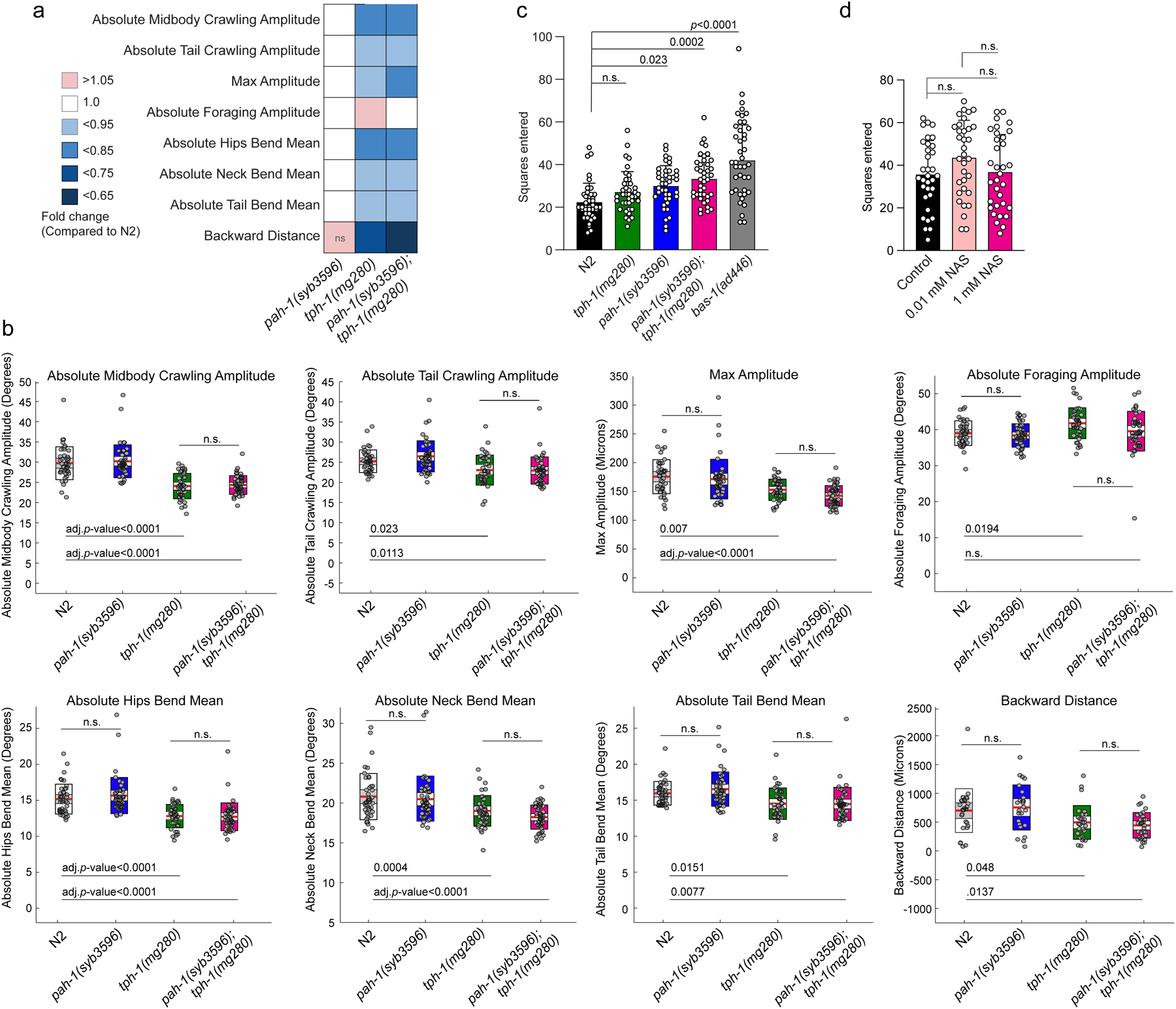
Serotonin-related behaviors of different mutants. **a**, Heat map of locomotory behaviors of *pah-1(syb3601)*, *tph-1(mg280)*, *pah- 1(syb3596);tph-1(mg280)* compared to WT. Fed day-1 adult animals were tracked using an automated single worm tracker. *pah-1(syb3601)* mutant animals (n = 46) did not show any gross defects in locomotion compared to WT. Features significantly different (q-value(Wilcoxon) <0.05) between WT (n = 42) and *tph-1(mg280)* (n = 40) mutant animals are displayed as fold change in relation to WT (red: upregulated compared to WT; blue: downregulated compared to WT). Except for foraging, *pah-1(syb3596);tph-1(mg280)* double mutants (n = 47) display significant changes in the same locomotory features compared to WT as observed between WT and *tph-1(mg280)* animals. n.s., not significant. **b**, Loss of PAH-1 did not result in gross locomotory defects. Fed day 1 adult animals were tracked using an automated single worm tracker. *pah-1(syb3601)* mutant animals did not show any gross defects in locomotion compared to WT. Features significantly different (q-value (Wilcoxon) <0.05) between WT and *tph-1(mg280)* mutant animals are displayed as boxplots with individual values for each animal tested. Additional loss of PAH-1 does not exacerbate phenotypes observed in *tph-1(mg280)* single mutants. Boxplots were generated using Worm Tracker (v2.0). Red center line indicates mean with 95 % confidence intervals (white box), outer boxes indicate standard deviation. **c**, Loss of PAH-1 significantly increased exploratory behavior after 6 h, to a larger extent than loss of TPH-1. **d**, Exogenous NAS did not influence exploratory behavior of WT after 13.5 h. Datain **c** (n = 20 per experiment per genotype and treatment) were collected in 2 biologically independent experiments. Data in **d** (n = 10 and n = 11 for the first two experiments and n = 12 for the third experiment) were collected in 3 biologically independent. Statistics in **b**-**d** were calculated using One-Way-ANOVA and *p*-values were adjusted with posthoc Tukey’s multiple comparison test. n.s., not significant.

**Extended Data Fig. 5 |:**
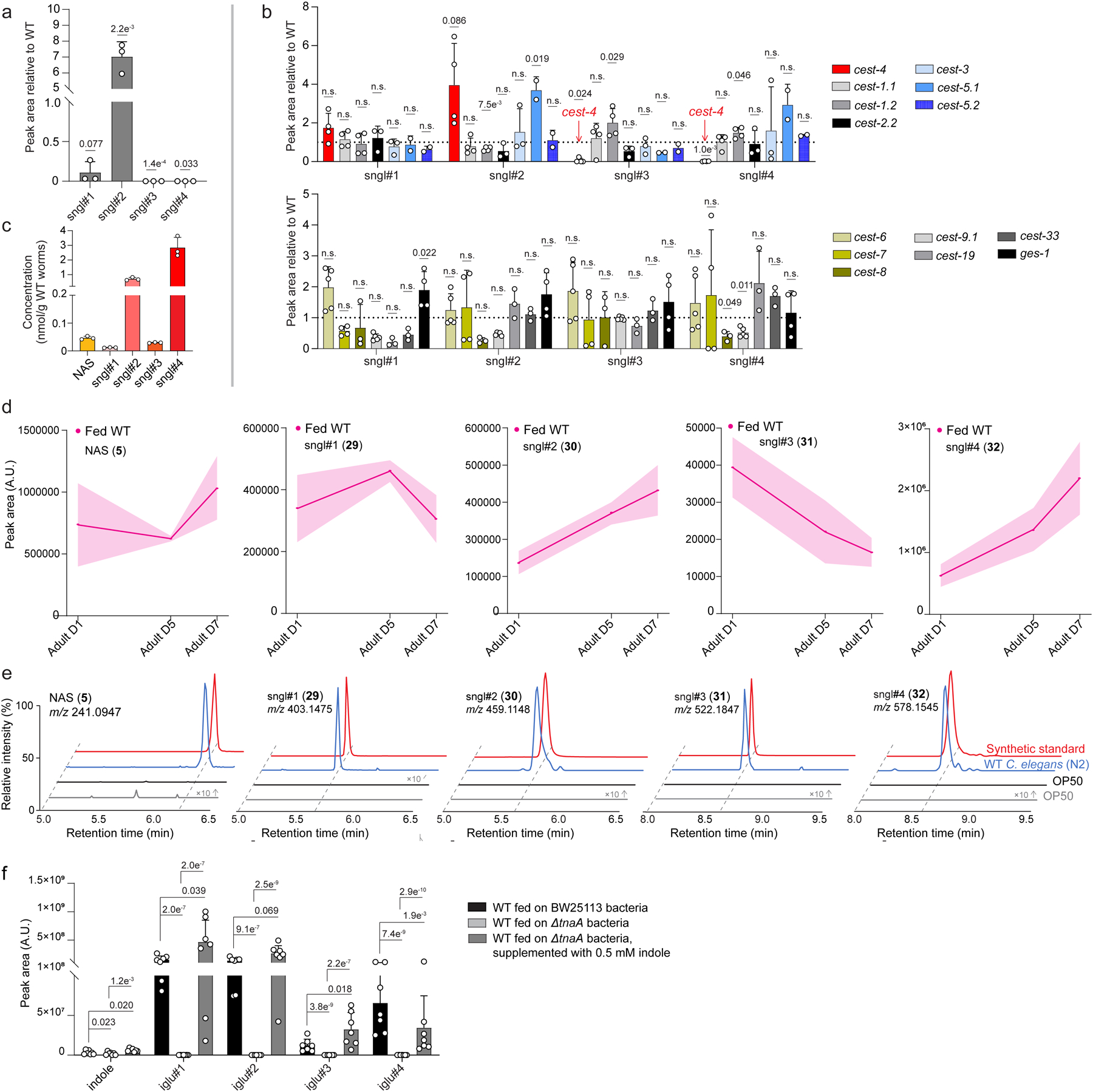
Life stage, starvation, and microbial metabolism affect serotonin metabolite biosynthesis. **a**, Abundances of sngl#1–4 in *exo*- metabolomes of *glo-1* mutants relative to WT. **b**, Abundances of sngl#1–4 in *cest* mutants (*cest-4*, *cest-1.1*, *cest-1.2*, *cest-2.2*, *cest-3*, *cest-5.1*, *cest-5.2*, *cest-6*, *cest-7*, *cest-8*, *cest-9.1, cest-19*, *cest-33*, *ges-1*) relative to WT. n.d., not detected. **c**, Absolute concentrations of NAS, sngl#1, sngl#2, sngl#3, and sngl#4 in WT *C. elegans* at day 7 of adulthood. **d**, Abundances of sngl#1–4 in the *endo*-metabolomes of WT *C. elegans* at day 1, day 5, and day 7 of adulthood. **e**, ESI+ and ESI− ion chromatograms for NAS and sngl#1–4 in *endo*-metabolomes of *E. coli* OP50 and WT *C. elegans*, demonstrating that NAS and serotonin glucosides sngl#1–4 are not produced by OP50. **f**, Abundances of free indole and iglu#-family metabolites in *endo*- metabolome samples of WT *C. elegans* fed BW25113 or *ΔtnaA E. coli* bacteria. Data in **a** (n = 3), **b** (n = 2–4), **c** (n = 3), **d** (n = 3), and **f** (n = 7) represent biologically independent experiments, and bars indicate mean ± s.d., *p-*values calculated by unpaired, two-tailed *t*-test with Welch correction; n.s., not significant.

## Supplementary Material

Source Data Extended Data Figure 1

Source Data Extended Data Figure 3

Source Data Extended Data Figure 2

Source Data Extended Data Figure 4

Source Data Extended Data Figure 5

Source Data Figure 1

Source Data Figure 2

Source Data Figure 3

Source Data Figure 4

Source Data Figure 5

Supplementary Information

## Data Availability

Source data are provided with this paper. The HPLC-HRMS and MS/MS data generated during this study have been deposited at MassIVE (gnps.ucsd.edu) under accession code MSV000088750 (doi:10.25345/C55W01).
