# Supplementary material for: Parallel pathways for serotonin biosynthesis and metabolism in C. elegans
Source: Nat Chem Biol. Author manuscript; Available in PMC 2024 Feb 1. (PMC9898190; doi:10.1038/s41589-022-01148-7)
Supplement: Supplementary Information [file NIHMS1859020-supplement-Supplementary_Information.pdf]

Parallel pathways for serotonin biosynthesis and metabolism in *C. elegans*

Supplementary Information

Jingfang Yu[1], Merly C. Vogt[2], Bennett W. Fox[1], Chester J. J. Wrobel[1], Diana Fajardo Palomino[1], Brian J. Curtis[1], Bingsen Zhang[1], Henry H. Le[1], Arnaud Tauffenberger[1], Oliver Hobert[2,*], and Frank C. Schroeder[1,*]

[1]Boyce Thompson Institute and Department of Chemistry and Chemical Biology, Cornell University, Ithaca, NY 14853, USA.

[2]Department of Biological Sciences, Columbia University, Howard Hughes Medical Institute, NY 10027, USA.

*Corresponding authors: Frank Schroeder, fs31@cornell.edu, and Oliver Hobert, or38@columbia.edu

Table of Contents **Page**

1. Supplementary Figures S2
3. Supplementary Tables S9
2. Chemical Syntheses S18
4. NMR Spectra S31
5. Supplementary References S55

2. Supplementary Figures

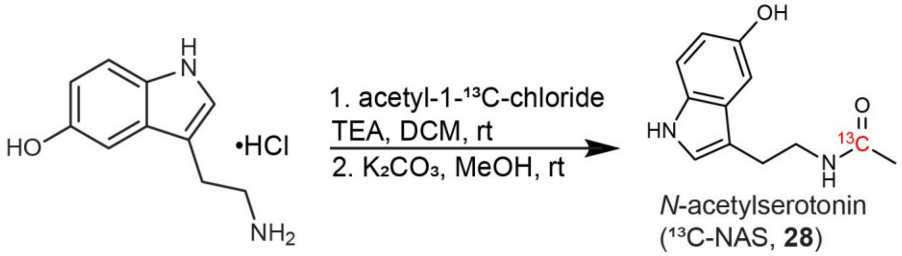

Supplementary Fig. 1: Synthesis of ^{13}C-labeled NAS.

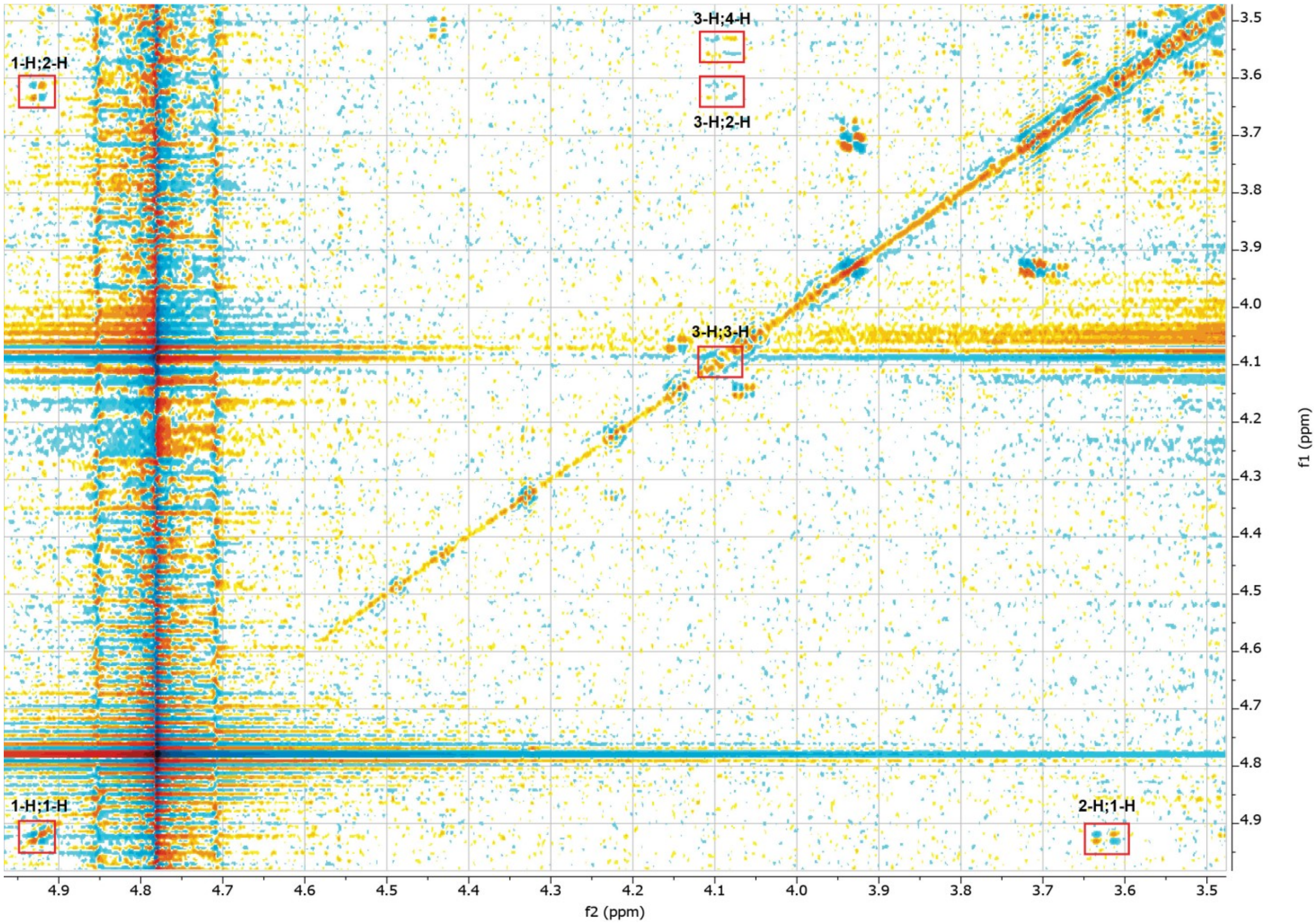

Supplementary Fig. 2: dqfCOSY NMR spectrum (CD_3OD, Bruker AVANCE III HD, 800 MHz) of isolated sngl#2 (**30**) from WT animals treated with 0.5 mM serotonin. Crosspeaks showed sngl#2 is a β-glucoside with 3-*O*-phosphorylation modification.

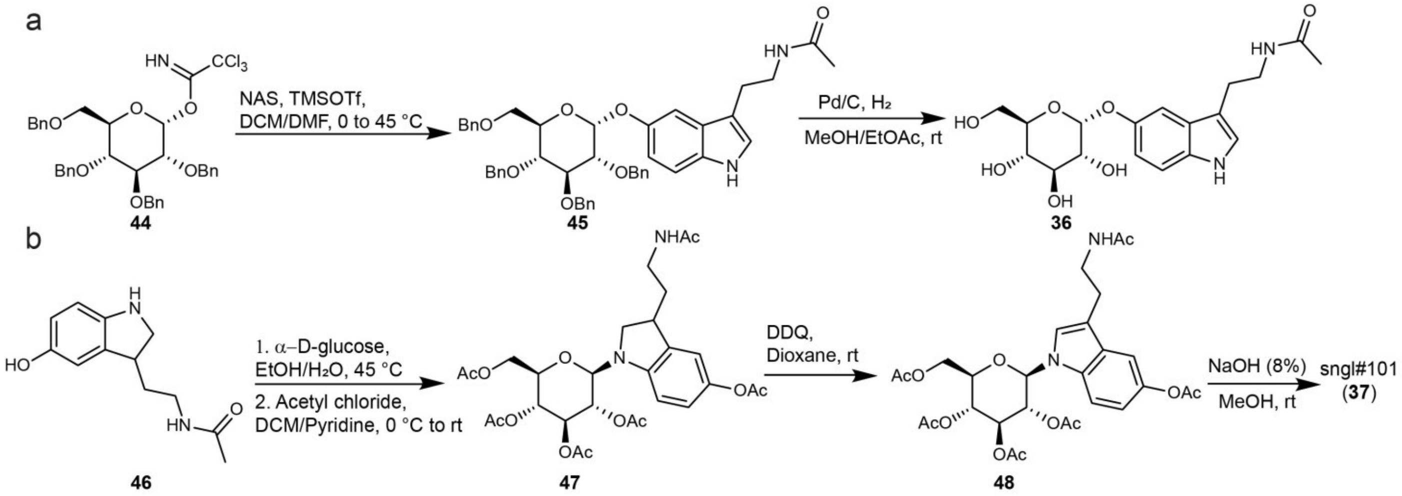

Supplementary Fig. 3: a, Scheme for synthesis of **36** (α-*O*-linked isomer of sngl#1). **b**, Scheme for synthesis of β-*N*-linked serotonin glucoside sngl#101.

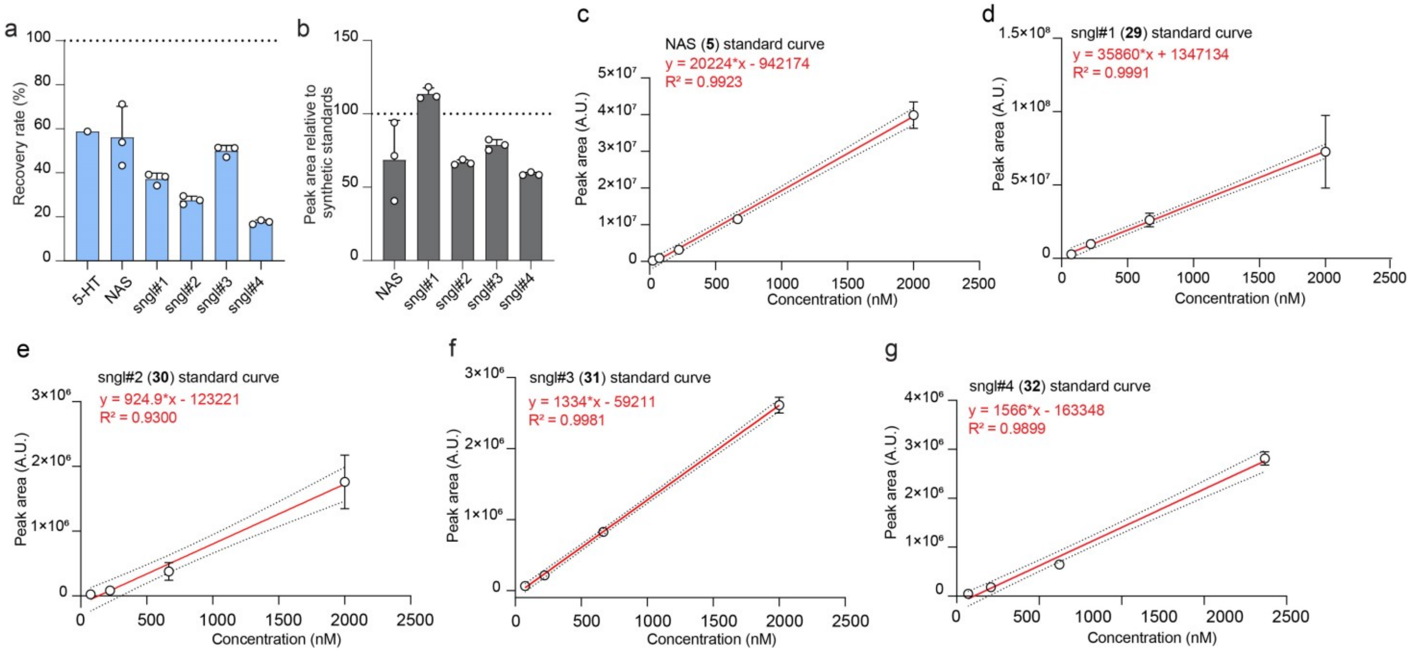

Supplementary Fig. 4: a, Recovery rate of 5-HT, NAS, and sngl#1-4. Shown are relative abundances of 5-HT, NAS, and sngl#1-4 added to *pah-1(syb3596);tph-1(mg280)* mutant worms at the time of harvest compared to *pah-1(syb3596);tph-1(mg280)* mutant *endo*-metabolome samples to which compounds were added right before mass spectrometric analysis (in case of 5-HT, prior to derivatization, see Methods). **b**, Effect of ion suppression on quantification of NAS and sngl#1-4 in worm *endo*-metabolome samples. Shown are measured peak areas for NAS and sngl#1-4 obtained for samples of *pah-1(syb3596);tph-1(mg280)* mutant *endo*-metabolome, to which NAS and sngl#1-4 were added at a concentration of 1 µM right before mass spectrometric analysis, relative to peak areas obtained for a pure sample containing NAS and sngl#1-4 at 1 µM. **c-g**, Standard curve for MS-based detection of NAS (**5**), sngl#1 (**29**), sngl#2 (**30**), sngl#3 (**31**), and sngl#4 (**32**). Data in **a** and **b** represent 3 independent replicates, except for 5-HT recovery rate in **a**, where n = 1. Data in **c-g** represent 2 independent experiments, except for **e**, where 3 independent experiments were measured for sngl#2 standard curve. Bars in **a-g** indicate mean ± s.d.

a

>Coding sequence

```
ATGCCACCAGCTGGACAAGATGATCTTGACTTCTTGAAGTACGCCATGGAATCGTACGTTGCTGACGTCAACG
CCGACATTGGCAAGACTACTATCGTATTCACTCTTCGTGAAAAGGCAGGAGCTCTCGCTGAAACATTGAAGCT
GTTCCAGGCACATGATGTGAATCTGTCTCACATTGAATCAAGACCATCAAAGACTCATGAAGGATGCTATGAG
GTGCTCGTTGAATTTGCTGAAGCTGAAGACCATCGTAAGATTGAAGGAGTTATTGAGCATTTCCAACAAAAAG
CTGAAAAGAAGGTTCTTGTTCAAGACTGGAACACCAAAAACAAACAAAACAAGGATTCTGTTCCATGGTTCCC
ACAGAAGATCAACGACATCGATCAATTTGCCAACAGAATTCTTTCTTATGGAGCTGAGCTTGATGCCGATCAC
CCTGGATTTAAGGACATGACCTACCGCGAGCGCAGAAAGTTTTTCGCCGATATTGCATTCAACTTCAAACACG
GAGACAAGATCCCTACTATCACCTACACTGATGAAGAAATTGCCACGTGGCGTACAGTCTACAACGAGCTGAC
AGTTATGTACCCGAAAAACGCTTGCCAAGAGTTCAACTACATATTCCCACTCCTCCAGCAGAATTGTGGTTTT
GGACCTGACCGCATTCCACAATTGCAGGATGTTTCAGATTTTTTGAAGGATTGTACCGGGTACACGATTCGAC
CAGTCGCTGGTCTTCTTTCTTCTCGTGATTTCTTGGCTGGTTTGGCCTTCCGTGTTTTTCATTCCACACAATA
CATTCGCCATCATTCCGCTCCAAAATACACACCTGAACCAGATATCTGCCACGAGCTTCTGGGACATGTTCCA
CTATTTGCTGATGTTGAATTTGCACAATTCTCACAGGAAATCGGTCTTGCTTCTCTTGGAGCTCCAGATGATG
TTATTGAAAAACTTGCCACACTCTACTGGTTCACAATCGAATTTGGAATCTGTCAACAAGATGGGGAGAAAAA
AGCTTACGGAGCCGGACTTTTGAGTTCATTTGGAGAGCTTCAATATGCGTTGAGTGATAAGCCGGAAGTTGTA
GATTTTGATCCAGCTGTATGTTGTGTCACCAAATATCCAATCACAGAATATCAGCCAAAGTATTTCTTAGCTG
AATCATTTGCAAGTGCTAAGAACAAACTTAAATCATGGGCAGCTACCATCAATCGTCCATTCCAAATTCGTTA
TAATGCTTACACTCAACGAGTTGAAATTCTCGACAAGGTAGCAGCACTTCAACGTCTCGCAAGAGACATCAGA
AGTGATATTTCTACTTTGGAAGAAGCTCTTGGAAAAGTGAACAATCTCAAGATGAAGTGA
```

b

```
H. sapiens   23  SYIEDNCNQNGAISLIFSLKEEVGALAKVLRLFEENDVNLTHIESRPSRLKKDEYEFFTH  82
                 SY+ D     G  +++F+L+E+ GALA+ L+LF+ +DVNL+HIESRPS+  +  YE
C. elegans   18  SYVADVNADIGKTTIVFTLREKAGALAETLKLFQAHDVNLSHIESRPSKTHEGCYEVLVE  77

             83  L----DKRSLPALTNII------KILRHDIGATVHELSRDKKKDTVPWFPRTIQELDRFA  132
                      D R +  +          K+L  D           + KD+VPWFP+ I ++D+FA
             78  FAEAEDHRKIEGVIEHFQQKAEKKVLVQDWNT-----KNKQNKDSVPWFPQKINDIDQFA  132

            133  NQILSYGAELDADHPGFKDPVYRARRKQFADIAYNYRHGQPIPRVEYMEEEKKTWGTVFK  192
                 N+ILSYGAELDADHPGFKD  YR RRK FADIA+N++HG  IP + Y +EE  TW TV+
            133  NRILSYGAELDADHPGFKDMTYRERRKFFADIAFNFKHGDKIPTITYTDEEIATWRTVYN  192

            193  TLKSLYKTHACYEYNHIFPLLEKYCGFHEDNIPQLEDVSQFLQTCTGFRLRPVAGLLSSR  252
                  L  +Y  +AC E+N+IFPLL++ CGF  D IPQL+DVS FL+ CTG+ +RPVAGLLSSR
            193  ELTVMYPKNACQEFNYIFPLLQQNCGFGPDRIPQLQDVSDFLKDCTGYTIRPVAGLLSSR  252

            253  DFLGGLAFRVFHCTQYIRHGSKPMYTPEPDICHELLGHVPLFSDRSFAQFSQEIGLASLG  312
                 DFL GLAFRVFH TQYIRH S P YTPEPDICHELLGHVPLF+D  FAQFSQEIGLASLG
            253  DFLAGLAFRVFHSTQYIRHHSAPKYTPEPDICHELLGHVPLFADVEFAQFSQEIGLASLG  312

            313  APDEYIEKLATIYWFTVEFGLCKQGDSIKAYGAGLLSSFGELQYCLSEKPKLLPLELEKT  372
                 APD+ IEKLAT+YWFT+EFG+C+Q    KAYGAGLLSSFGELQY LS+KP+++  +
            313  APDDVIEKLATLYWFTIEFGICQQDGEKKAYGAGLLSSFGELQYALSDKPEVVDFDPAVC  372

            373  AIQNYTVTEFQPLYYVAESFNDAKEKVRNFAATIPRPFSVRYDPYTQRIEVLDNTQQLKI  432
                  +  Y +TE+QP Y++AESF  AK K++++AATI RPF +RY+ YTQR+E+LD    L+
            373  CVTKYPITEYQPKYFLAESFASAKNKLKSWAATINRPFQIRYNAYTQRVEILDKVAALQR  432

            433  LADSINSEIGILCSALQKI  451
                 LA  I S+I  L  AL K+
            433  LARDIRSDISTLEEALGKV  451
```

CRISPR deletion

Supplementary Fig. 5: a, Coding sequence of *pah-1 (syb3601)*. Sections marked red were deleted. **b,** Sequence alignments of *C. elegans* PAH-1 and human PAH. The protein sequence of Cel-PAH-1 was submitted to an NCBI BLASTp search (restricted to species *Homo sapiens*, conditional compositional BLOSUM62, gap open cost: 11, gap extension cost: 1, word size: 6).

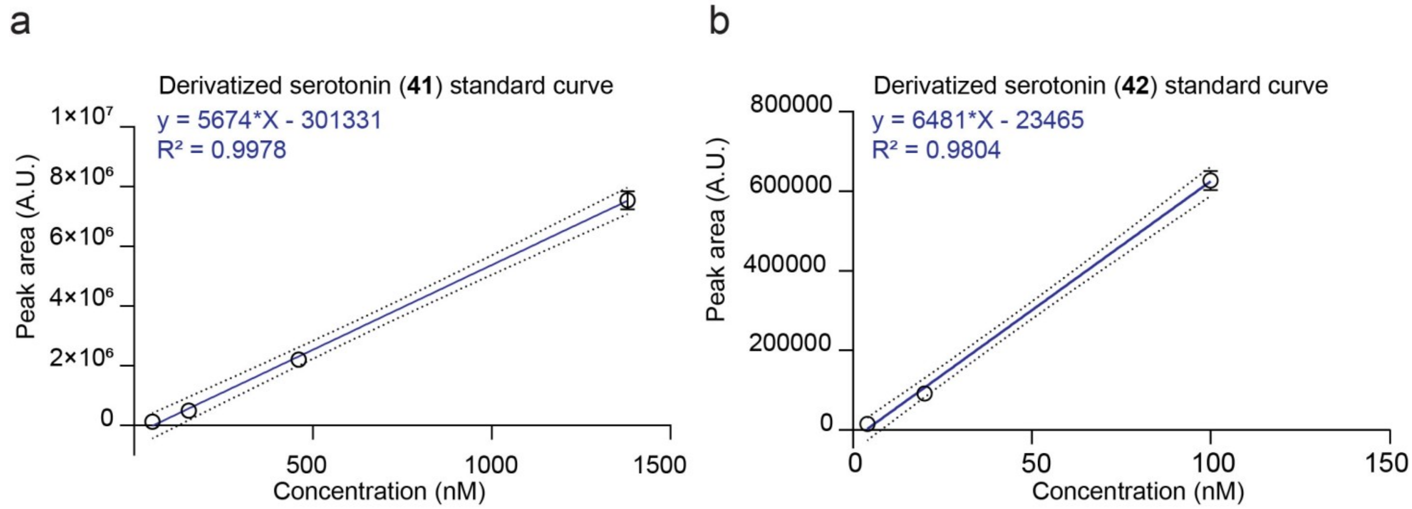

Supplementary Fig. 6: a, **b**, Standard curve for MS-based detection of mono-dansyl-derivatized serotonin (**41**) and di-dansyl-derivatized serotonin (**42**). Data in **a** and **b** represent 2 independent experiments, and bars indicate mean ± s.d.

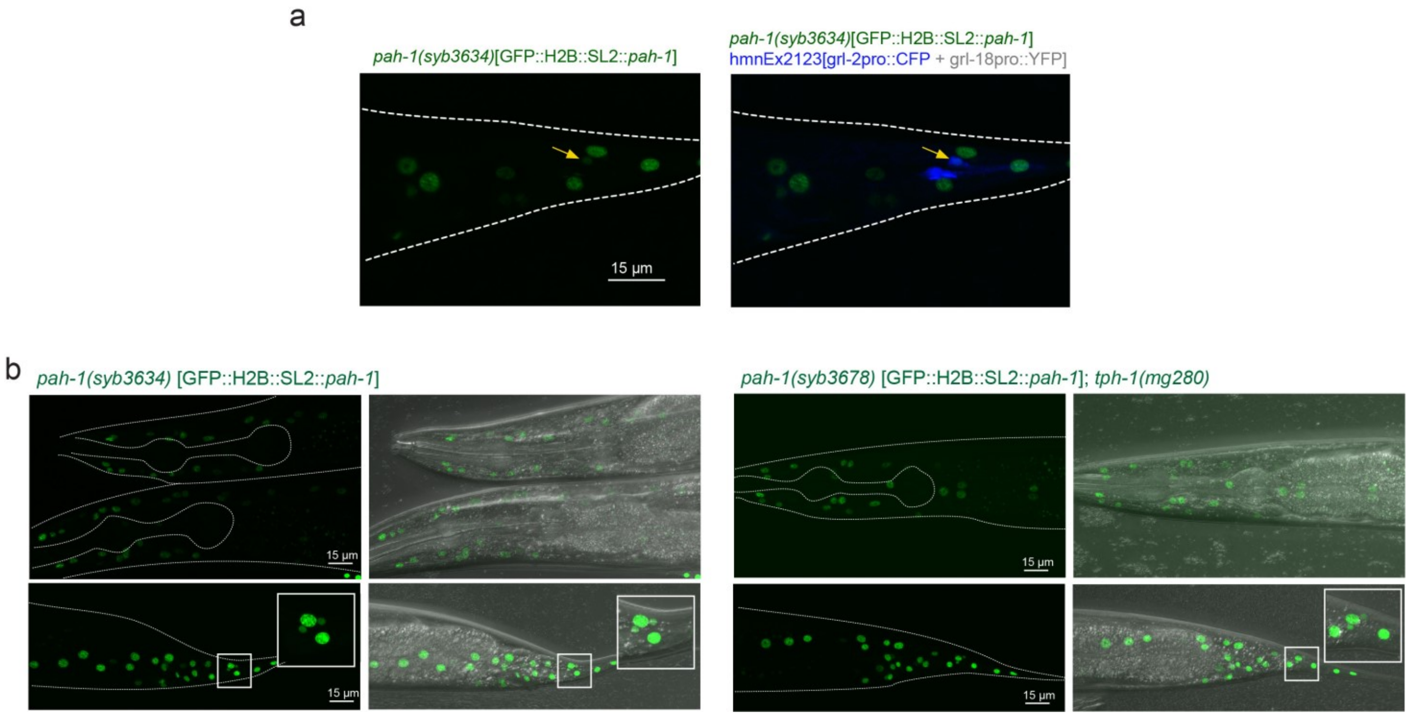

Supplementary Fig. 7: **a**, *GFP::H2B::SL2::PAH-1* (green) co-localizes with the phasmid socket glial cell-specific marker *grl-2pro::CFP* (blue) in the tail of adult hermaphrodites. **b**, Comparison of *GFP::H2B::T2A::pah-1*-expression in WT and *tph-1(mg280)* mutant background revealed no differences, indicating that loss of *tph-1* does not strongly affect *pah-1* expression patterns. Scale bar = 15 µm. For each genotype, at least 10 animals were scored on day 1 of Adulthood for GFP expression under well fed conditions on at least 2 different days.

2. Supplementary Tables

Supplementary Table 1. ***C. elegans*** **strains used in this work.**

Gene/genotype	Strain	Allele	Source	Genotyped	Outcrossed
C. elegans wildtype	N2		Caenorhabditis Genetics Center (CGC)	Yes	
tph-1	MT15434	*mg280*	CGC	Yes	No
tph-1	MT14984	*n4622*	CGC	Yes	Yes
pah-1	PHX3601	*syb3601*	SunyBiotech	Yes	No
pah-1(syb3596);tph-1(mg280)	PHX3596		SunyBiotech	Yes	No
bas-1	MT7988	*ad446*	CGC	Yes	Yes
cat-2	CB1112	*e1112*	CGC	Yes	No
glo-1	DH10	*zu437*	CGC	Yes	Yes
pah-1(syb3634)[GFP::H2B::T2A::pah-1]	PHX3634		SunyBiotech	Yes	No
pah-1(syb3678)[GFP::H2B::T2A::pah-1];tph-1(mg280)	PHX3678		SunyBiotech	Yes	No
pah-1(syb3634)[GFP::H2B::T2A::pah-1];nsIs698 [mir-228p::NLS::RFP]	OH17653		This work	Yes	No
pah-1(syb3634)[GFP::H2B::T2A::pah-1];nsIs698[mir-228p::NLS::RFP];tph-1[mg280]	OH17654		This work	Yes	No
pah-1(syb3634)[GFP::H2B::T2A::pah-1], hmnEx2123[grl-2pro::CFP + grl-18pro::YFP]	OH17655		This work	Yes	No
cest-4(syb5218)[cest-4::SL2::GFP::H2B]	PHX5218	*syb5218*	SunyBiotech	Yes	No
bas-1(syb5923)[bas-1::SL2::GFP::H2B]	PHX5923	*syb5923*	SunyBiotech	Yes	No

cest-1.1	PS8031	*sy1180*	Le. *et al*[1]	Yes	No
cest-1.1	PS8032	*sy1181*	Le. *et al*[1]	Yes	No
cest-1.2	PHX3928	*syb3928*	Wrobel. *et al*[2]	Yes	No
cest-19	PS8029	*sy1178*	Le. *et al*[1]	Yes	No
cest-19	PS8030	*sy1179*	Le. *et al*[1]	Yes	No
cest-2.2	PS8008	*sy1170*	Le. *et al*[1]	Yes	No
cest-2.2	PS8009	*sy1171*	Le. *et al*[1]	Yes	No
cest-3	PHX3937	*syb3937*	SunyBiotech	Yes	No
cest-33	PS8033	*sy1182*	Le. *et al*[1]	Yes	No
cest-33	PS8034	*sy1183*	Le. *et al*[1]	Yes	No
cest-4	PS8116	*sy1192*	Le. *et al*[1]	Yes	No
cest-4	PS8117	*sy1193*	Le. *et al*[1]	Yes	No
cest-6	RB1804	*ok2338*	Le. *et al*[1]	Yes	No
cest-8	PS7953	*sy1163*	Sternberg lab	Yes	No
cest-8	PS7954	*sy1164*	Sternberg lab	Yes	No
ges-1	RB2053	*ok2716*	Le. *et al*[1]	Yes	No

Supplementary Table 2. *E. coli* strains used in this work.

Strain	Source
OP50	Caenorhabditis Genetics Center (CGC)
BW25113 (K12)	Baba. *et al*[3]
JW3686-7 (*ΔtnaA*)	Baba. *et al*[3]

Supplementary Table 3. Primer sequence for RT-PCR.

Primer	Sequence
ama-1 FWD	CGA CGA GTC CAA CGT ACT CTC CA
ama-1 REV	AAT AGG TAG GCG ACG ACG GC
cat-2 FWD	CAG CTC ACC GAT CAG ATG AGG
cat-2 REV	GCG TTG GGT CAG TGG AAG
tph-1 FWD	CAA TAC TAC TCG AAG AAA GCT GCT
tph-1 REV	TCC TAA CGA TCC ACT TCG ACG
pah-1 FWD	TGA TGC CGA TCA CCC TGG
pah-1 REV	GTG TAG CTG ATA GTA GGG ATC TTG TC

Supplementary Table 4. NMR spectroscopic data for **36**. ^{1}H (600 MHz), dqfCOSY, HSQC and HMBC NMR spectroscopic data were acquired in methanol-d_4.

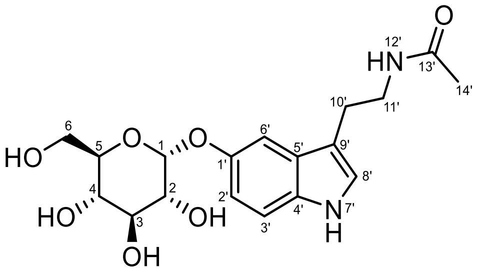

36

**Position**	**δ ^{13}C [ppm]**	**δ ^{1}H [ppm] (Multiplicity, J_{HH}[Hz])**	**HMBC**
1	100.7	5.40 (d, $J_{1,2}$ = 3.7)	C-1', C-2, C-3
2	73.3	3.57 (dd, $J_{2,3}$ = 9.7)	C-3, C-4 (weak)
3	74.9	3.89 (dd, $J_{3,4}$ = 9.2)	C-2, C-4
4	71.5	3.43 (m)	C-3, C-5, C-6
5	73.8	3.86	C-6 (weak)
6a	62.3	3.73 (dd, $J_{5,6a}$ = 5.4, $J_{6a,6b}$ = 12.0)	C-4, C-5
6b		3.83 (dd, $J_{5,6b}$ = 2.4)	C-4, C-5
1'	151.9		
2'	114.5	6.97 (dd, $J_{2',3'}$ = 8.7, $J_{2',6'}$ = 2.3)	C-1', C-4', C-6'
3'	112.2	7.23 (d)	C-1', C-5', C-6'
4'	134.2		
5'	128.8		
6'	107.2	7.40 (d)	C-1', C-2', C-4', C-9'
7'			
8'	124.1	7.05 (s)	C-1'(weak), C-4', C-5', C-6' (weak), C-9', C-10', C-11'
9'	113.0		
10'	25.9	2.89 (t, $J_{10',11'}$ = 7.4)	C-5', C-8', C-9', C-11'
11'	41.2	3.44 (m)	C-9', C-10', C-13'
12'			
13'	172.8		
14'	22.3	1.91 (s)	C-13'

Supplementary Table 5. NMR spectroscopic data for sngl#101 (**37**). ^{1}H (600 MHz), dqfCOSY, HSQC and HMBC NMR spectra were acquired in methanol-d_4.

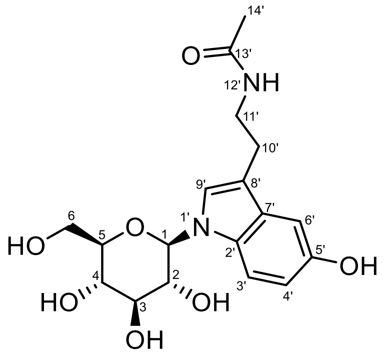

sngl#101 (**37**)

**Position**	**δ ^{13}C [ppm]**	**δ ^{1}H [ppm] (Multiplicity, J_{HH}[Hz])**	**HMBC**
1	86.4	4.85 (d, $J_{1,2}$ = 9.0)	C-2, C-3, C-5, C-7’, C-9’
2	73.1	3.89 (t, $J_{2,3}$ = 9.0)	C-1, C-3
3	78.7	3.57 (t, $J_{3,4}$ = 9.0)	C-2, C-4
4	71.1	3.46 (t)	C-3, C-5, C-6 (weak)
5	80.0	3.54 (ddd, $J_{4,5}$ = 9.7, $J_{5,6a}$ = 5.6, $J_{5,6b}$ = 2.3)	C-2, C-4
6a	62.3	3.69 (dd, $J_{6a,6b}$ = 12.2)	C-1 (weak)
6b		3.86 (dd)	C-4 (weak)
1’			
2′	130.5		
3′	111.7	7.32 (d, $J_{3',4'}$ = 8.8)	C-2’, C-5’, C-6’ (weak), C-7’ (weak)
4′	112.3	6.72 (dd, $J_{4',6'}$ = 2.3)	C-5’ (weak), C-6’, C-7’
5′	151.7		
6′	103.6	6.92 (d)	C-4’, C-5’, C-7’, C-8’ (weak)
7′	132.8		
8′	113.3		
9′	124.6	7.19 (s)	C-2’, C-3’, C-7’, C-8’ (weak)
10′	25.8	2.84 (t, $J_{10',11'}$ = 7.3)	C-2’, C-8’, C-9’, C-11’
11’	40.9	3.44 (t)	C-2’, C-8’, C-9’, C-10’, C-13’
12’			
13’	172.9		
14’	22.1	1.91 (s)	C-13’

Supplementary Table 6. NMR spectroscopic data for sngl#1 (**29**). ^{1}H (600 MHz), dqfCOSY, HSQC and HMBC NMR spectra were acquired in methanol-d_4.

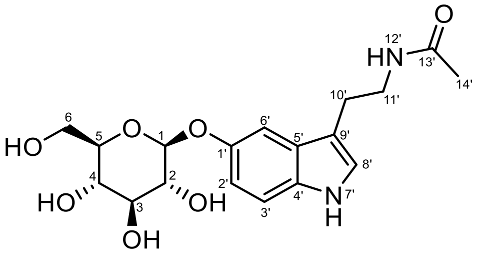

sngl#1 (**29**)

Position	δ ^{13}C [ppm]	δ ^{1}H [ppm] (Multiplicity, J_{HH}[Hz])	HMBC
1	103.9	4.85 (m)	C-1', C-2, C-3
2	77.7	3.46 (m)	C-1', C-3
3	71.3	3.39 (m)	C-4, C-6 (weak)
4	74.8	3.47 (m)	C-2, C-5
5	77.7	3.44 (m)	C-3, C-4
6a	62.3	3.71 (dd, $J_{5,6a}$ = 5.9, $J_{6a,6b}$ = 12.0)	C-3, C-5
6b		3.93 (dd, $J_{5,6b}$ = 2.2)	C-3, C-5 (weak)
1'	152.4		
2′	114.0	6.96 (dd, $J_{2',3'}$ = 8.8, $J_{2',6'}$ = 2.3)	C-1', C-4', C-6'
3′	112.1	7.22 (d)	C-1', C-4' (weak), C-5', C-6'
4′	134.0		
5′	128.6		
6′	106.3	7.35 (d)	C-1', C-2', C-3' (weak), C-4', C-9'
7′			
8′	124.1	7.06 (s)	C-1' (weak), C-4', C-5', C-6' (weak), C-9', C-11'
9′	112.8		
10′	25.6	2.89 (t, $J_{10',11'}$ = 7.4)	C-5', C-8', C-9', C-11'
11'	40.9	3.45 (m)	C-10', C-13'
12'			
13'	172.8		
14'	22.2	1.91 (s)	C-13'

Supplementary Table 7. NMR spectroscopic data for sngl#2 (**30**). ^{1}H (600 MHz), dqfCOSY, HSQC and HMBC NMR spectra were acquired in methanol-d_4.

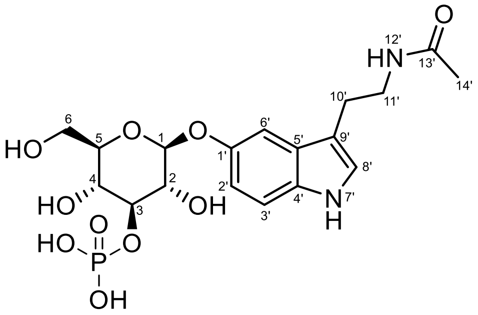

sngl#2 (**30**)

**Position**	**δ ^{13}C [ppm]**	**δ ^{1}H [ppm] (Multiplicity, J_{HH}[Hz])**	**HMBC**
1	103.5	4.93 (d, $J_{1,2}$ = 7.3)	C-1' (weak)
2	74.0	3.65 (br, m)	
3	83.5	4.20 (br, m)	
4	70.5	3.60 (br, m)	
5	77.3	3.49 (br, m)	
6a	62.2	3.73 (dd, $J_{5,6a}$ = 5.1, $J_{6a,6b}$ = 12.1)	
6b		3.93 (d)	
1'	152.4		
2'	114.0	6.95 (dd, $J_{2',3'}$ = 8.7, $J_{2',6'}$ = 1.8)	C-1', C-4', C-6'
3'	112.2	7.22 (d)	C-1', C-5', C-6'
4'	134.1		
5'	128.7		
6'	106.5	7.35 (d)	C-1', C-2', C-4', C-9'
7'			
8'	124.3	7.06 (s)	C-1' (weak), C-3', C-4', C-5', C-6', C-9', C-10'
9'	113.0		
10'	25.9	2.89 (t, $J_{10',11'}$ = 7.4)	C-5', C-8', C-9', C-11'
11'	41.1	3.44 (t)	C-9', C-10', C-13'
12'			
13'	172.9		
14'	22.3	1.91 (s)	C-13'

Supplementary Table 8. NMR spectroscopic data for sngl#3 (**31**). ^{1}H (800 MHz), dqfCOSY, HSQC and HMBC NMR spectra were acquired in methanol-d_4.

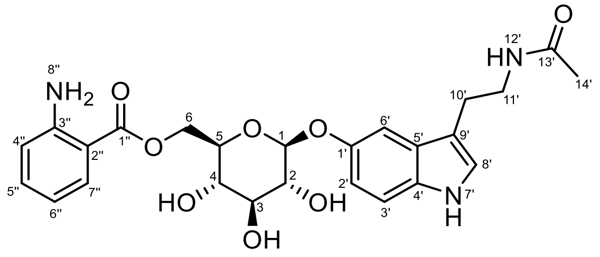

sngl#3 (**31**)

Position	δ ^{13}C [ppm]	δ ^{1}H [ppm] (Multiplicity, J_{HH}[Hz])	HMBC
1	104.0	4.88 (d, $J_{1,2}$ = 7.3)	C-3, C-4, C-5, C-1’
2	74.9	3.50 (m)	C-1, C-3
3	77.8	3.52 (m)	C-2, C-4
4	71.9	3.46 (m)	C-3, C-5, C-6
5	75.3	3.74 (ddd, $J_{5,6a}$ = 2.1)	C-1, C-3, C-4, C-6
6a	64.4	4.70 (dd, $J_{6a,6a}$ = 11.8)	C-4, C-5, C-1’’
6b		4.37 (dd, $J_{5,6b}$ =7.0)	C-4, C-5, C-1’’
1’	152.6		
2′	114.2	6.98 (dd, $J_{2',3'}$ = 8.7, $J_{2',6'}$ = 2.2)	C-1’, C-4’, C-6’
3′	112.2	7.16 (d)	C-1’, C-5’, C-6’
4′	134.6		
5′	129.0		
6′	107.0	7.28 (d)	C-1’, C-2’, C-4’, C-9’
7′			
8′	124.2	7.05 (s)	C-1’ (weak), C-4’, C-5’, C-9’, C-10’ (weak), C-11’ (weak)
9′	113.3		
10′	25.9	2.81 (t, $J_{10',11'}$ = 7.3)	C-5’, C-8’, C-9’, C-11’
11’	41.1	3.39 (t)	C-9’, C-10’, C-13’
12’			
13’	173.1		
14’	22.3	1.89 (s)	C-13’
1’’	169.2		
2’’	111.2		
3’’	152.6		
4’’	117.5	6.71 (d, $J_{4'',5''}$ = 8.3)	C-1’’ (weak), C-2’’, C-6’’, C-7’’ (weak)
5’’	134.8	7.22 (ddd, $J_{5'',7''}$ = 1.3, $J_{5'',6''}$ = 6.7)	C-3’’, C-7’’
6’’	116.4	6.49 (ddd, $J_{4'',6''}$ = 0.9, $J_{6'',7''}$ = 8.1)	C-1’’ (weak), C-2’’, C-3’’, C-4’’, C-5’’, C-7’’
7’’	132.0	7.78 (dd)	C-1’’, C-3’’, C-4’’ (weak), C-5’’
8’’			C-1’’, C-3’’, C-4’’ (weak), C-5’’

Supplementary Table 9. NMR spectroscopic data for sngl#4 (**32**). ^{1}H (600 MHz), dqfCOSY, HSQC and HMBC NMR spectra were acquired in methanol-d_4. Spectra chemical shifts are relative to residual methanol-d_4.

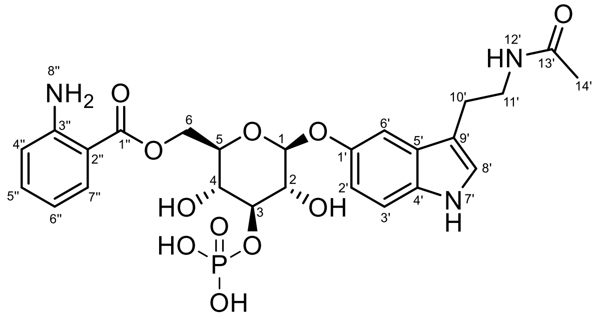

sngl#4 (**32**)

Position	δ ^{13}C [ppm]	δ ^{1}H [ppm] (Multiplicity, J_{HH}[Hz])	HMBC
1	103.3	4.97 (d, $J_{1,2}$ = 7.8)	C-1'
2	73.9	3.70 (t, $J_{2,3}$ = 8.0)	C-1, C-3
3	83.3	4.26 (m)	
4	70.8	3.69 (m)	C-3, C-5, C-6
5	74.6	3.81 (m)	C-5, C-1''
6a	64.0	4.39 (dd, $J_{5,6a}$ =7.0, $J_{6a,6b}$ = 11.6)	C-5 (weak), C-1''
6b		4.70 (dd, $J_{5,6b}$ =1.1)	C-1''(weak)
1'	151.6		
2'	114.2	6.96 (dd, $J_{2',3'}$ = 8.8, $J_{2',6'}$ = 2.0)	C-1', C-4', C-6'
3'	112.4	7.16 (d)	C-1', C-5', C-6'(weak)
4'	134.1		
5'	128.5		
6'	106.9	7.28 (d)	C-1', C-2'(weak), C-4', C-9'
7'			
8'	124.1	7.04 (s)	C-1' (weak), C-4', C-5', C-6'(weak), C-9'
9'	112.9		
10'	25.7	2.78 (t, $J_{10',11'}$ = 7.4)	C-5', C-8', C-9', C-11'
11'	40.9	3.37 (t)	C-9', C-10', C-13'
12'			
13'	172.7		
14'	22.3	1.89 (s)	C-13'
1''	168.3		
2''	112.0		
3''	150.4		
4''	117.6	6.79 (d, $J_{4'',5''}$ = 8.1)	C-2'', C-6''
5''	134.8	7.26 (m)	C-3'', C-7''
6''	116.4	6.58 (t, $J_{5'',6''}$ = 7.6)	C-2'', C-4'', C-7''(weak)
7''	131.9	7.79 (dd, $J_{5'',7''}$ = 1.0, $J_{6'',7''}$ = 7.9)	C-1'', C-3'', C-5''
8''			

3. Chemical Syntheses

3.1. Reagents and General Procedures

All oxygen and moisture-sensitive reactions were carried out under argon atmosphere in flame-dried glassware. Solutions and solvents sensitive to moisture and oxygen were transferred via standard syringe and cannula techniques. All commercial reagents were purchased as reagent grade and, unless otherwise stated, were purchased from Sigma-Aldrich and used without any further purification. Acetic acid (AcOH), acetonitrile (ACN), dichloromethane (DCM), ethyl acetate (EtOAc), *N*,*N*-dimethylformamide (DMF), tetrahydrofuran (THF), formic acid, hexanes and methanol (MeOH) used for chromatography and as a reagent or solvent were purchased from Thermo Fisher Scientific. Acetyl chloride (1-^{13}C, 99%) was purchased from Cambridge Isotope Laboratories, *N*-acetylserotonin (NAS) was obtained from Biosynth International, Boc-2-aminobenzoic acid (Boc-2-Abz-OH) was from Chem-Impex International, and trifluoroacetic acid (TFA) was from Tokyo Chemical Industry, fluoxetine hydrochloride was from Spectrum Chemical. Dichloromethane (DCM), and *N*,*N*-dimethylformamide (DMF) were dried with 3 Å molecular sieves prior to use. Thin-layer chromatography (TLC) was performed using J. T. Baker Silica Gel IB2F plates. Flash chromatography was performed using Teledyne Isco CombiFlash systems and Teledyne Isco RediSep Rf silica and C18 reverse phase columns. All deuterated solvents were purchased from Cambridge Isotopes. Abbreviations used: triethylamine (TEA), 2,3-dichloro-5,6-dicyano-1,4-benzoquinone (DDQ), trichloroacetonitrile (CCl_3CN), 1,8-diazabicyclo[5.4.0]undec-7-ene (DBU), trifluoromethanesulfonate (TMSOTf), *N*-ethyl-*N'*-(3-dimethylaminopropyl)carbodiimide hydrochloride (EDC·HCl), 4-dimethylaminopyridine (DMAP), 1,3-dichloro-1,1,3,3-tetraisopropyldisiloxane ($TIPDSiCl_2$), 3-chloroperoxybenzoic acid (*m*-CPBA). Nuclear Magnetic Resonance (NMR) spectra were recorded on Bruker INOVA 500 (500 MHz) and Varian INOVA 600 (600 MHz) spectrometers at Cornell University's NMR facility and Bruker AVANCE III HD 800 MHz (800 MHz) or Bruker AVANCE III HD 600 MHz (600 MHz) at SUNY ESF's NMR facility. ^{1}H NMR chemical shifts are reported in ppm (δ) relative to residual solvent peaks (7.26 ppm for chloroform-*d*, 3.31 ppm for methanol-d_4, 2.05 ppm for acetone-d_6). NMR-spectroscopic data are reported as follows: chemical shift, multiplicity (s = singlet, d = doublet, t = triplet, q = quartet, m = multiplet, br = broad), coupling constants (Hz), and integration and often tabulated including 2D NMR data. ^{13}C NMR chemical shifts are reported in ppm (δ) relative to residual solvent peaks (77.16 ppm for chloroform-*d*, 49.00 ppm for methanol-d_4, 29.9 ppm for acetone-d_6). All NMR data processing was done using MestreLab MNOVA version 14.2.1-27684 (https://mestrelab.com/).

3.2. Synthesis of acylated serotonin derivatives

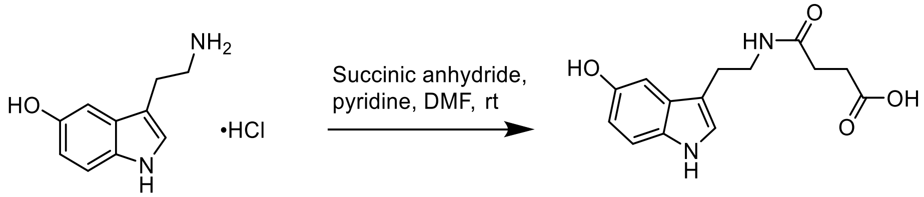

14

4-((2-(5-hydroxy-1*H*-indol-3-yl)ethyl)amino)-4-oxobutanoic acid (14). To a solution of serotonin hydrochloride (128.1 mg, 0.602 mmol, 1.0 eq.) in DMF (6 mL) was added succinic anhydride (78.3 mg, 0.783 mmol, 1.3 eq.) and pyridine (0.6 mL). The mixture was stirred at room temperature for 24 hours and concentrated *in vacuo.* Flash column chromatography on silica using a gradient of 0-50% MeOH in DCM afforded **14** (165.4 mg, 99%) as clear oil.

^{1}H NMR (500 MHz, methanol-*d$_4$*): δ (ppm) 7.16 (d, *J* = 8.6 Hz, 1H), 6.98 (s, 1H), 6.96 (d, *J* = 2.3 Hz, 1H), 6.69 (dd, *J* = 2.3, 8.6 Hz, 1H), 3.42 (t, *J* = 7.2 Hz, 2H), 2.83 (t, *J* = 7.2 Hz, 2H), 2.57 (t, *J* = 7.0 Hz, 2H), 2.43 (t, *J* = 7.0 Hz, 2H).

^{13}C NMR (125 MHz, methanol-*d$_4$*): δ (ppm) 176.3, 174.3, 151.0, 132.9, 129.3, 124.3, 112.7, 112.4, 112.3, 103.5, 41.3, 31.5. 30.2, 26.1.

HRMS (ESI) *m/z* calcd for $C_{14}H_{16}N_2O_4$ $[M – H]^-$ 275.1037, found 275.1043.

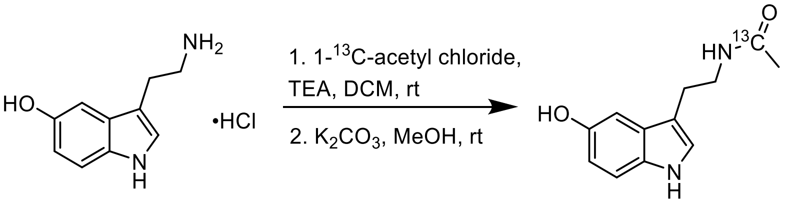

28

***N*-(2-(5-hydroxy-1H-indol-3-yl)ethyl)acetamide-1-^{13}C (28)**. To a suspension of serotonin hydrochloride (132 mg, 0.621 mmol, 1.0 eq.) in DCM (5 mL) was added TEA (433 µL, 3.10 mmol, 5.0 eq.). The stirred mixture was cooled to 0 °C before 1-^{13}*C*-acetyl chloride (93 µL, 1.30 mmol, 2.1 eq.) was added. The mixture was slowly warmed to room temperature and stirred for 24 hours. The reaction mixture was then diluted with DCM, the organics were washed with water, dried with Na_2SO_4, and concentrated *in vacuo*. Crude intermediates were dissolved in MeOH (10 mL), and K_2CO_3 (85.8 mg, 0.621 mmol, 1.0 eq.) was added. The reaction was stirred at room temperature for 2 hours and concentrated to 2 mL *in vacuo*. The residue was diluted with water and extracted with EtOAc twice. The organics were separated, washed with brine, and dried with Na_2SO_4. Flash column chromatography on silica using a gradient of 0-50% MeOH in DCM afforded **28** (98.0 mg, 72%) as light-yellow oil.

^{1}H NMR (600 MHz, methanol-*d$_4$*): δ (ppm) 7.15 (dd, *J* = 0.6, 8.6 Hz, 1H), 6.99 (s, 1H), 6.93 (dd, *J* = 0.6, 2.4 Hz, 1H), 6.66 (dd, *J* = 2.4, 8.6 Hz, 1H), 3.42 (ddd, *J* = 3.7, 7.3, 8.2 Hz, 2H), 2.85 (dt, *J* = 0.6, 7.3 Hz, 2H), 1.91 (d, *J* = 6.1 Hz, 3H). **^{13}C NMR (125 MHz, methanol-*d$_4$*)**: δ (ppm) 175.9

(^{12}C), 173.4(^{13}C), 151.1, 133.1, 129.5, 124.2, 112.6, 112.4, 103.5, 41.4, 26.2, 22.6 (d, *J* = 50.3 Hz).

HRMS (ESI) *m/z* calcd for $C_{11}{}^{13}CH_{14}N_2O_2$ $[M + H]^+$ 220.1161, found 220.1160.

3.3. Synthesis of compound 36

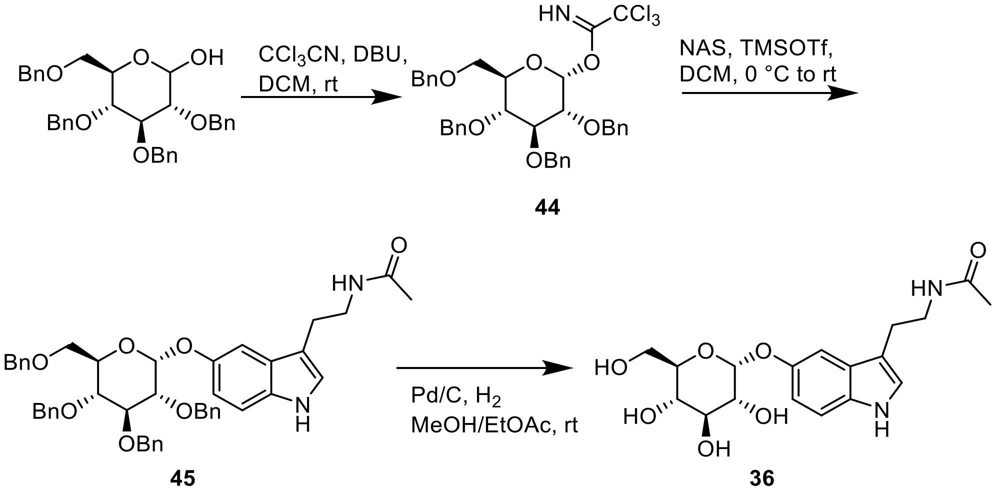

***N*-(2-(5-(((2*R*,3*R*,4*S*,5*R*,6*R*)-3,4,5-tris(benzyloxy)-6-((benzyloxy)methyl)tetrahydro-2*H*-pyran-2-yl)oxy)-1*H*-indol-3-yl)ethyl)acetamide (45).** To a solution of 2,3,4,6-tetra-*O*-benzyl-D-glucopyranose (412 mg, 0.761 mmol, 1.0 eq.) in DCM (2 mL) was added trichloroacetonitrile (152 µL, 1.52 mmol, 2.0 eq.) and DBU (21 µL, 0.152 mmol, 0.2 eq.) under argon. The mixture was stirred at room temperature for 1.5 hours and concentrated *in vacuo*. Flash column chromatography on silica using a gradient of 25% ethyl acetate in hexanes afforded intermediate **44** (502.4 mg, 97%) as clear oil. A well-stirred solution of **44** (502.4 mg, 0.745 mmol, 2.0 eq.) and *N*-acetylserotonin (806 mg, 0.368 mmol, 1.0 eq.) in DCM (4 mL) and DMF (0.8 mL) was cooled to 0 °C, followed by addition of TMSOTf (66 µL, 0.368 mmol, 1.0 eq.), and the solution was allowed to warm to room temperature within 30 minutes. After stirring at 45 °C for 18 hours, the mixture was concentrated *in vacuo*. Flash column chromatography on silica using a gradient of 0-15% MeOH in DCM afforded **45** (59.7 mg, 22%) as clear oil.

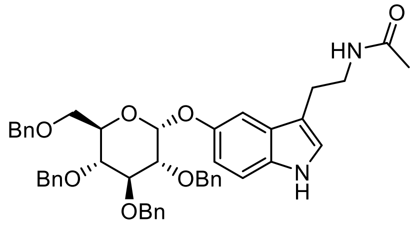

45

^{1}H NMR (500 MHz, chloroform-*d*): δ (ppm) 7.41-7.26 (m, 20H), 7.17-7.14 (m, 2H), 7.04-7.01 (m, 2H), 5.50 (d, *J* = 3.4 Hz, 1H), 5.44 (m, 1H), 5.08 (d, *J* = 10.8 Hz, 1H), 4.90 (d, *J* = 11.0 Hz, 1H), 4.88 (d, *J* = 10.9 Hz, 1H), 4.81 (d, *J* = 12.0 Hz, 1H), 4.72 (d, *J* = 12.0 Hz, 1H), 4.57 (d, *J* = 11.9 Hz, 1H), 4.50 (d, *J* = 10.8 Hz, 1H), 4.41 (d, *J* = 12.0 Hz, 1H), 4.25 (t, *J* = 9.2 Hz, 1H), 4.03 (m, 1H), 3.78-3.71 (m, 3H), 3.62 (dd, *J* = 1.9, 10.8 Hz, 1H), 3.53 (dt, *J* = 6.2, 6.6 Hz, 2H), 2.87 (t, *J* = 6.6 Hz, 2H), 1.90 (s, 3H). **^{13}C NMR (125 MHz, chloroform-*d*)**: δ (ppm) 170.1, 151.2, 139.0, 138.4, 138.2, 138.0, 132.7, 128.62, 128.58, 128.54, 128.48, 128.19, 128.13, 128.05, 128.02,

127.87, 127.82, 127.78, 123.1, 114.3, 113.2, 111.9, 105.8, 96.8, 82.2, 80.0, 77.8, 76.0, 75.3, 75.5, 10.8, 68.7, 39.6, 25.4, 23.5.

***N*-(2-(5-(((2*R*,3*R*,4*S*,5*S*,6*R*)-3,4,5-trihydroxy-6-(hydroxymethyl)tetrahydro-2*H*-pyran-2-yl)oxy)-1*H*-indol-3-yl)ethyl)acetamide (36).** To a solution of **45** (59.2 mg, 0.080 mmol, 1.0 eq.) in a mixture of MeOH and EtOAc (3 mL, v/v = 1:1) was added Pd/C (10% w/w, 38 mg). The stirred reaction mixture was purged with argon for 5 minutes, flushed with hydrogen and then subjected to a hydrogen atmosphere for 2 hours at room temperature, and again purged with argon for 5 minutes. The mixture was filtered through celite and concentrated *in vacuo,* affording **36** as clear oil (29.8 mg, 98%).

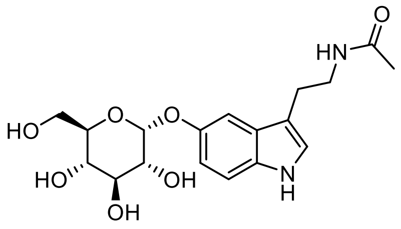

36

See Supplementary Table 4 for NMR spectroscopic data of **36**.

HRMS (ESI) *m/z* calcd for $C_{18}H_{24}N_2O_7$ $[M + Na]^+$ 403.1476, found 403.1486.

3.4. Synthesis of sngl#101 (37)

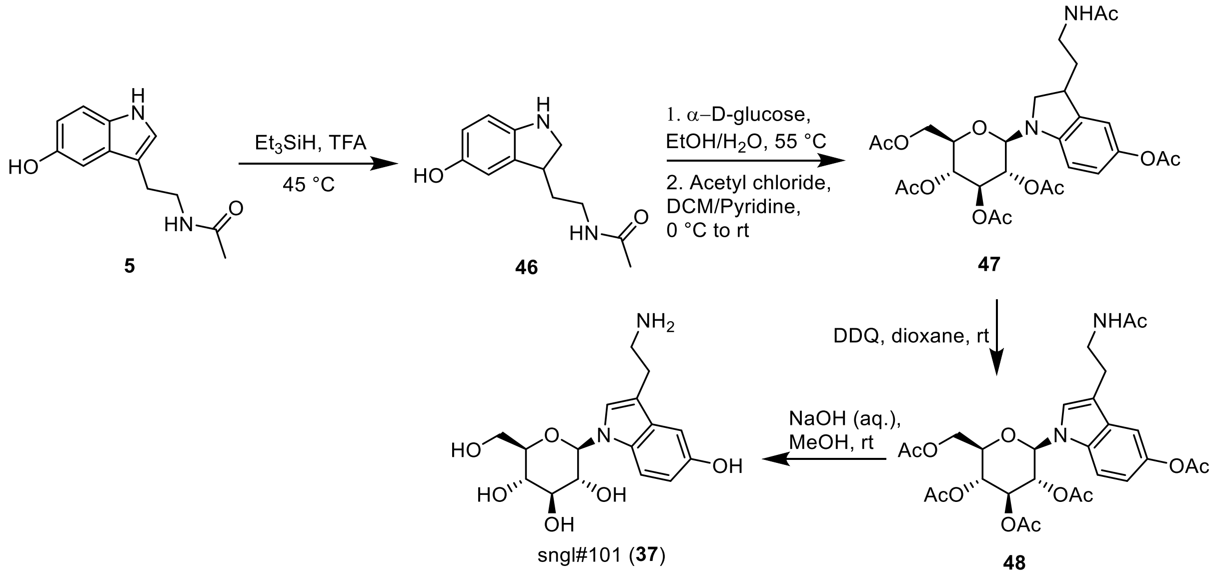

***N*-(2-(5-hydroxyindolin-3-yl)ethyl)acetamide (46)** To a solution of *N*-acetylserotonin (210.2 mg, 0.963 mmol, 1.0 eq.) in TFA (4 mL) was added triethylsilane (185 µL, 1.15 mmol, 1.2 eq.). The mixture was stirred at 45 °C for 4 hours and concentrated *in vacuo*. Flash column chromatography on silica using a gradient of 0-40% MeOH in DCM afforded **46** (209.0 mg, 99%).

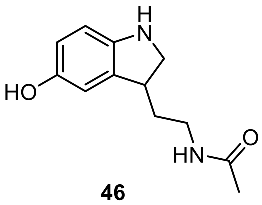

46

^{1}H NMR (500 MHz, methanol-*d$_4$*): δ (ppm) 7.17 (d, *J* = 8.6 Hz, 1H), 6.79 (d, *J* = 2.2 Hz, 1H), 6.74 (dd, *J* = 2.2, 8.6 Hz, 1H), 3.95-3.88 (m, 1H), 3.62-3.55 (m, 1H), 3.49-3.42 (m, 2H), 3.29-3.20 (m, 2H), 2.02-1.94 (m, 1H), 1.88 (s, 3H), 1.72-1.63 (m, 1H). **^{13}C NMR (125 MHz, methanol-*d$_4$*)**: δ (ppm) 173.5 (br), 160.2, 141.3, 128.5, 120.1, 116.6, 112.7, 52.5, 40.6, 38.0, 34.5, 22.5.

HRMS (ESI) *m/z* calcd for $C_{12}H_{16}N_2O$ $[M + H]^+$ 221.1284, found 221.1272.

(2*R*,3*R*,4*S*,5*R*,6*R*)-2-(3-(2-acetamidoethyl)-5-acetoxyindolin-1-yl)-6-(acetoxymethyl)tetrahydro-2*H*-pyran-3,4,5-triyl triacetate (47). To a solution of **46** (209 mg, 0.953 mmol, 1.0 eq.) in TFA (1.5 mL) was added α-D-glucose (867 mg, 4.82 mmol, 5.0 eq.). The mixture was refluxed for 2 hours and concentrated *in vacuo*. The crude intermediate was redissolved in pyridine (15 mL) and acetic anhydride (8 mL, 86.7 mmol, 90 eq.) was added. The resulting mixture was stirred at room temperature for 1 hour and then diluted with water and extracted with DCM: MeOH (v/v = 95:5) for three

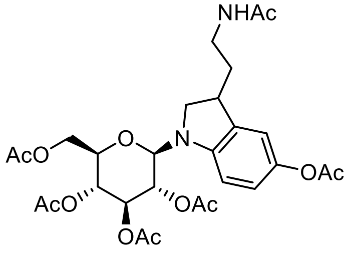

47

times. The combined organics were washed with sat. aq. $NaHCO_3$ and brine and dried with Na_2SO_4. Flash column chromatography on silica using a gradient of 0-30% isopropanol in toluene afforded **47** (mixture of diastereomers, 19.5 mg, 3.8%) as yellow oil.

^{1}H NMR (600 MHz, chloroform-*d*): δ (ppm) 6.86-6.78 (m, 2H), 6.52 (d, *J* = 8.4 Hz, 0.5H), 6.50 (d, *J* = 8.5 Hz, 0.5H), 5.67 (m, 0.5H), 5.57 (m, 0.5H), 5.33 (dt, *J* = 6.7, 9.4 Hz, 1H), 5.23 (dt, *J* = 8.2, 9.2 Hz, 1H), 5.07 (td, *J* = 3.3, 9.7 Hz, 1H), 4.91 (d, *J* = 10.0 Hz, 1H), 4.25 (ddd, *J* = 5.0, 10.9, 12.4 Hz, 1H), 4.04 (ddd, *J* = 2.4, 12.3, 17.5 Hz, 1H), 3.77-3.71 (m, 2H), 3.34-3.28 (m, 3H), 3.21 (m, 1H), 2.35 (s, 3H), 2.04 (d, *J* = 1.7 Hz, 3H), 2.03 (d, *J* = 1.7 Hz, 3H), 2.01-1.98 (6H), 1.94 (d, *J* = 11.8 Hz, 3H), 1.76-1.62 (m, 2H).

HRMS (ESI) *m/z* calcd for $C_{28}H_{36}N_2O_{12}$ $[M + H]^+$ 593.2341, found 593.2299.

(2*R*,3*R*,4*S*,5*R*,6*R*)-2-(3-(2-acetamidoethyl)-5-acetoxy-1*H*-indol-1-yl)-6-(acetoxymethyl)tetrahydro-2*H*-pyran-3,4,5-triyl triacetate (48). To a solution of **47** (19.5 mg, 0.0324 mmol, 1.0 eq.) in 1,4-dioxane (1 mL) was added DDQ (8.8 mg, 0.039 mmol, 1.2 eq.), and the mixture was stirred at room temperature. After 1.5 hours, the reaction mixture was cooled to 0 ºC ice bath, diluted with sat. aq. $NaHCO_3$, and extracted with EtOAc for three times. Combined organics were washed with brine, dried with Na_2SO_4, and then concentrated *in vacuo*. Flash column chromatography on silica using 100% DCM afforded **48** (15.2 mg, 79%).

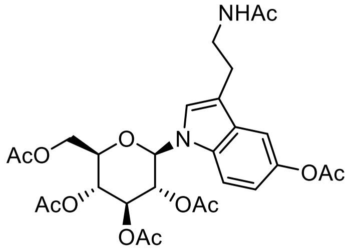

48

^{1}H NMR (600 MHz, chloroform-*d*): δ (ppm) 7.31 (d, *J* = 8.9 Hz, 1H), 7.23 (d, *J* = 2.1 Hz, 1H), 7.15 (s, 1H), 6.98 (dd, *J* = 2.1, 8.9 Hz, 1H), 5.91 (m, 1H), 5.53 (d, *J* = 9.0 Hz, 1H), 5.46 (t, *J* = 9.5 Hz, 1H), 5.35 (t, *J* = 9.4 Hz, 1H), 5.25 (t, *J* = 9.8 Hz, 1H), 4.32 (dd, *J* = 5.0, 12.6 Hz, 1H), 4.16 (dd, *J* = 2.1, 12.6 Hz, 1H), 4.11 (q, *J* = 7.2 Hz, 1H), 4.01 (ddd, *J* = 2.2, 5.0, 10.2 Hz, 1H), 3.67 (m, 1H), 3.42 (m, 1H), 2.93 (m, 1H), 2.81 (m, 1H), 2.31 (s, 3H), 2.084 (s, 3H), 2.078 (s, 3H), 2.02 (s, 3H), 1.94 (s, 3H), 1.55 (s, 3H). **^{13}C NMR (125 MHz, chloroform-*d*)**: δ (ppm) 170.7, 170.6, 170.4, 170.1, 169.6, 169.2, 144.9, 134.6, 128.8, 123.6, 117.1, 115.7, 111.8, 109.8, 83.0, 75.0, 72.8, 71.5, 68.3, 62.0, 51.0, 39.1, 23.3, 21.3, 20.9, 20.73, 20.70, 20.2.

HRMS (ESI) *m/z* calcd for $C_{28}H_{34}N_2O_{12}$ $[M + H]^+$ 591.2184, found 591.2151.

***N*-(2-(5-hydroxy-1-((2*R*,3*R*,4*S*,5*S*,6*R*)-3,4,5-trihydroxy-6-(hydroxymethyl)tetrahydro-2*H*-pyran-2-yl)-1*H*-indol-3-yl)ethyl)acetamide (sngl#101, 37).** To a solution of **48** (15.2 mg, 0.0257 mmol, 1.0 eq.) in MeOH (1.5 mL) was added 8% NaOH (0.3 mL). The mixture was stirred at room temperature for 25 min. and concentrated *in vacuo*. Flash column chromatography on silica using a gradient of 0-90% MeOH in DCM afforded **37** as clear oil (5.7 mg,

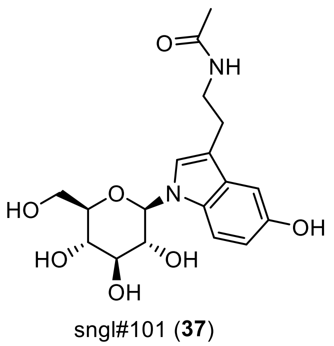

sngl#101 (**37**)

58%).

See Supplementary Table 5 for NMR spectroscopic data of sngl#101 (**37**).

HRMS (ESI) *m/z* calcd for $C_{18}H_{24}N_2O_7$ [M + Na]$^+$ 403.1476, found 403.1471.

3.5. Synthesis of sngl#1-4

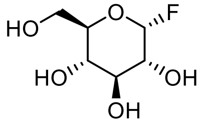

38

(2*R*,3*R*,4*S*,5*S*,6*R*)-2-fluoro-6-(hydroxymethyl)tetrahydro-2*H*-pyran-3,4,5-triol (38). 38 was prepared as previously described [4,5].

***N*-(2-(5-(((2*S*,3*R*,4*S*,5*S*,6*R*)-3,4,5-trihydroxy-6-(hydroxymethyl)tetrahydro-2*H*-pyran-2-yl)oxy)-1*H*-indol-3-yl)ethyl)acetamide (sngl#1, 29).** To a 20 mL glass vial containing **38** (1.52 g, 8.35 mmol, 3 eq.), *N*-acetylserotonin (607 mg, 2.78 mmol, 1.0 eq.) and $Ca(OH)_2$ (618 mg, 8.35 mmol, 3 eq.) was added water (3 mL). The reaction mixture was stirred vigorously for 35 minutes. The crude mixture was purified by reversed-phase flash chromatography with a C18 column using a gradient of 0-40% MeOH in H_2O, which afforded sngl#1 (**29**, 779.0 mg, 74%) as a white solid.

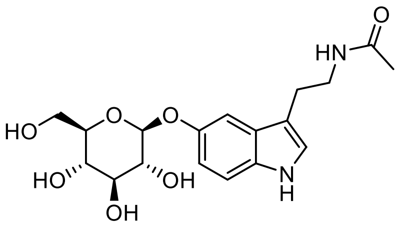

sngl#1 (**29**)

See Supplementary Table 6 for NMR spectroscopic data of sngl#1 (**29**).

HRMS (ESI) *m/z* calcd for $C_{18}H_{24}N_2NaO_7^+$ [M + Na]$^+$ 403.1476, found 403.1485.

((2*R*,3*S*,4*S*,5*R*,6*S*)-6-((3-(2-acetamidoethyl)-1*H*-indol-5-yl)oxy)-3,4,5-trihydroxytetrahydro-2*H*-pyran-2-yl)methyl 2-aminobenzoate (sngl#3, 31). To a mixture of DCM/DMF (3 mL, v/v = 1:2) was added Boc-2-aminobenzoic acid (15.4 mg, 0.065 mmol, 1.2 eq.) and EDC·HCl (31.2 mg, 0.163 mmol, 3.0 eq.). The mixture was stirred at room temperature for 30 minutes, and DMAP (26.5 mg, 0.217 mmol, 4.0 eq.) and sngl#1 (**29**, 20.6 mg, 0.0542 mmol, 1.0 eq.) were added. After 5 days, the reaction mixture was concentrated *in vacuo*. Flash column chromatography on silica using a gradient of 0-30% MeOH in DCM afforded intermediate **49** (4.1 mg, 13%). Intermediate **49** was redissolved in DCM (1 mL), followed by slow addition of TFA (0.1 mL). The reaction mixture was stirred at room temperature for 1.5 hours and

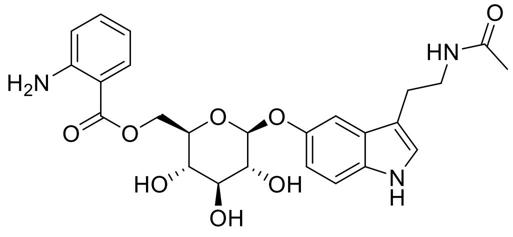

sngl#3 (**31**)

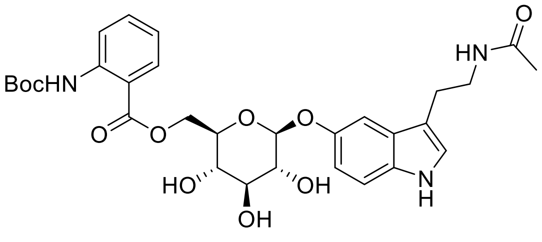

49

concentrated *in vacuo*. Preparative HPLC provided a pure sample of sngl#3 (**31**, 0.3 mg, 1.1 %).

See Supplementary Table 8 for NMR spectroscopic data of sngl#3 (**31**).

HRMS (ESI) *m/z* calcd for $C_{25}H_{29}N_3O_8$ $[M + H]^+$ 500.2027, found 500.2005.

***N*-(2-(5-(((6a*R*,8*S*,9*R*,10*R*,10a*S*)-9,10-dihydroxy-2,2,4,4-tetraisopropylhexahydropyrano[3,2-f][1,3,5,2,4]trioxadisilocin-8-yl)oxy)-1*H*-indol-3-yl)ethyl)acetamide (50).** To a solution of sngl#1 (**29**, 194 mg, 0.511 mmol, 1.0 eq.) in DMF was added imidazole (152 mg, 1.84 mmol, 4.4 eq.) was cooled to 0 °C before $TIPDSiCl_2$ (228 µL, 0.713 mmol, 1.4 eq.) was added. The reaction mixture was allowed to warm to room temperature over 1.5 hours and stirred for another 30 minutes. The mixture was then diluted with DCM and quenched with water. The organics were washed with sat. aq. $NaHCO_3$, dried with Na_2SO_4, and concentrated *in vacuo*. Flash column chromatography on silica using a gradient of 0-10% MeOH in DCM afforded **50** as a white solid (227.6 mg, 72%).

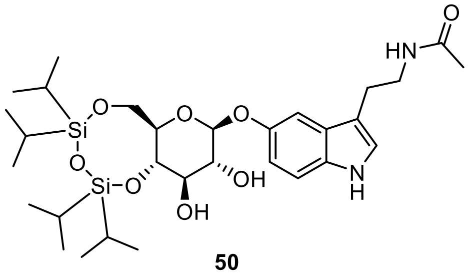

50

^{1}H NMR (500 MHz, chloroform-*d*): δ (ppm) 8.16 (s, 1H), 7.25-7.20 (m, 2H), 7.04-6.95 (m, 2H), 4.89 (d, *J* = 7.3 Hz, 1H), 4.13 (d, *J* = 11.9 Hz, 1H), 4.01 (d, *J* = 12.5 Hz, 1H), 3.93 (t, *J* = 8.9 Hz, 1H), 3.75-3.64 (m. 2H), 3.52 (m, 2H), 3.36 (m, 1H), 3.88 (m, 2H), 1.94 (s, 3H), 1.10-0.99 (m, 28H).

HRMS (ESI) *m/z* calcd for $C_{30}H_{50}N_2O_8Si_2$, $[M + H]^+$ 623.3178, found 623.3157.

(6a*R*,8*S*,9*R*,10*R*,10a*S*)-8-((3-(2-acetamidoethyl)-1*H*-indol-5-yl)oxy)-10-hydroxy-2,2,4,4-tetraisopropylhexahydropyrano[3,2-f][1,3,5,2,4]trioxadisilocin-9-yl benzyl carbonate (51). To a solution of **50** (227 mg, 0.365 mmol, 1.0 eq.) in DCM was added DMAP (147 mg, 1.20 mmol, 3.3 eq.) and DMF (50 µL). The mixture was cooled to 0 °C before added benzyl chloroformate (233 µL, 1.64 mmol, 4.5 eq.). The reaction mixture was allowed to warm to room temperature within 30 minutes and stirred for another 1.3 hours. The mixture was diluted with DCM and then quenched with water. The aqueous layer was separated and extracted with DCM for three times. The combined organics were washed with sat. aq. $NaHCO_3$ and brine, dried with Na_2SO_4, and concentrated *in vacuo*. Flash column chromatography of the residue on silica using a gradient of 0-20% isopropanol in toluene afforded **51** as a white solid (196.1 mg, 66%).

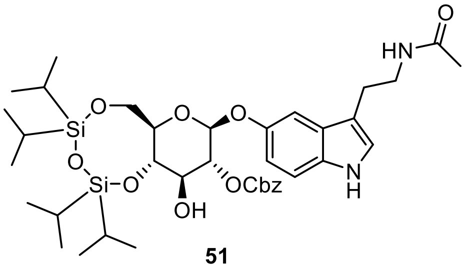

51

^{1}H NMR (600 MHz, chloroform-*d*): δ (ppm) 8.22 (s, 1H), 7.40-7.37 (m, 2H), 7.36-7.31 (m, 3H), 7.19-7.16(m, 2H), 6.99 (s, 1H), 6.82 (dd, *J* = 2.2, 8.7 Hz, 1H), 5.64 (m, 1H), 5.26 (d, *J* = 12.1 Hz,

1H), 5.21 (d, *J* = 12.1 Hz, 1H), 4.97 (d, *J* = 8.0 Hz, 1H), 4.93 (dd, *J* = 8.7, 9.3 Hz, 1H), 4.12 (dd, *J* = 1.9, 12.7 Hz, 1H), 4.05 (dd, *J* = 1.2, 12.7 Hz, 1H), 3.98 (t, *J* = 1.2, 9.3 Hz, 1H), 3.81 (t, *J* = 1.2, 9.1 Hz, 1H), 3.51-3.47 (m, 2H), 3.34 (dt, *J* = 1.2, 9.4 Hz, 1H), 2.83 (t, *J* = 6.6 Hz, 2H), 1.88 (s, 3H), 1.14-1.01 (m, 28H). **^{13}C NMR (125 MHz, chloroform-*d*)**: δ (ppm) 170.5, 155.1, 151.7, 135.2, 133.1, 128.74, 128.68, 128.48, 128.35, 127.8, 126.4, 114.5, 113.0, 111.8, 106.6, 101.5, 77.9, 76.7, 75.2, 70.2, 69.7, 60.9, 39.8, 25.2, 23.3, 17.57, 17.47, 17.43, 17.37, 17.33, 17.31, 17.25, 13.7, 13.3, 12.7, 12.6.

HRMS (ESI) *m/z* calcd for $C_{38}H_{56}N_2O_{10}Si_2$ $[M + H]^+$ 757.3546, found 757.3517.

(6a*R*,8*S*,9*R*,10*R*,10a*R*)-8-((3-(2-acetamidoethyl)-1*H*-indol-5-yl)oxy)-10-((bis(benzyloxy)phosphoryl)oxy)-2,2,4,4-tetraisopropylhexahydropyrano[3,2-f][1,3,5,2,4]trioxadisilocin-9-yl benzyl carbonate (52). To a solution of **51** (145.8 mg, 0.188 mmol, 1.0 eq.) in DCM was added dibenzyl *N*,*N*-diisopropylphosphoramidite (221 μL, 0.659 mmol, 3.5 eq.) and 1*H*-tetrazole (0.45 M in ACN, 1.5 mL, 0.659 mmol, 3.5 eq.). The reaction mixture was stirred at room temperature for 1 hour. The solution was cooled to -78 °C under argon, and *m*-CPBA (≤77%, 143.0 mg, 0.638 mmol, 3.4 eq.) in DCM (1.5 mL) was added slowly to the reaction mixture. The solution was stirred at -78 °C for 0.5 hour, and slowly warmed to room temperature and reacted for another 1 hour, then was diluted with DCM and washed with 10% Na_2SO_4 twice, sat. aq. $NaHCO_3$, and brine, dried with Na_2SO_4, and concentrated *in vacuo*. Flash column chromatography on silica using a gradient of 5-100% EtOAc in hexanes afforded **52** as a white solid (141.3 mg, 74%).

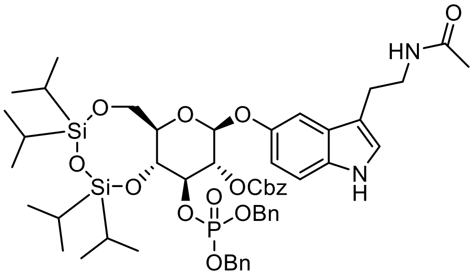

52

^{1}H NMR (500 MHz, chloroform-*d*): δ (ppm) 8.31 (s, 1H), 7.36-7.27 (m, 15H), 7.22-7.18 (m, 2H), 7.04 (d, *J* = 2.2 Hz, 1H), 6.78 (dd, *J* = 2.2, 8.7 Hz, 1H), 5.53 (m, 1H), 5.23 (d, *J* = 12.2 Hz, 1H), 5.13 (dd, *J* = 8.0, 9.4 Hz, 1H), 5.08-4.91 (m, 6H), 4.61 (dt, *J* = 8.6, 8.9 Hz, 1H), 4.20-4.14 (m, 2H), 4.09 (d, *J* = 12.6 Hz, 1H), 3.59-3.47 (m, 2H), 3.33 (dt, *J* = 1.7, 9.4 Hz, 1H), 2.87 (t, *J* = 6.6 Hz, 2H), 1.92 (s, 3H), 1.16-0.99 (m, 28H). **^{13}C NMR (125 MHz, chloroform-*d*)**: 170.2, 154.6, 151.7, 136.14, 136.08, 135.90, 135.85, 135.3, 133.2, 128.60, 128.57, 128.53, 128.49, 128.36, 128.12, 128.06, 127.8, 123.3, 114.6, 113.1, 111.8, 106.8, 101.6, 80.3 (d, *J* = 6.5 Hz), 76.6 (d, *J* = 4.6 Hz), 70.0, 69.6 (t, *J* = 5.9 Hz), 68.7 (d, *J* = 5.2 Hz), 60.9, 39.8, 25.3, 23.5, 17.54, 17.50, 17.46, 17.41, 17.36, 17.34, 17.28, 17.11, 13.35, 13.26, 12.97, 12.95.

HRMS (ESI) *m/z* calcd for $C_{52}H_{69}N_2O_{13}PSi_2$ $[M + H]^+$ 1017.4149, found 1017.4105.

(2*S*,3*R*,4*S*,5*R*,6*R*)-2-((3-(2-acetamidoethyl)-1*H*-indol-5-yl)oxy)-4-((bis(benzyloxy)phosphoryl)oxy)-5-hydroxy-6-(hydroxymethyl)tetrahydro-2*H*-pyran-3-yl benzyl carbonate (53). To a solution of **52** (141.3 mg, 0.139 mmol, 1.0 eq.) in THF (6 mL) was added acetic acid (24 µL, 0.417 mmol, 3.0 eq.). The solution was cooled to -10 °C before tetrabutylammonium fluoride solution (1M in THF, 417 µL, 00.417mmol, 3.0 eq.) was added. The reaction mixture was stirred for 1.5 hours in cold and concentrated *in vacuo*. Flash column chromatography on silica using a gradient of 0-15% MeOH in DCM afforded **53** as a white solid (92.3 mg, 86%).

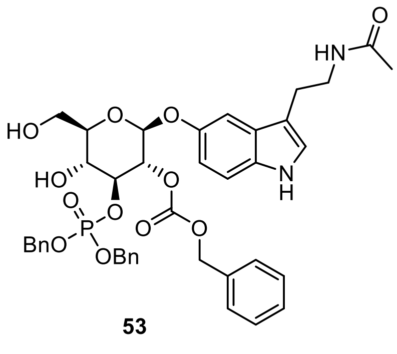

53

^{1}H NMR (500 MHz, chloroform-*d*): δ (ppm) 8.32 (s, 1H), 7.36-7.21 (m, 15H), 7.14 (d, *J* = 8.7 Hz, 1H), 6.96 (d, *J* = 2.1 Hz, 1Hd), 6.71 (dd, *J* = 2.1, 8.7 Hz, 1H), 5.82 (m, 1H), 5.12 (d, *J* = 12.2 Hz, 1H), 5.10-4.94 (m, 7H), 4.49 (dt, *J* = 7.2, 8.9 Hz, 1H), 3.99 (dd, *J* = 2.8, 12.2 Hz, 1H), 3.84-3.74 (m, 2H), 3.55-3.46 (m, 2H), 3.40 (m, 1H), 2.91-2.77 (m, 2H), 1.89 (s, 3H). **^{13}C NMR (125 MHz, chloroform-*d*)**: δ (ppm) 171.1, 154.5, 151.2, 135.0, 133.1, 128.81, 128.75, 128.72, 128.71, 128.69, 128.66, 123.4, 114.2, 113.1, 111.8, 106.9, 100.6, 81.7 (d, *J* = 5.6 Hz), 76.17, 76.0 (d, *J* = 6.2 Hz), 70.4, 70.25 (d, *J* = 6.0 Hz), 70.17, 70.13 (d, *J* = 6.0 Hz), 62.3, 40.3, 25.4, 23.4.

HRMS (ESI) *m/z* calcd for $C_{40}H_{43}N_2O_{12}P$ [M - H]$^-$ 773.2481, found 773.2488.

(2*S*,3*R*,4*S*,5*R*,6*R*)-2-((3-(2-acetamidoethyl)-1H-indol-5-yl)oxy)-3,5-dihydroxy-6-(hydroxymethyl)tetrahydro-2*H*-pyran-4-yl dihydrogen phosphate (30) To a solution of **53** (26.9 mg, 0.0347 mmol, 1.0 eq.) in a mixture of MeOH and EtOAc (2 mL, v/v = 1:1) was added Pd/C (10% w/w, 18 mg). The reaction mixture was purged with argon for 5 minutes, flushed with hydrogen, and then subjected to hydrogen atmosphere for 1.5 hours at room temperature, and subsequently again purged with argon for 5 minutes. The mixture was filtered through celite and concentrated *in vacuo*. The crude mixture was purified by reversed-phase flash chromatography with a C18 column using a gradient of 0-10% ACN in H_2O with 0.1% formic acid, which afforded sngl#2 as a clear oil (**30**, 9.3 mg, 58%).

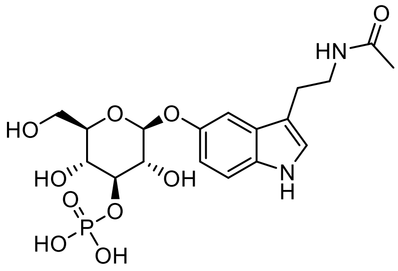

sngl#2 (**30**)

See Supplementary Table 7 for NMR spectroscopic data of sngl#2 (**30**).

HRMS (ESI) *m/z* calcd for $C_{18}H_{25}N_2O_{10}P$ [M - H]$^-$ 459.1174, found 459.1185.

((2*R*,3*R*,4*S*,5*R*,6*S*)-6-((3-(2-acetamidoethyl)-1H-indol-5-yl)oxy)-5-(((benzyloxy)carbonyl)oxy)-4-((bis(benzyloxy)phosphoryl)oxy)-3-hydroxytetrahydro-2*H*-pyran-2-yl)methyl 2-((*tert*-butoxycarbonyl)amino)benzoate (54). To a mixture of dry DCM/DMF (2 mL, v/v = 100:1) was added Boc-2-aminobenzoic acid (70.7 mg, 0.298 mmol, 2.5 eq.) and EDC·HCl (68.4 mg, 0.444 mmol, 3.0 eq.). The mixture was stirred at room temperature for 25 minutes, and DMAP (58.2 mg, 0.476 mmol, 4.0 eq.) and **53** (92.3 mg, 0.119 mmol, 1.0 eq.) were added. After 25 hours, the reaction mixture was concentrated *in vacuo*. Flash column chromatography on silica using a gradient of 0-12% MeOH in DCM afforded **54** as a white solid (72.0 mg, 61%).

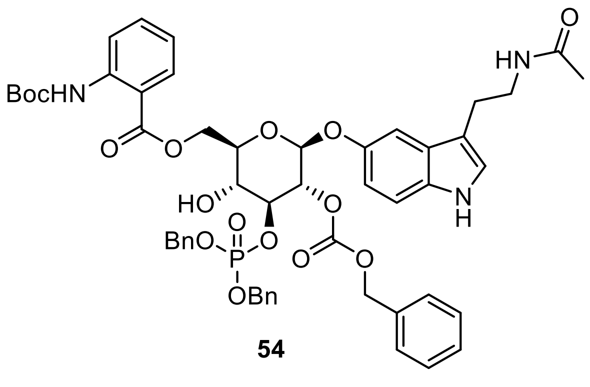

54

^{1}H NMR (600 MHz, chloroform-*d*): δ (ppm) 8.42 (d, *J* = 8.4 Hz, 1H), 7.97 (dd, *J* = 1.1, 8.0 Hz, 1H), 7.49 (dd, *J* = 1.1, 7.8 Hz, 1H), 7.33-7.15 (m, 16H), 7.09 (d, *J* = 8.7 Hz, 1H), 7.00 (d, *J* = 1.1 Hz, 1H), 6.91 (t, *J* = 7.8 Hz, 1H), 6.82 (dd, *J* = 2.0, 8.7 Hz, 1H), 5.61 (m, 1H), 5.15-4.95 (m, 8H), 4.71 (dd, *J* = 2.0, 12.0 Hz, 1H), 4.56 (dd, *J* = 6.1, 12.0 Hz, 1H), 4.49 (m, 1H), 3.84 (t, *J* = 9.4 Hz, 1H), 3.77 (m, 1H), 3.49-3.44 (m, 2H), 2.75 (t, *J* = 6.9 Hz, 2H), 1.90 (s, 3H), 1.50 (s, 9H).

HRMS (ESI) *m/z* calcd for $C_{52}H_{56}N_3O_{15}P$ $[M + H]^+$ 994.3522, found 994.3489.

((2*R*,3*R*,4*S*,5*R*,6*S*)-6-((3-(2-acetamidoethyl)-1H-indol-5-yl)oxy)-5-(((benzyloxy)carbonyl)oxy)-4-((bis(benzyloxy)phosphoryl)oxy)-3-hydroxytetrahydro-2*H*-pyran-2-yl)methyl 2-aminobenzoate (55). To a solution of **54** (72.0 mg, 72.5 μmol, 1.0 eq.) in DCM (2 mL) was added TFA (200 μL). The yellow mixture was stirred at room temperature for 1 hour and turned purple. The reaction mixture was then concentrated *in vacuo*. Flash column chromatography on silica using a gradient of 0-10% MeOH in DCM afforded **55** (54.9 mg, 85%).

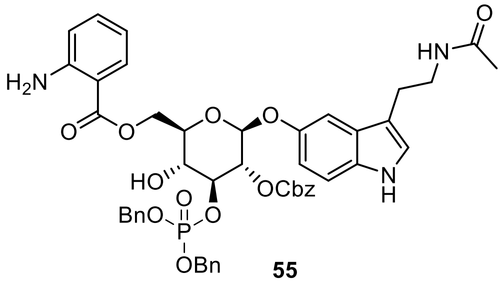

55

^{1}H NMR (600 MHz, acetone-*d*$_6$): δ (ppm) 7.89 (dd, *J* = 1.5, 8.1 Hz, 1H), 7.40-7.24 (m, 17H), 7.22 (d, *J* = 8.5 Hz, 1H), 7.15 (s, 1H), 6.84 (dd, *J* = 2.3, 8.7 Hz, 1H), 6.80 (dd, *J* = 0.6, 8.3 Hz, 1H), 6.56 (ddd, *J* = 1.1, 7.1, 8.3 Hz, 1H), 5.32 (d, *J* = 8.1 Hz, 1H), 5.24 (d, *J* = 12.2 Hz, 1H), 5.17-5.03 (m, 6H), 4.84 (m, 1H), 4.75 (dd, *J* = 1.2, 12.2 Hz, 1H), 4.53 (dd, *J* = 5.6, 11.8 Hz, 1H), 4.10 (m, 1H), 4.03 (t, *J* = 9.1 Hz, 1H), 3.50-3.39 (m, 2H), 2.83 (t, *J* = 7.2 Hz, 2H), 1.87 (s, 3H). **^{13}C NMR (125 MHz, acetone-*d*$_6$)**: δ (ppm) 168.4, 155.4, 152.4, 152.0, 137.1, 136.5, 132.0, 129.34, 129.32, 129.26, 129.18, 129.13, 129.07, 129.04, 128.84, 128.81, 128.68, 124.44, 117.3, 116.1, 113.8, 113.5, 112.5, 110.4, 106.8, 101.1, 81.6 (d, *J* = 5.8

Hz), 77.1 (d, J = 4.6 Hz), 74.6, 70.54, 70.46, 70.3 (d, J = 5.5 Hz), 70.2 (d, J = 5.5 Hz), 63.7, 40.4, 26.3, 23.0.

HRMS (ESI) *m/z* calcd for $C_{47}H_{56}N_3O_{15}P$ $[M + H]^+$ 894.2998, found 894.2957.

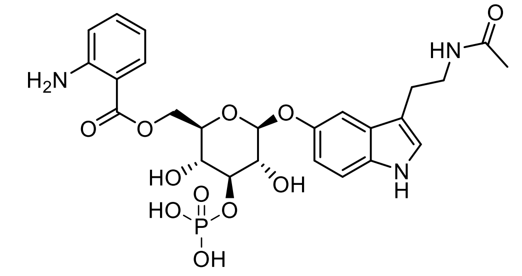

sngl#4 (**32**)

((2*R*,3*R*,4*S*,5*R*,6*S*)-6-((3-(2-acetamidoethyl)-1*H*-indol-5-yl)oxy)-3,5-dihydroxy-4-(phosphonooxy)tetrahydro-2*H*-pyran-2-yl)methyl 2-aminobenzoate (sngl#4, 32). To a solution of **55** (54.9 mg, 61.4 µmol, 1.0 eq.) in a mixture of MeOH and EtOAc (2.5 mL, v/v = 2:3) was added Pd/C (10% w/w) (32 mg). The reaction mixture was purged with argon for 5 minutes, flushed with hydrogen, and then subjected to hydrogen atmosphere for 3 hours at room temperature, and again purged with argon for 5 minutes. The mixture was filtered through celite and concentrated *in vacuo*, affording sngl#4 (**32**, 33.4 mg, 94%).

See Supplementary Table 9 for NMR spectroscopic data of sngl#4 (**32**).

HRMS (ESI) *m/z* calcd for $C_{33}H_{36}N_3O_{13}P$, $[M - H]^-$ 578.1545, found 578.1554.

2.6. Synthesis of dansyl-modified serotonin 41 and 42

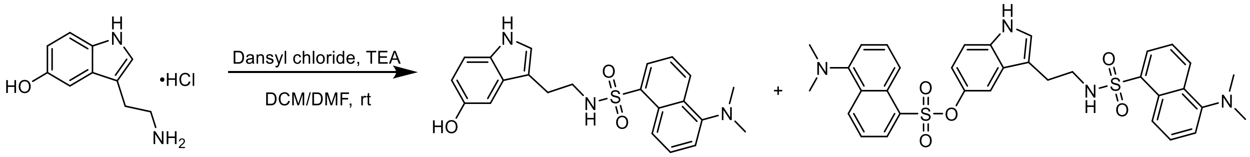

41 **42**

5-(Dimethylamino)-*N*-(2-(5-hydroxy-1*H*-indol-3-yl)ethyl)naphthalene-1-sulfonamide (41).

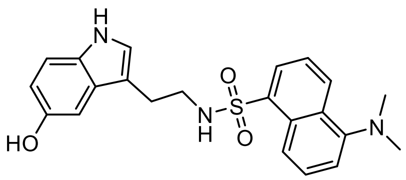

41

To a suspension of serotonin hydrochloride (60.0 mg, 0.282 mmol, 1.2 eq.) and dansyl chloride (63.4 mg, 0.235 mmol, 1.0 eq.) in a mixture of DCM and DMF (3.5 mL, v/v = 6:1) was added TEA (49 µL, 0.352 mmol, 1.5 eq.). The solution was stirred at room temperature for 1 hour and quenched with water. The mixture was extracted with DCM twice, washed with sat. aq. $NaHCO_3$ and brine, dried with Na_2SO_4, and concentrated *in vacuo*. Flash column chromatography on silica using a gradient of 0-10% methanol in DCM afforded **41** (40.2 mg, 42%) as a yellow-green oil.

^{1}H NMR (600 MHz, chloroform-*d*): δ (ppm) 8.51 (dt, J = 0.8, 8.6 Hz, 1H), 8.20 (dd, J = 1.2, 7.3 Hz, 1H), 8.16 (dt, J = 0.8, 8.6 Hz, 1H), 8.06 (br, 1H), 7.47 (dd, J = 7.3, 8.4 Hz, 1H), 7.43 (dd, J = 7.6, 8.4 Hz, 1H), 7.14 (dd, J = 0.6, 7.6 Hz, 1H), 7.12 (dd, J = 0.6, 8.6 Hz, 1H), 6.75 (ddd, J = 0.4,

2.4, 8.6 Hz, 1H), 6.72 (m, 2H), 6.24 (s, 1H), 4.93 (t, *J* = 6.0 Hz, 1H), 3.16 (dt, *J* = 6.2, 6.8 Hz, 1H), 2.89 (s, 6H), 2.71 (t, *J* = 6.8 Hz, 1H). **^{13}C NMR (125 MHz, chloroform-*d*)**: δ (ppm) 151.9, 150.0, 134.8, 131.5, 130.4, 129.9, 129.60, 129.58, 128.3, 127.6, 123.6, 123.3, 118.8, 115.3, 112.3, 111.9, 110.8, 102.9, 45.5, 43.3, 25.6.

HRMS (ESI) *m/z* calcd for $C_{22}H_{23}N_3O_3S$, $[M + H]^+$ 410.1533, found 410.1521.

3-(2-((5-(Dimethylamino)naphthalene)-1-sulfonamido)ethyl)-1*H*-indol-5-yl 5-(dimethylamino)naphthalene-1-sulfonate (42). **42** was obtained as a side product in the synthesis of **41** described above. Flash column chromatography on silica using a gradient of 0-5% methanol in DCM afforded **42** (4.4 mg, 3%) as a yellow-green oil.

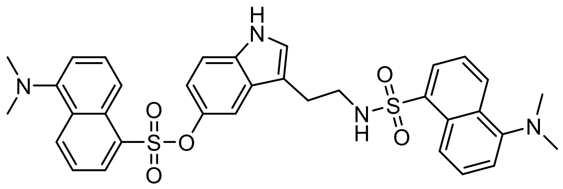

42

^{1}H NMR (600 MHz, chloroform-*d*): δ (ppm) 8.53 (d, *J* = 8.7 Hz, 2H), 8.50 (d, *J* = 8.7 Hz, 1H), 8.18 (dd, *J* = 1.2, 7.2 Hz, 1H), 8.11 (d, *J* = 8.7 Hz, 1H), 7.97 (dd, *J* = 1.2, 7.2 Hz, 1H), 7.89 (br, 1H), 7.68 (dd, *J* = 7.6, 8.5 Hz, 1H), 7.49 (dd, *J* = 7.4, 8.5 Hz, 1H), 7.44 (dd, *J* = 7.6, 8.5 Hz, 1H), 7.29 (t, *J* = 7.8 Hz, 1H), 7.25 (1H), 7.15 (d, *J* = 7.6 Hz, 1H), 7.03 (t, *J* = 8.8 Hz, 1H), 6.76 (s, 1H), 6.74 (d, *J* = 1.6 Hz, 1H), 6.65 (dd, *J* = 2.3, 8.8 Hz, 1H), 4.54 (t, *J* = 5.9 Hz, 1H), 2.97 (dt, *J* = 6.4, 6.7 Hz, 1H), 2.90 (s, 12H), 2.59 (t, *J* = 6.4Hz, 1H). **^{13}C NMR (125 MHz, chloroform-*d*)**: δ (ppm) 151.9, 143.4, 134.73, 134.67, 131.7, 131.4, 131.3, 130.6, 130.4, 129.8, 129.6, 129.1, 128.4, 126.9, 124.3, 123.3, 123.1, 120.0, 116.9, 115.7, 115.3, 112.2, 111.7, 45.62, 45.59, 42.9, 25.5.

HRMS (ESI) *m/z* calcd for $C_{34}H_{34}N_4O_5S_2$, $[M + H]^+$ 643.2043, found 643.2024.

4. NMR Spectra

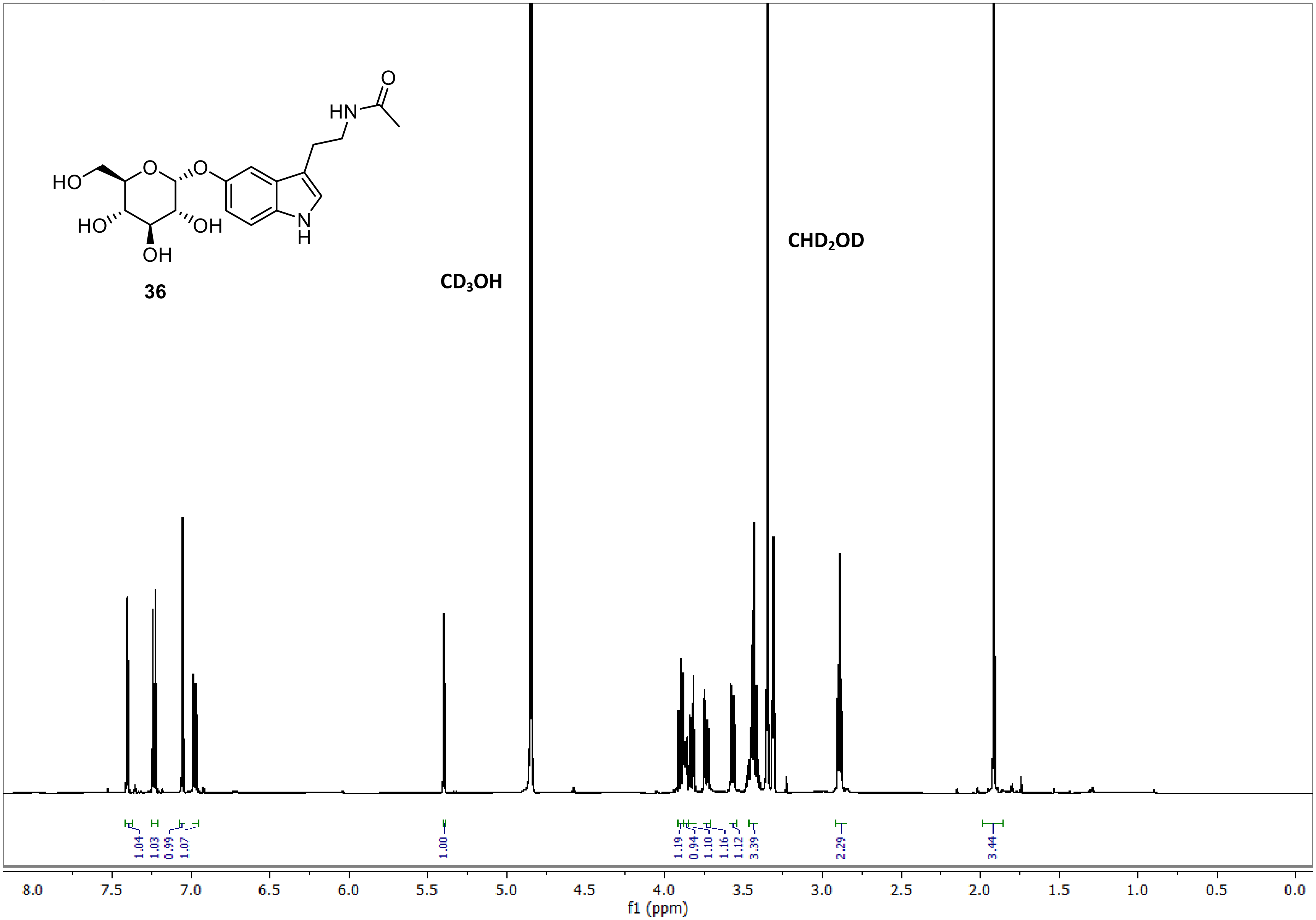

^{1}H NMR spectrum (600 MHz) of **36** in methanol-d_4.

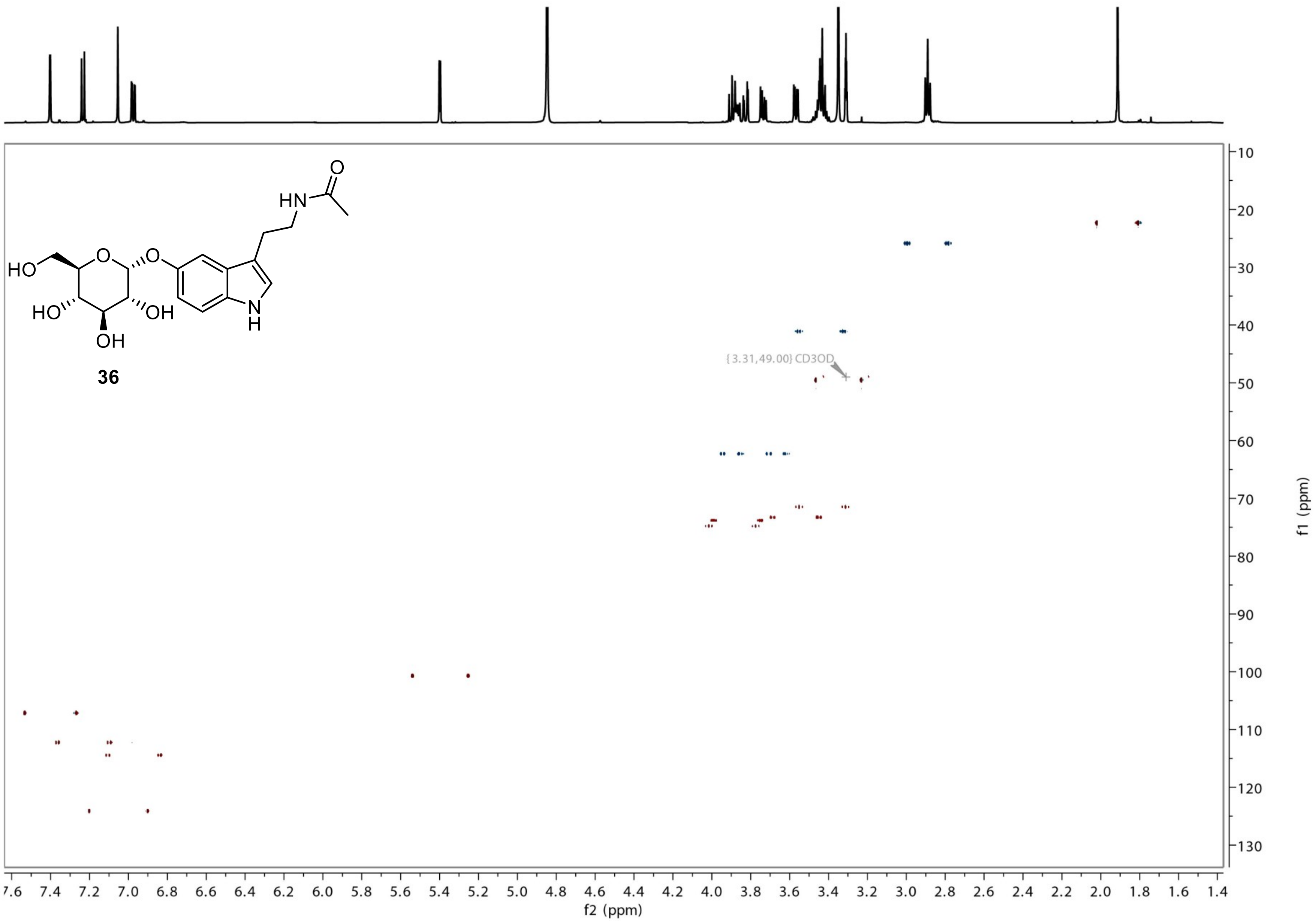

HSQC spectrum (600 MHz) of **36** in methanol-d_4.

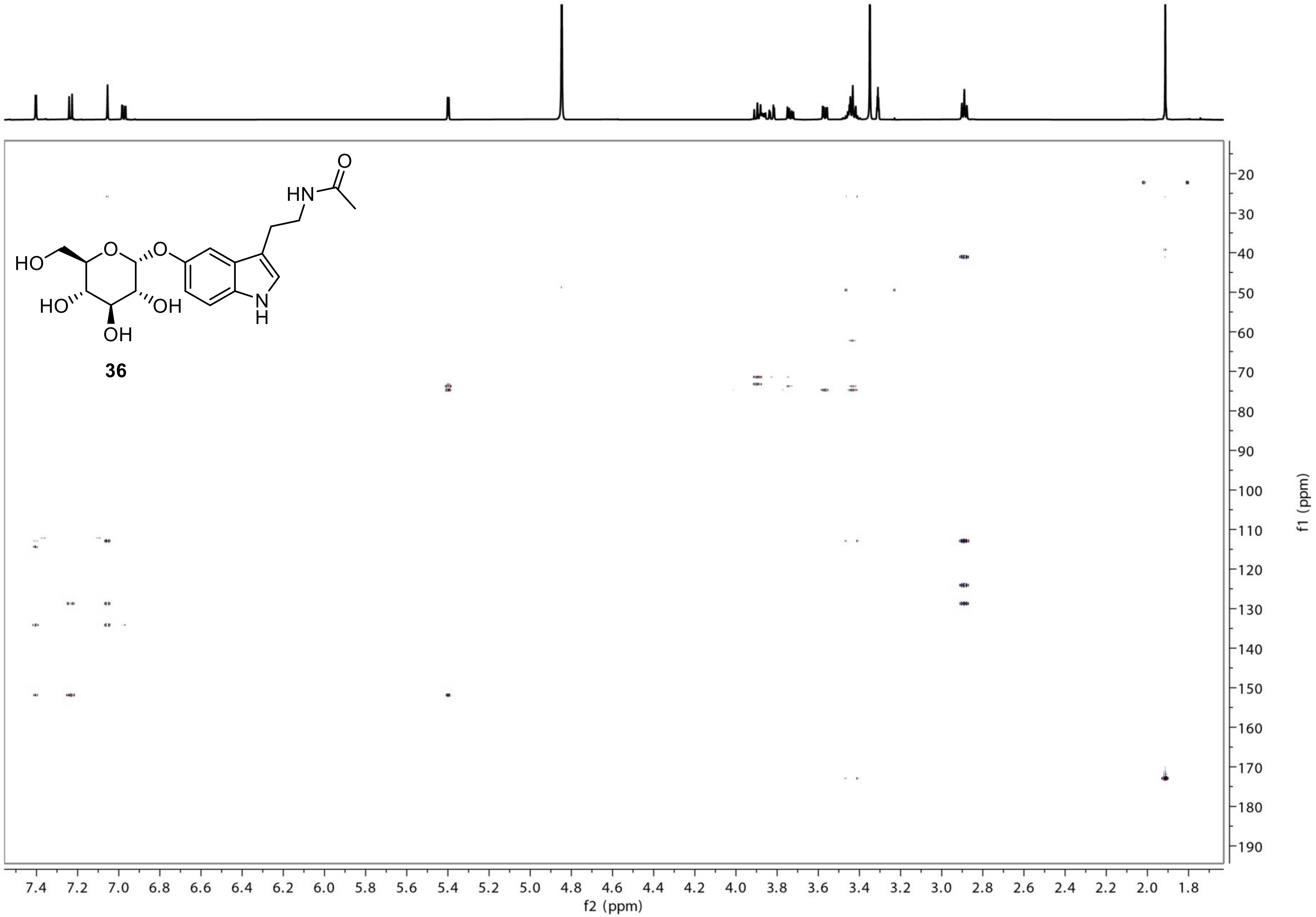

HMBC spectrum (600 MHz) of **36** in methanol-d_4.

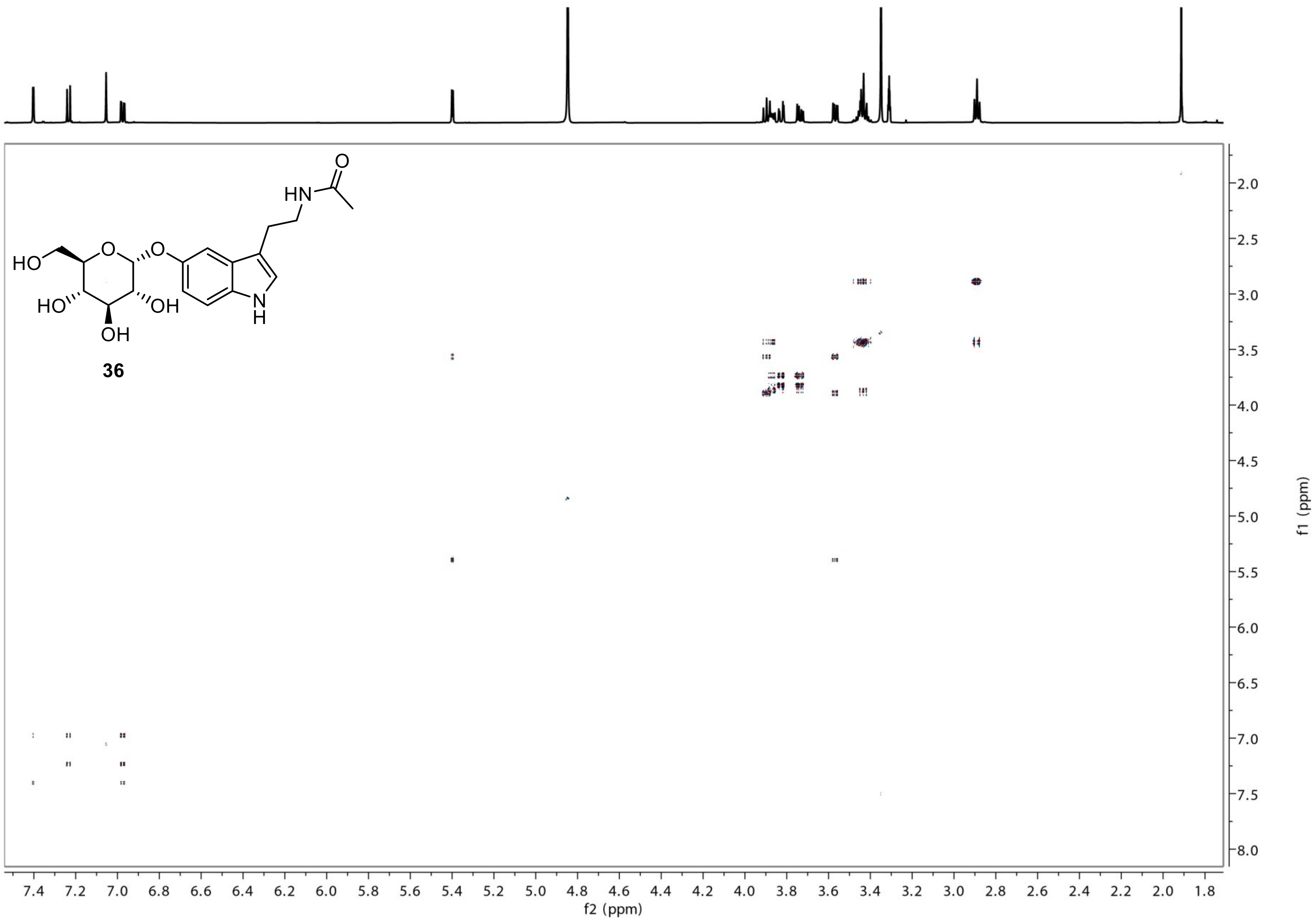

dqfCOSY spectrum (600 MHz) of **36** in methanol-d_4.

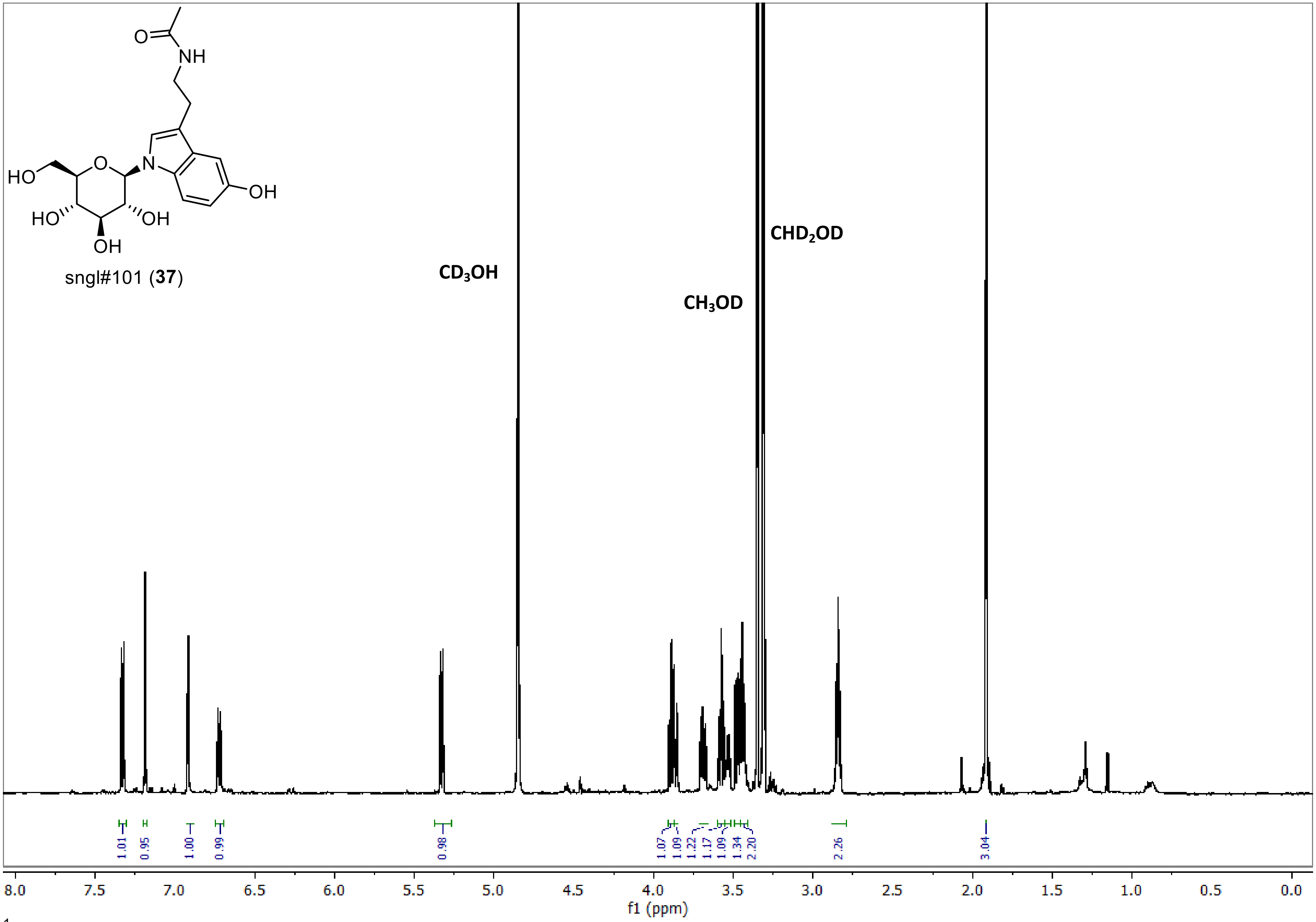

^{1}H NMR spectrum (600 MHz) of sngl#101 (**37**) in methanol-d_4.

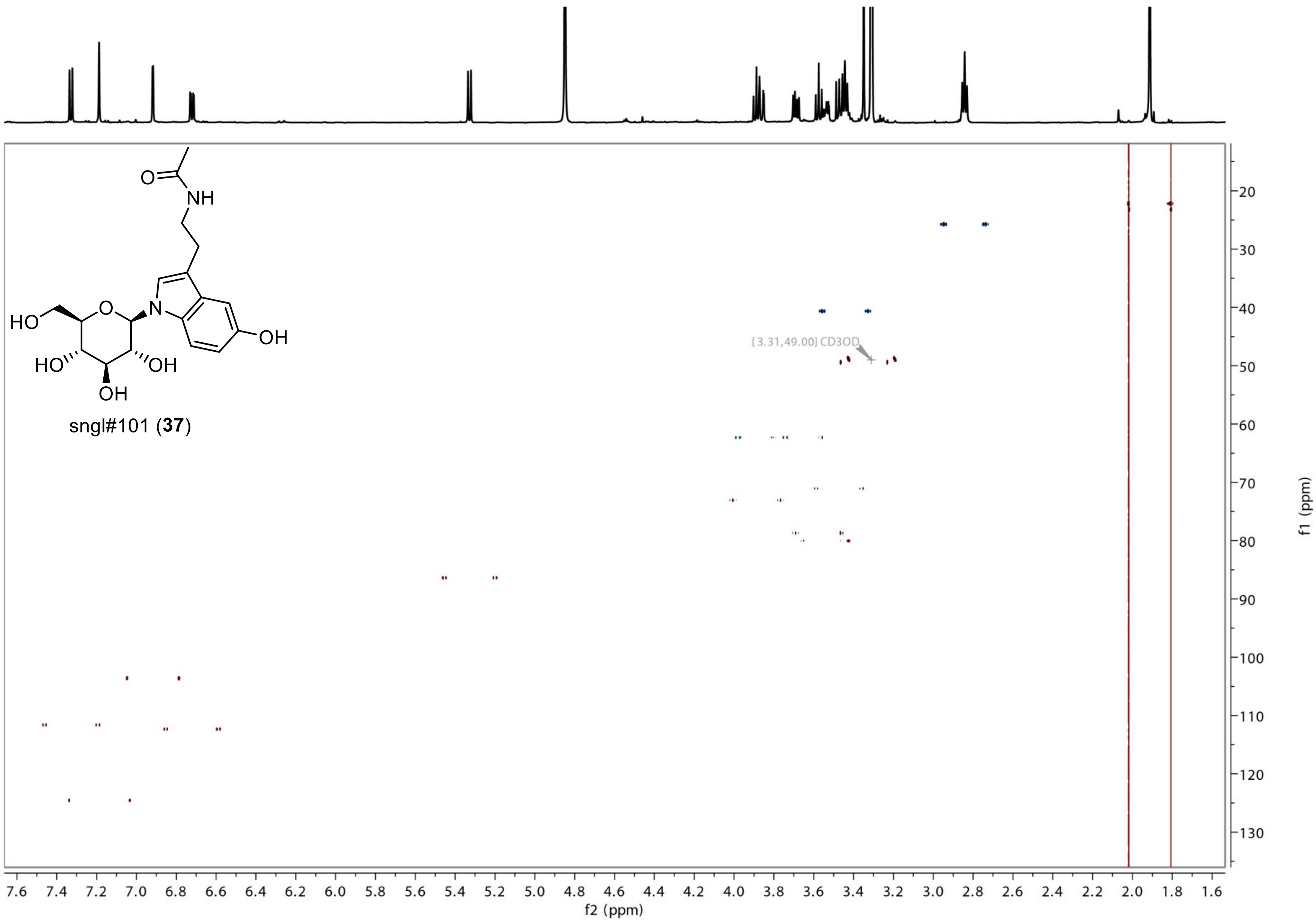

HSQC spectrum (600 MHz) of sngl#101 (**37**) in methanol-d_4.

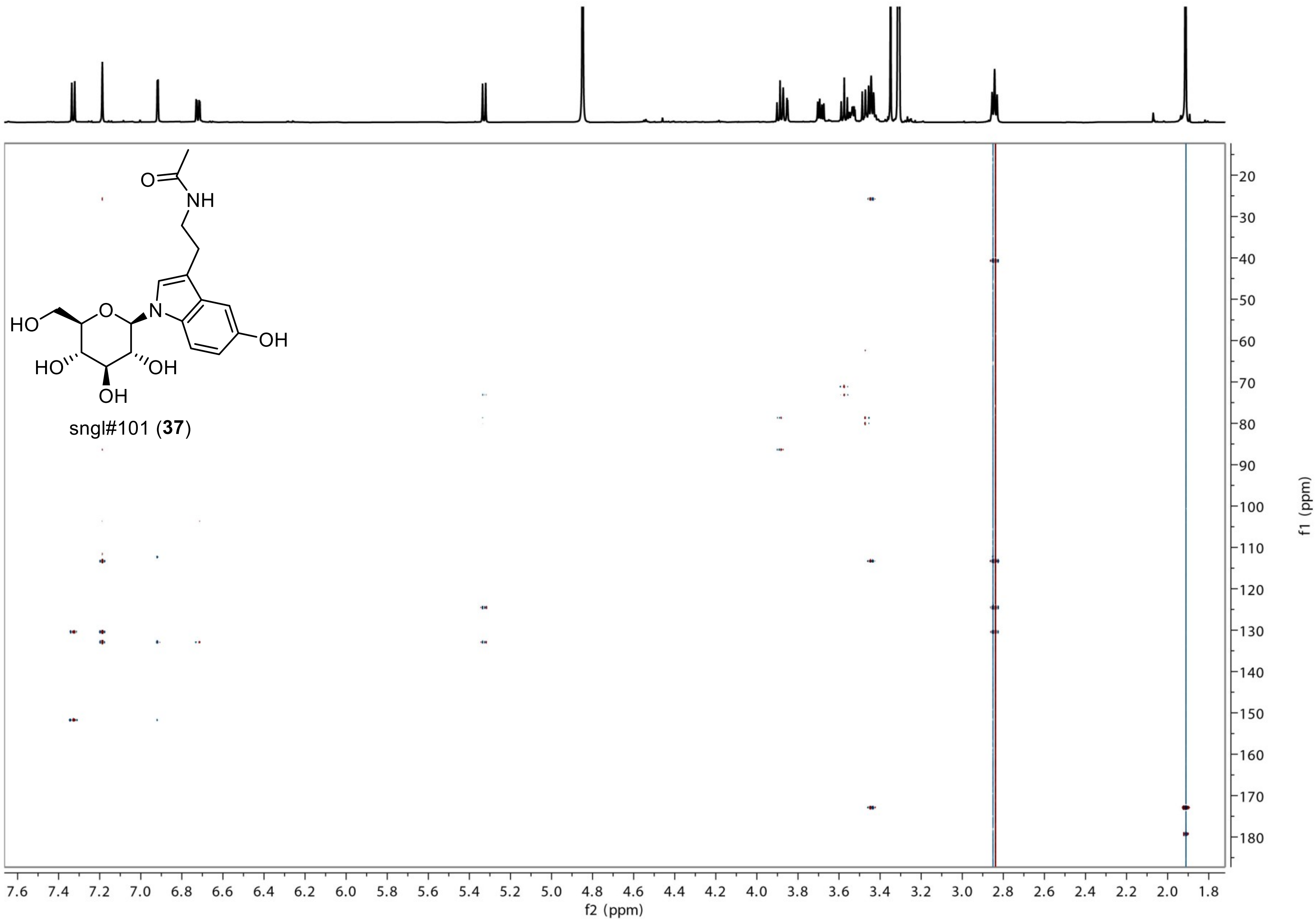

sngl#101 (**37**)

HMBC spectrum (600 MHz) of sngl#101 (**37**) in methanol-d_4.

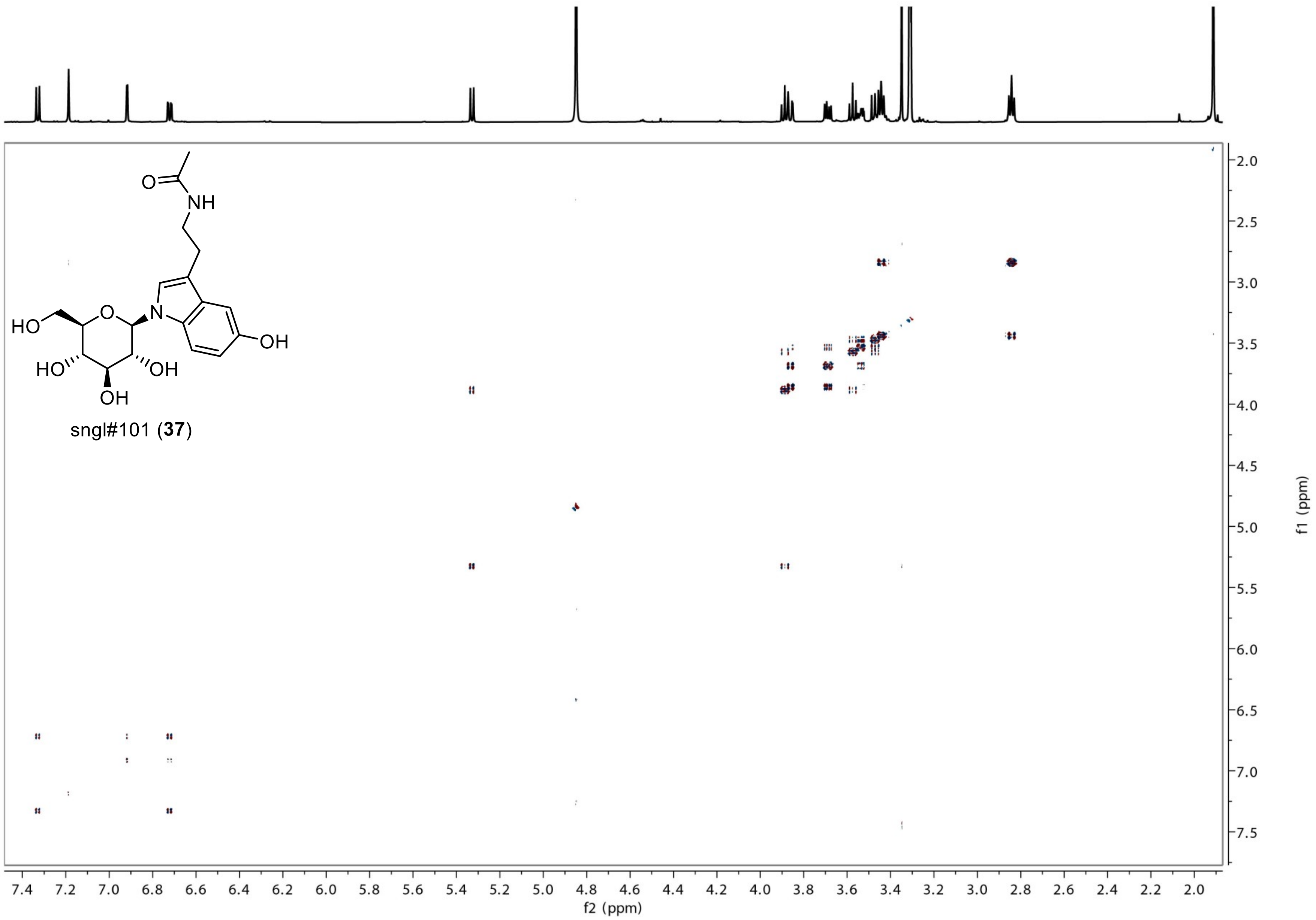

dqfCOSY spectrum (600 MHz) of sngl#101 (**37**) in methanol-d_4.

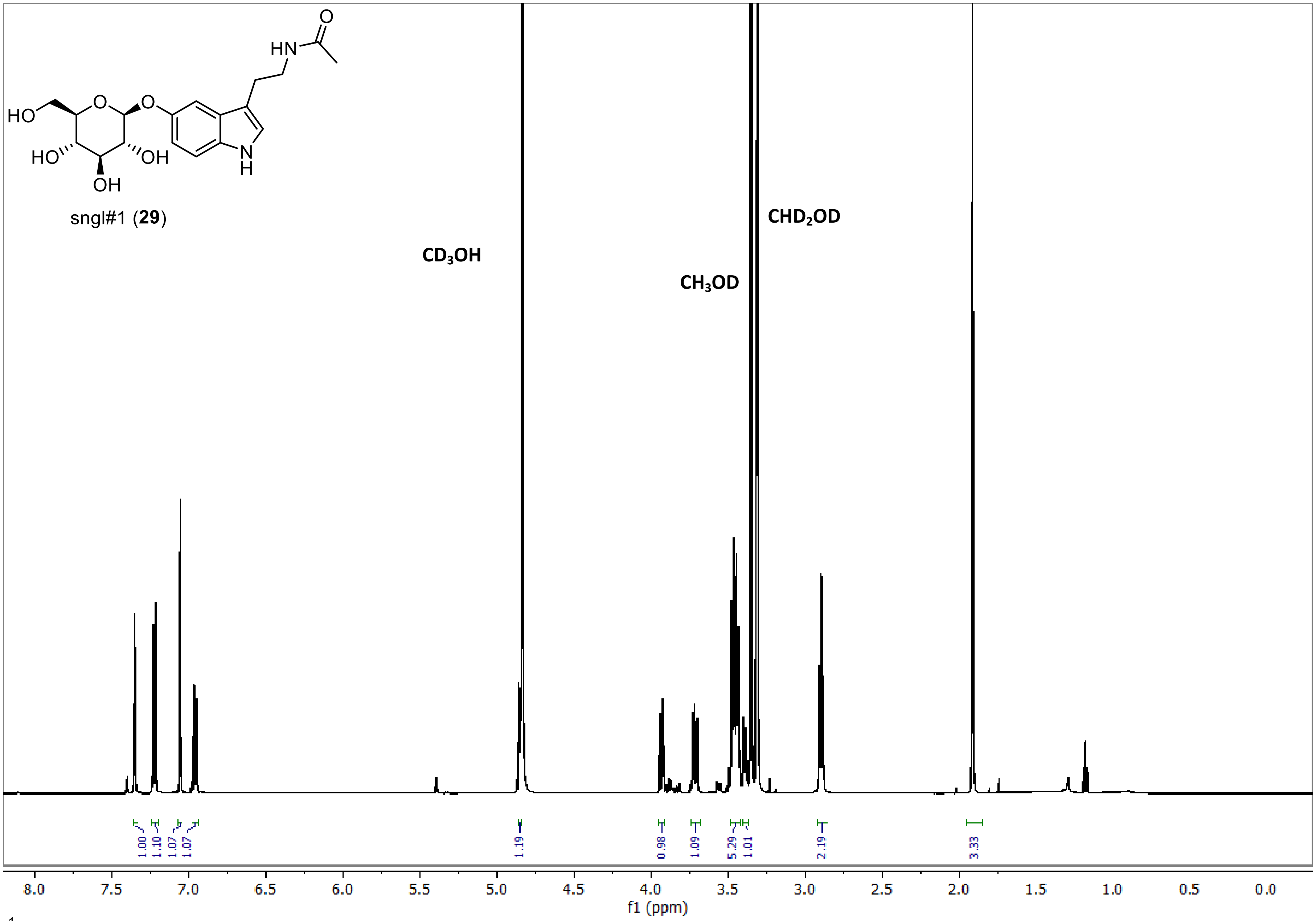

^{1}H NMR spectrum (600 MHz) of sngl#1 (**29**) in methanol-d_4.

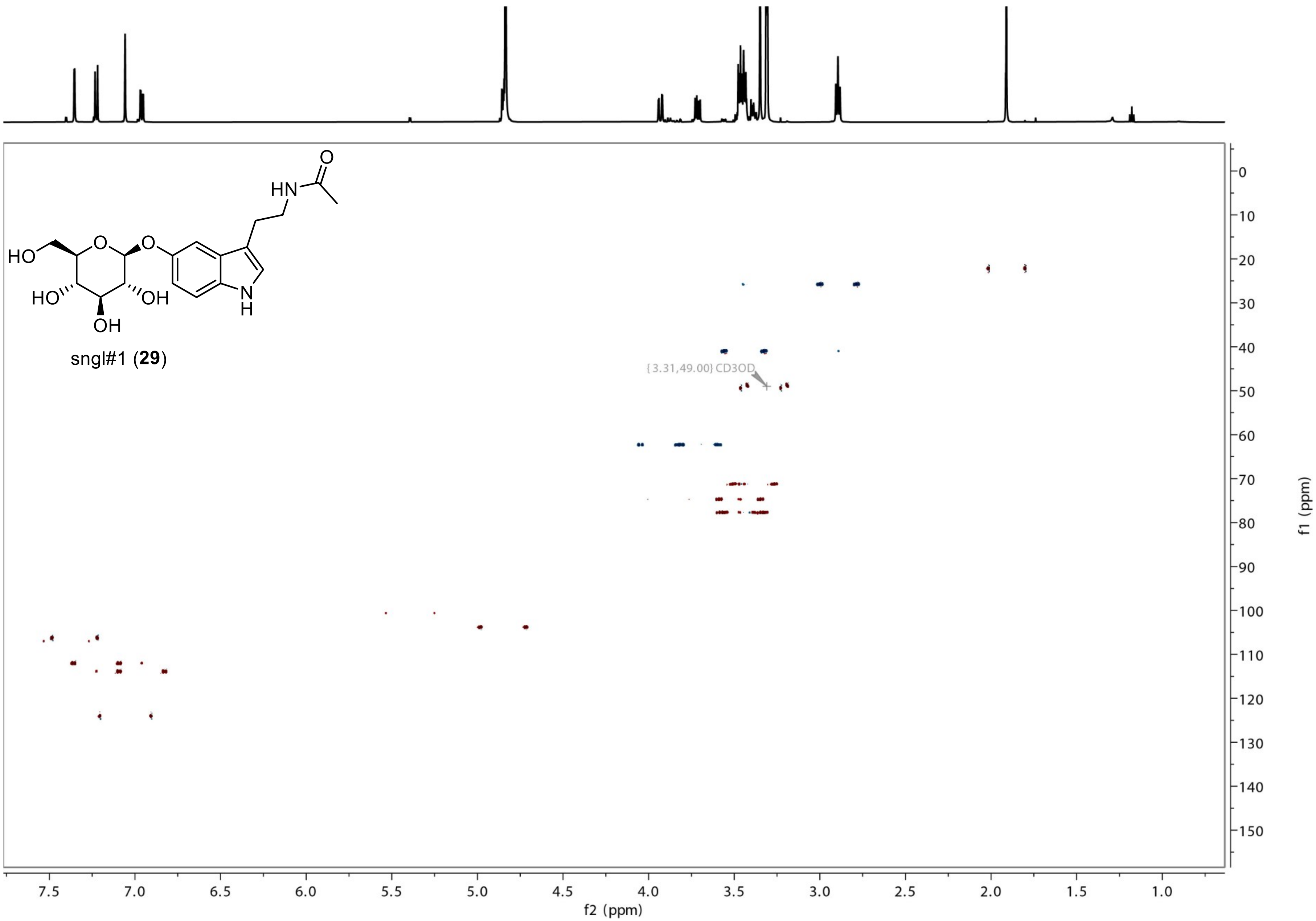

HSQC spectrum (600 MHz) of sngl#1 (**29**) in methanol-d_4.

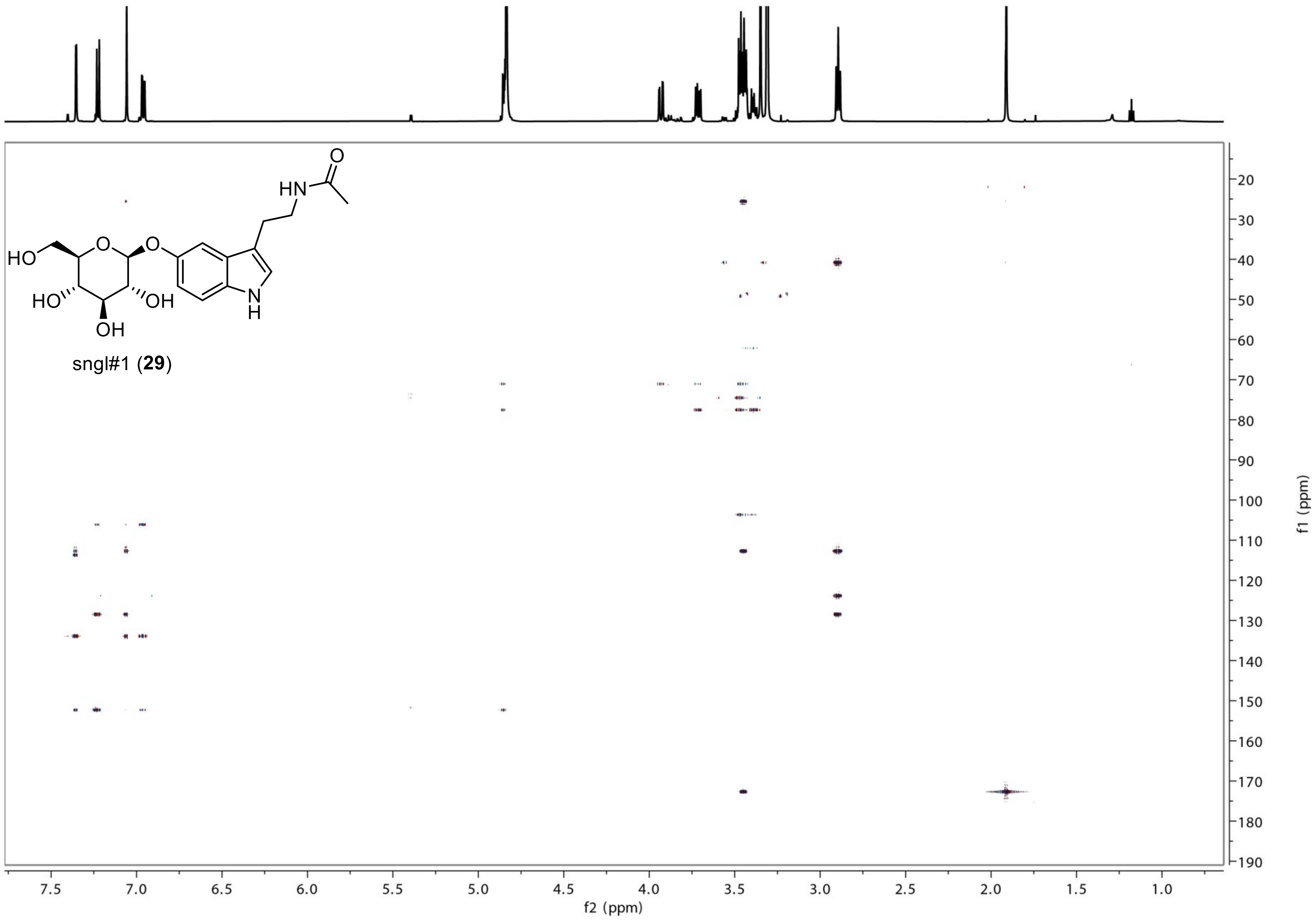

HMBC spectrum (600 MHz) of sngl#1 (**29**) in methanol-d_4.

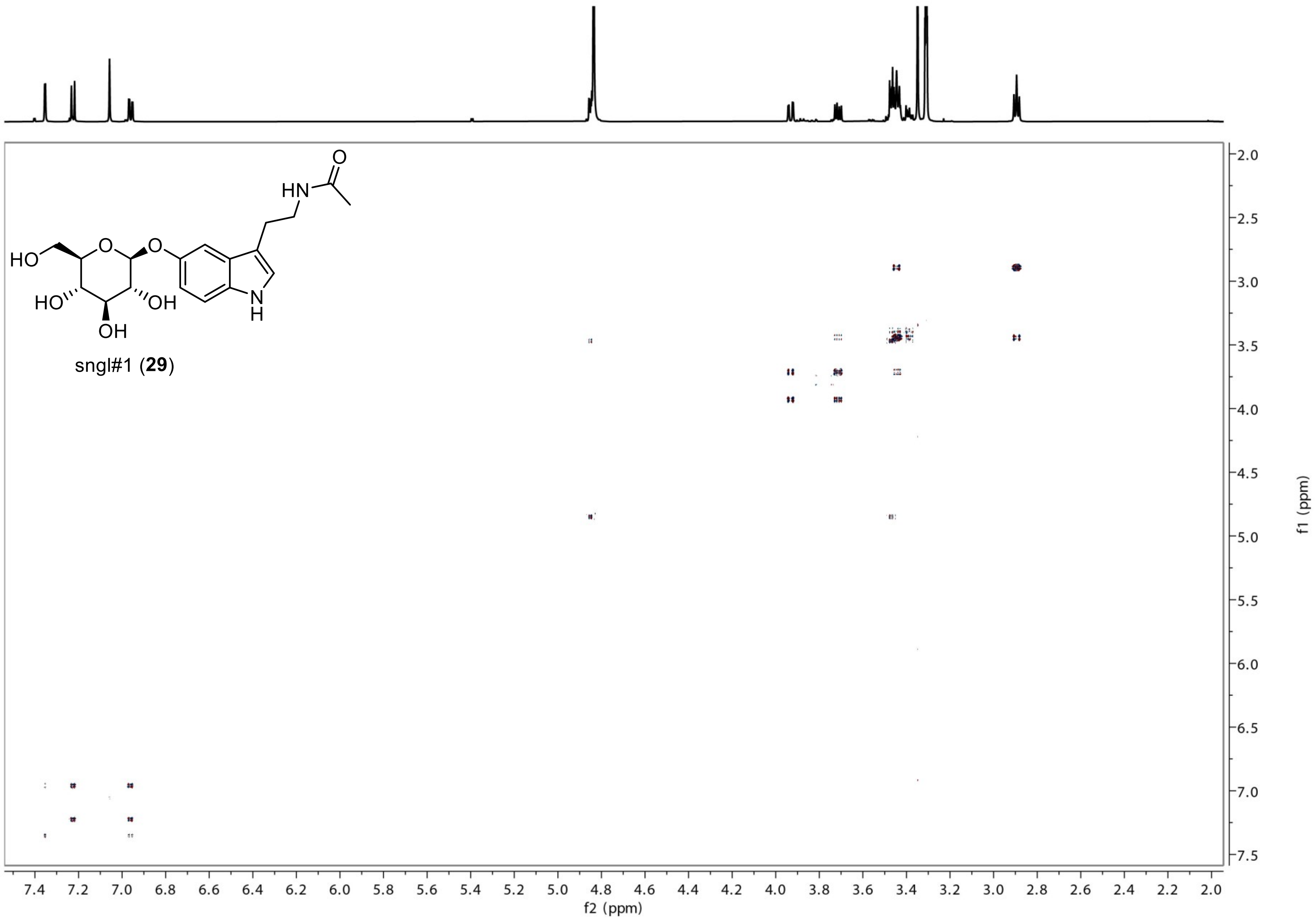

dqfCOSY spectrum (600 MHz) of sngl#1 (**29**) in methanol-d_4.

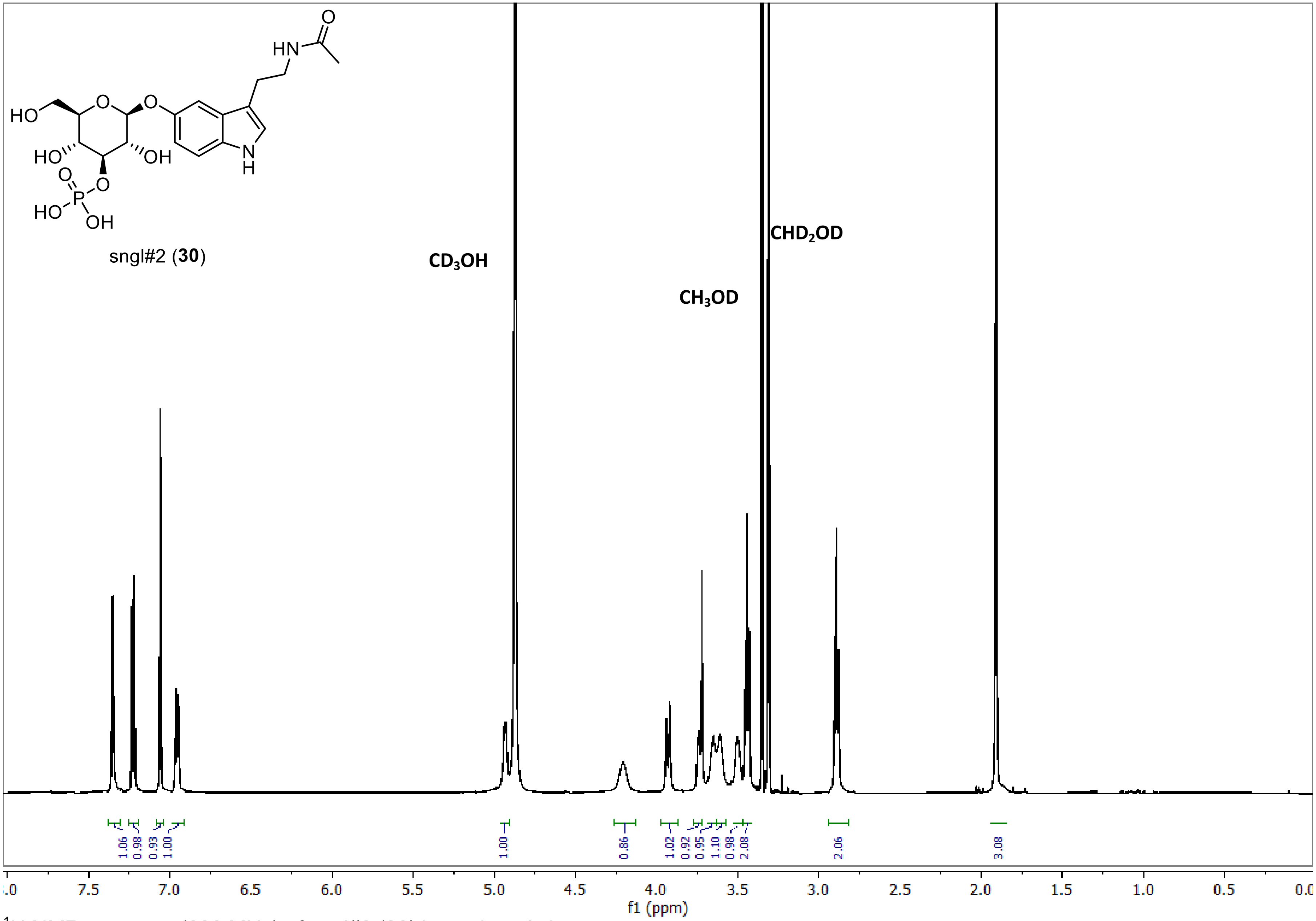

^{1}H NMR spectrum (600 MHz) of sngl#2 (**30**) in methanol-d_4.

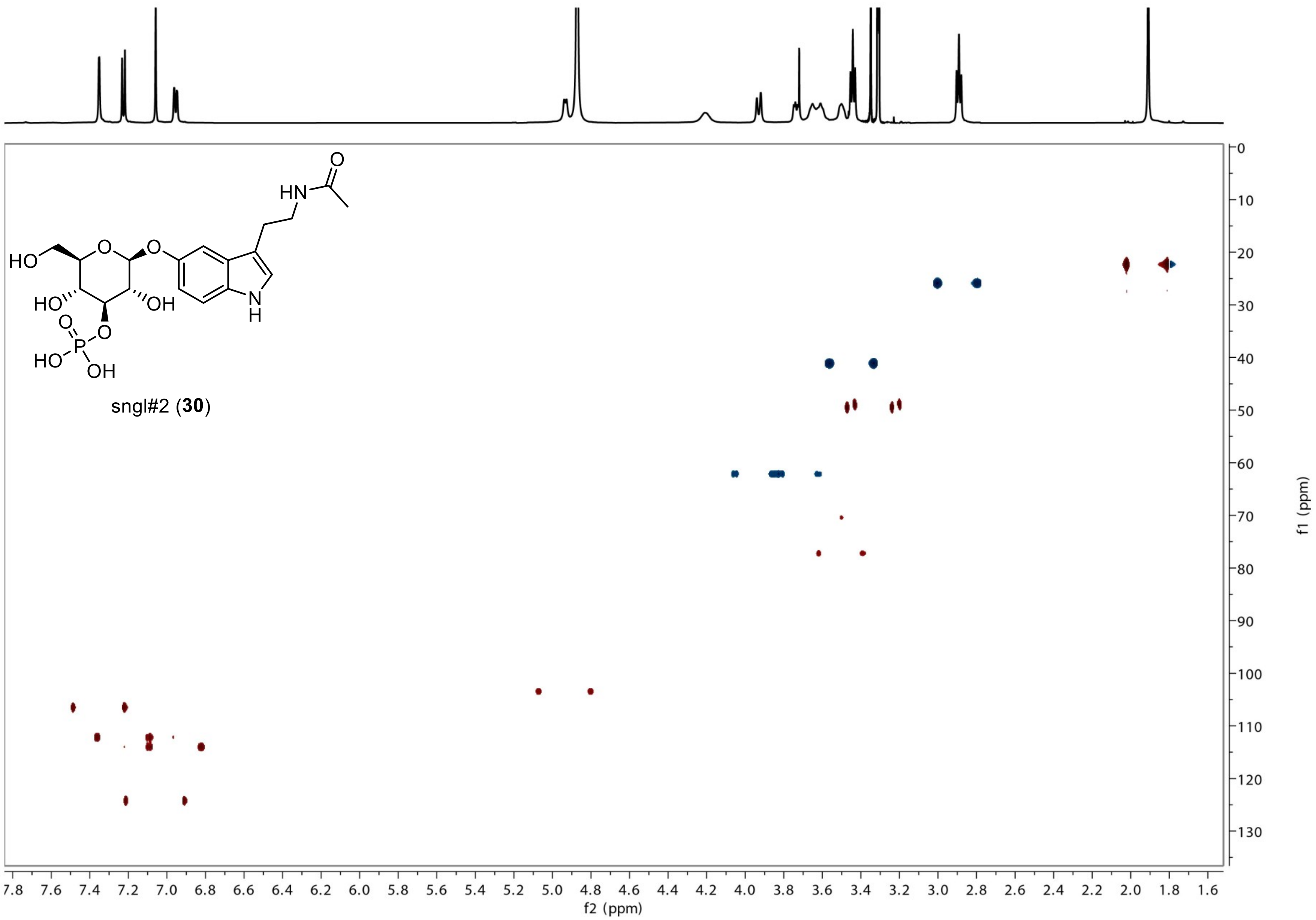

HSQC spectrum (600 MHz) of sngl#2 (**30**) in methanol-d_4.

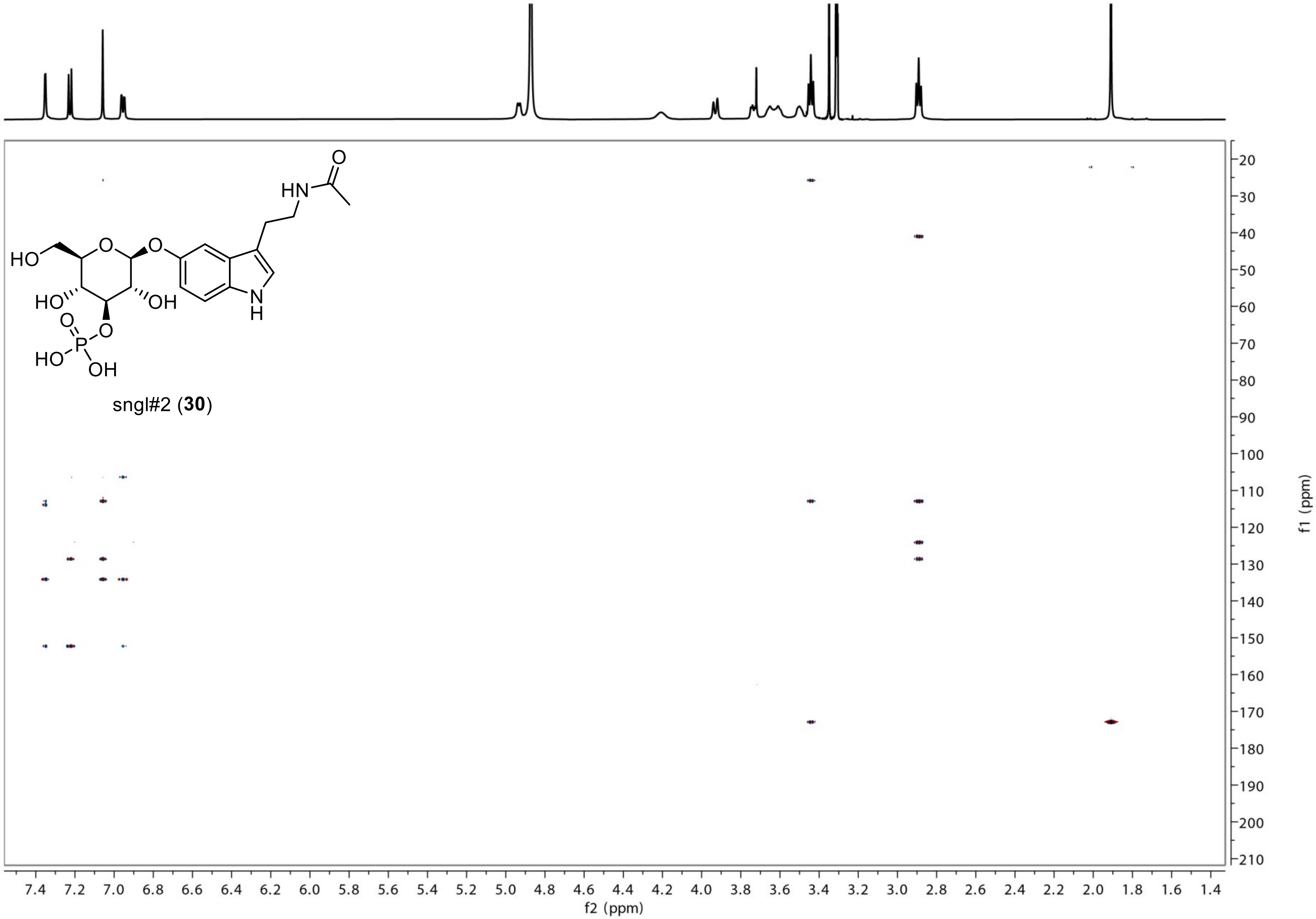

HMBC spectrum (600 MHz) of sngl#2 (**30**) in methanol-d_4.

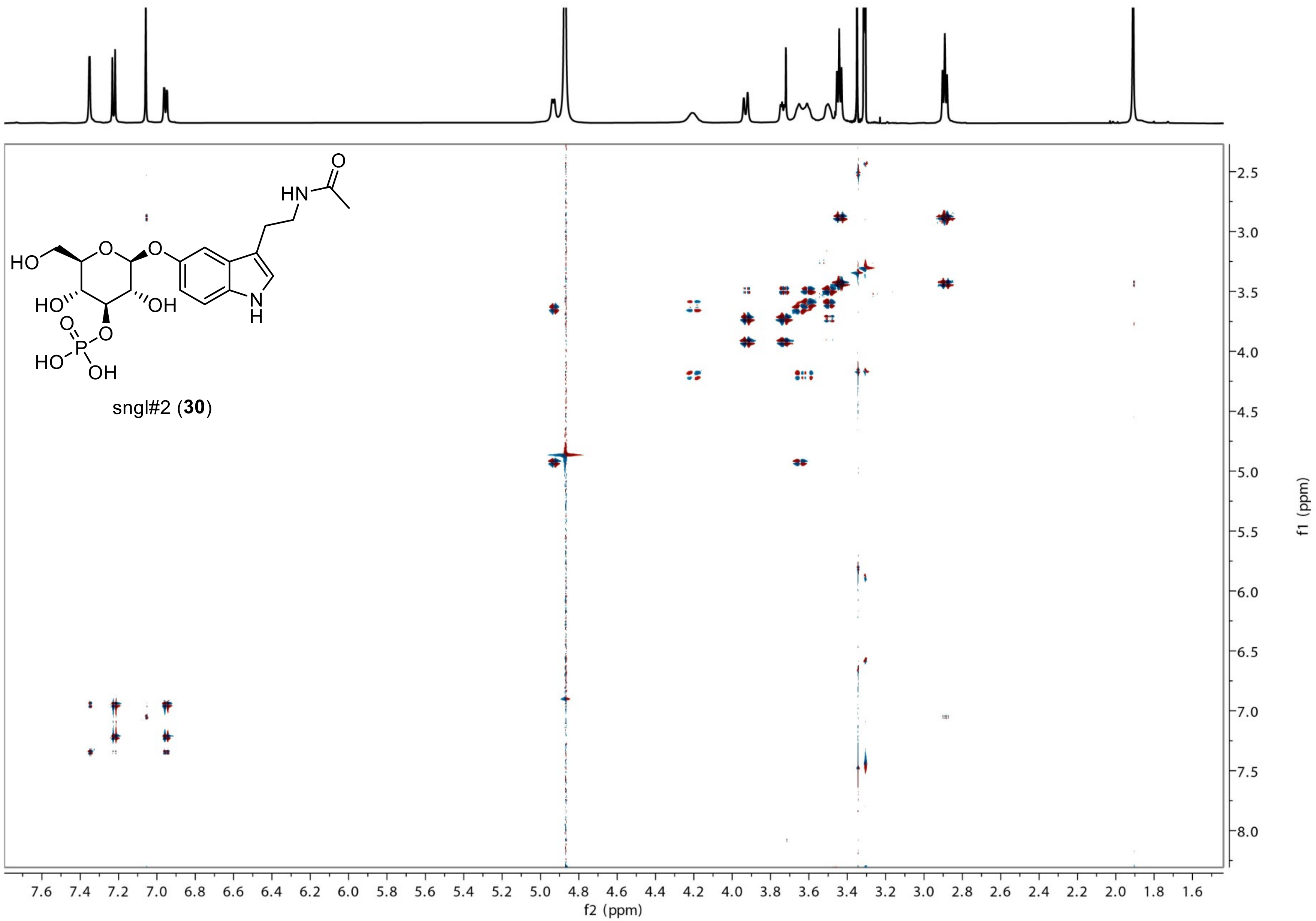

dqfCOSY spectrum (600 MHz) of sngl#2 (**30**) in methanol-d_4.

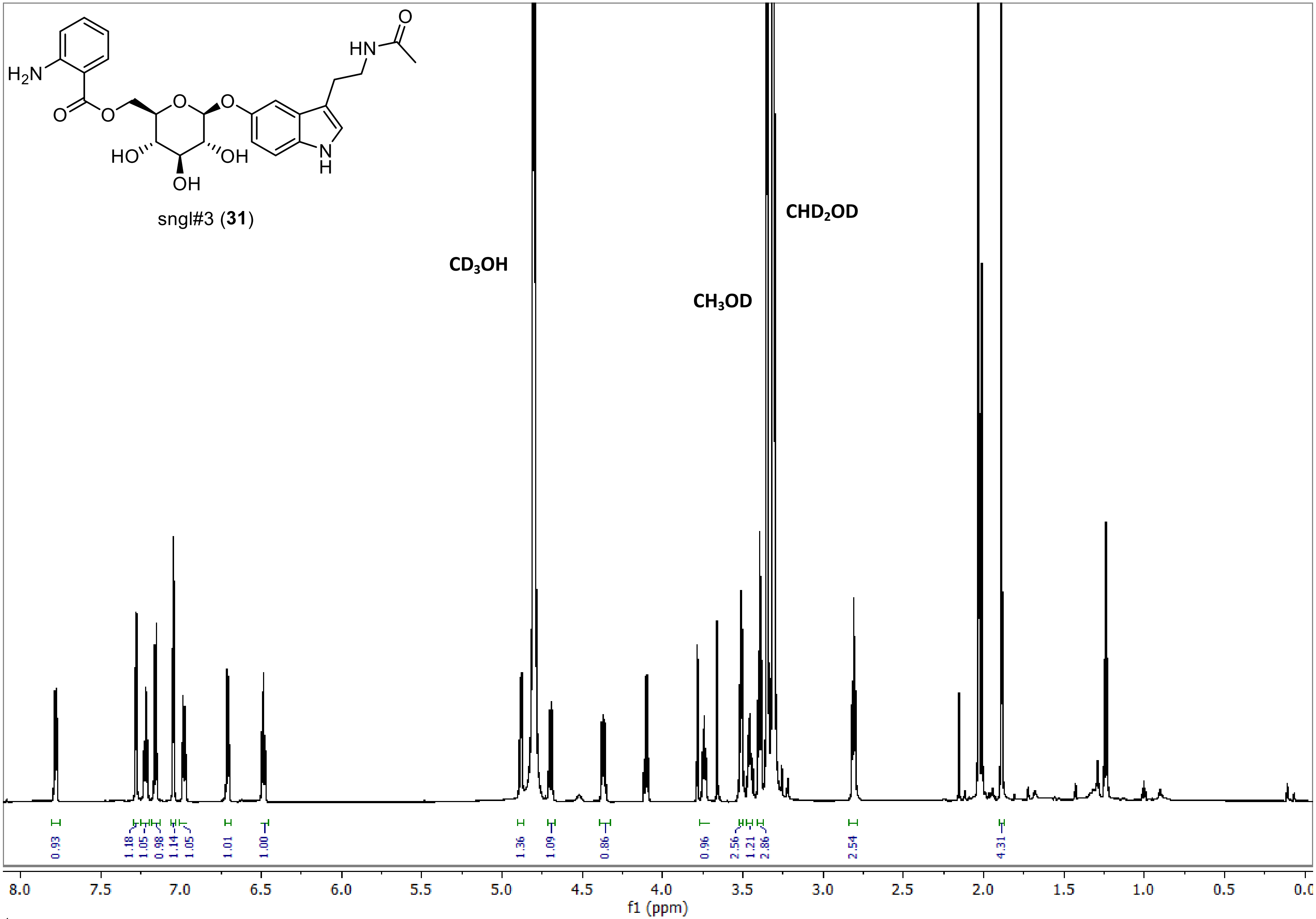

^{1}H NMR spectrum (800 MHz) of sngl#3 (**31**) in methanol-d_4.

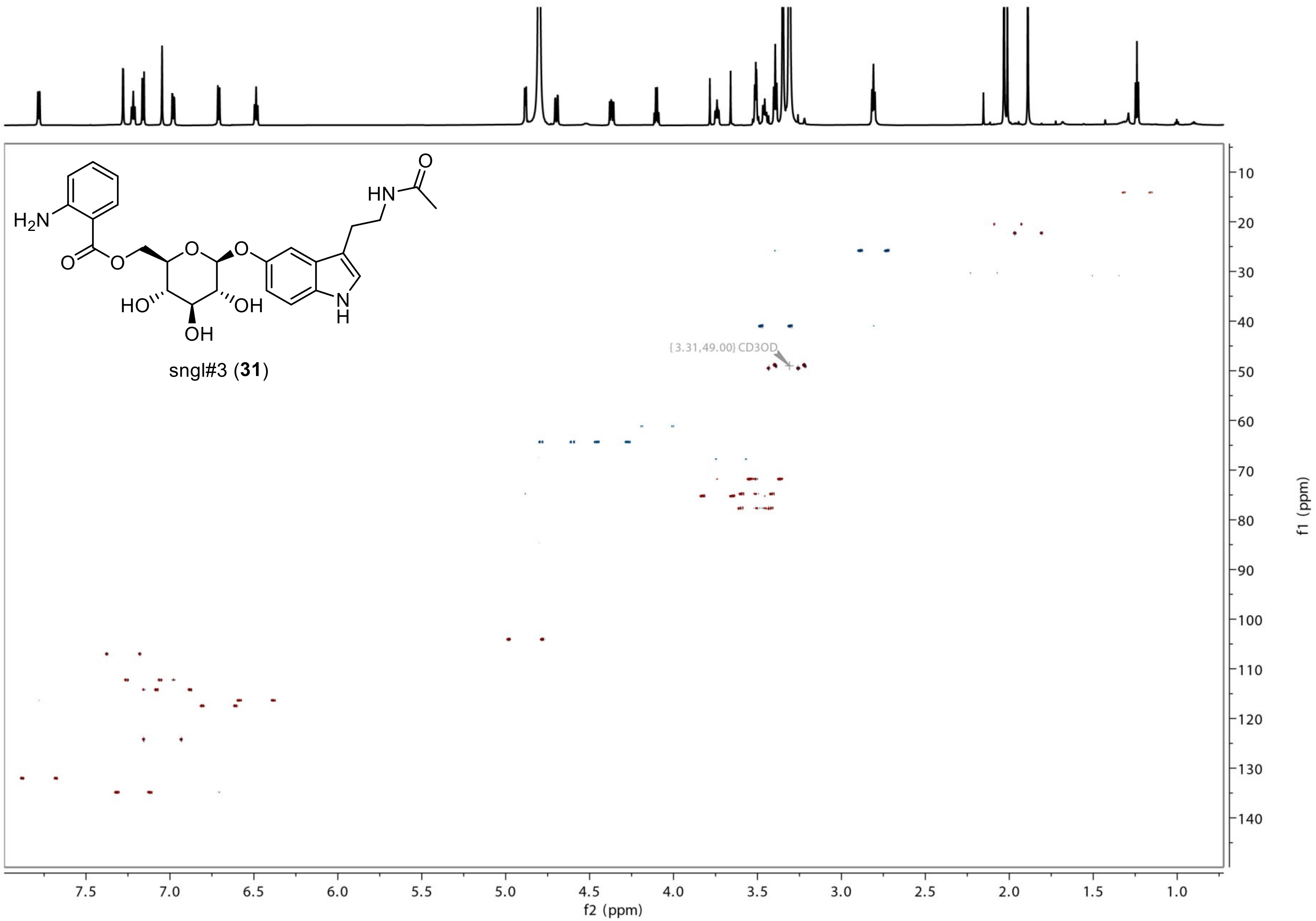

HSQC spectrum (800 MHz) of sngl#3 (**31**) in methanol-d_4.

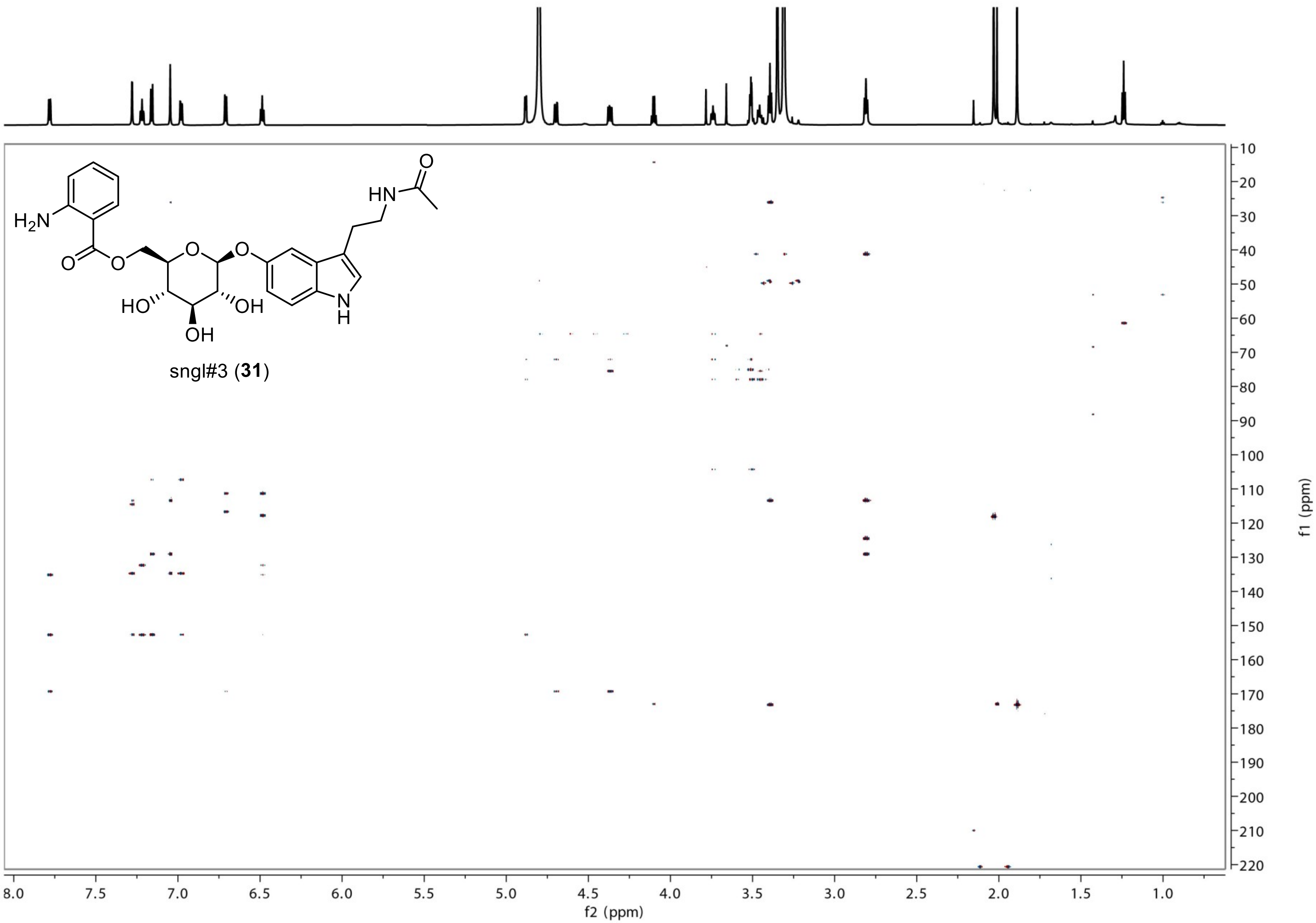

HMBC spectrum (800 MHz) of sngl#3 (**31**) in methanol-d_4.

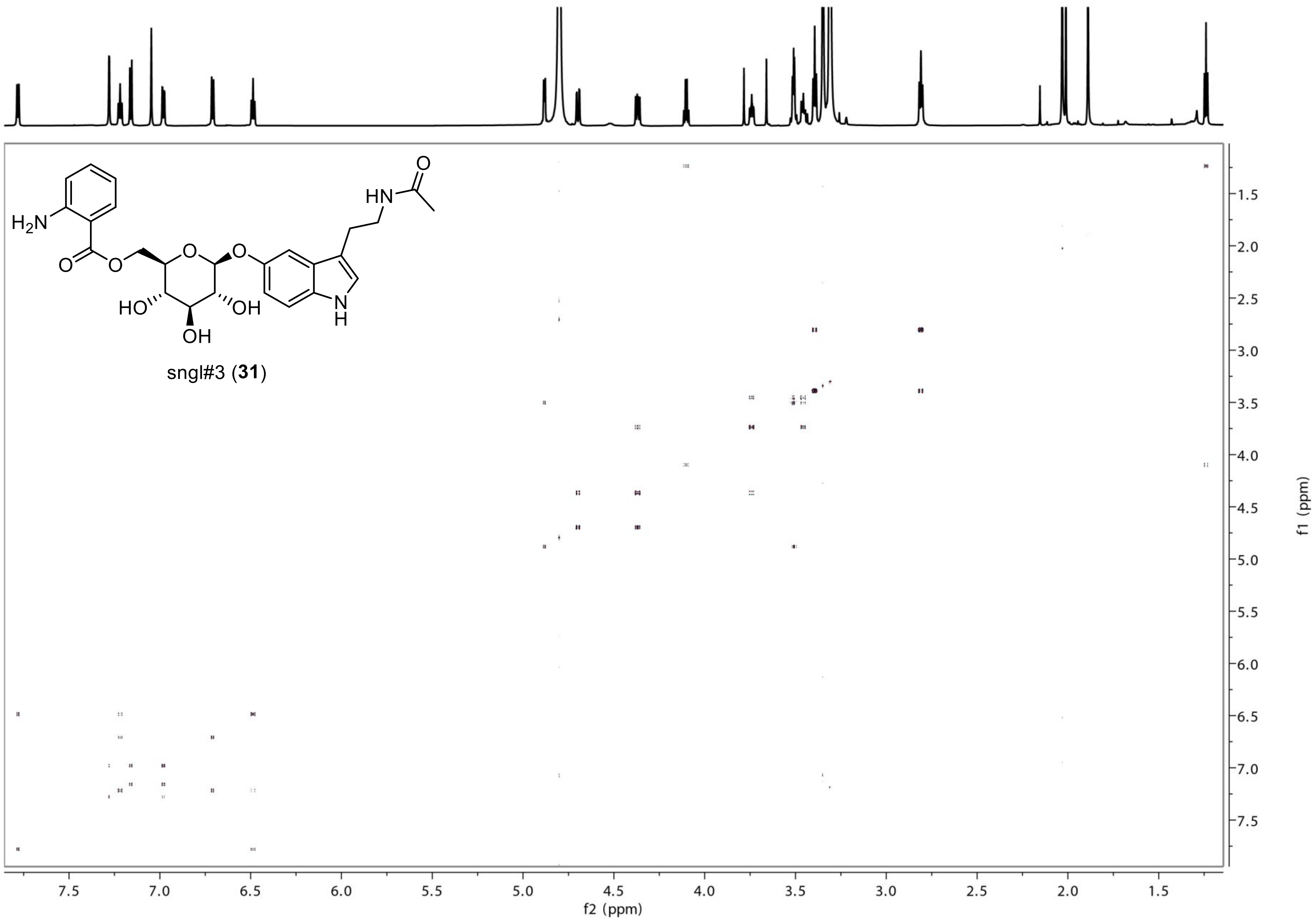

dqfCOSY spectrum (800 MHz) of sngl#3 (**31**) in methanol-*d*4.

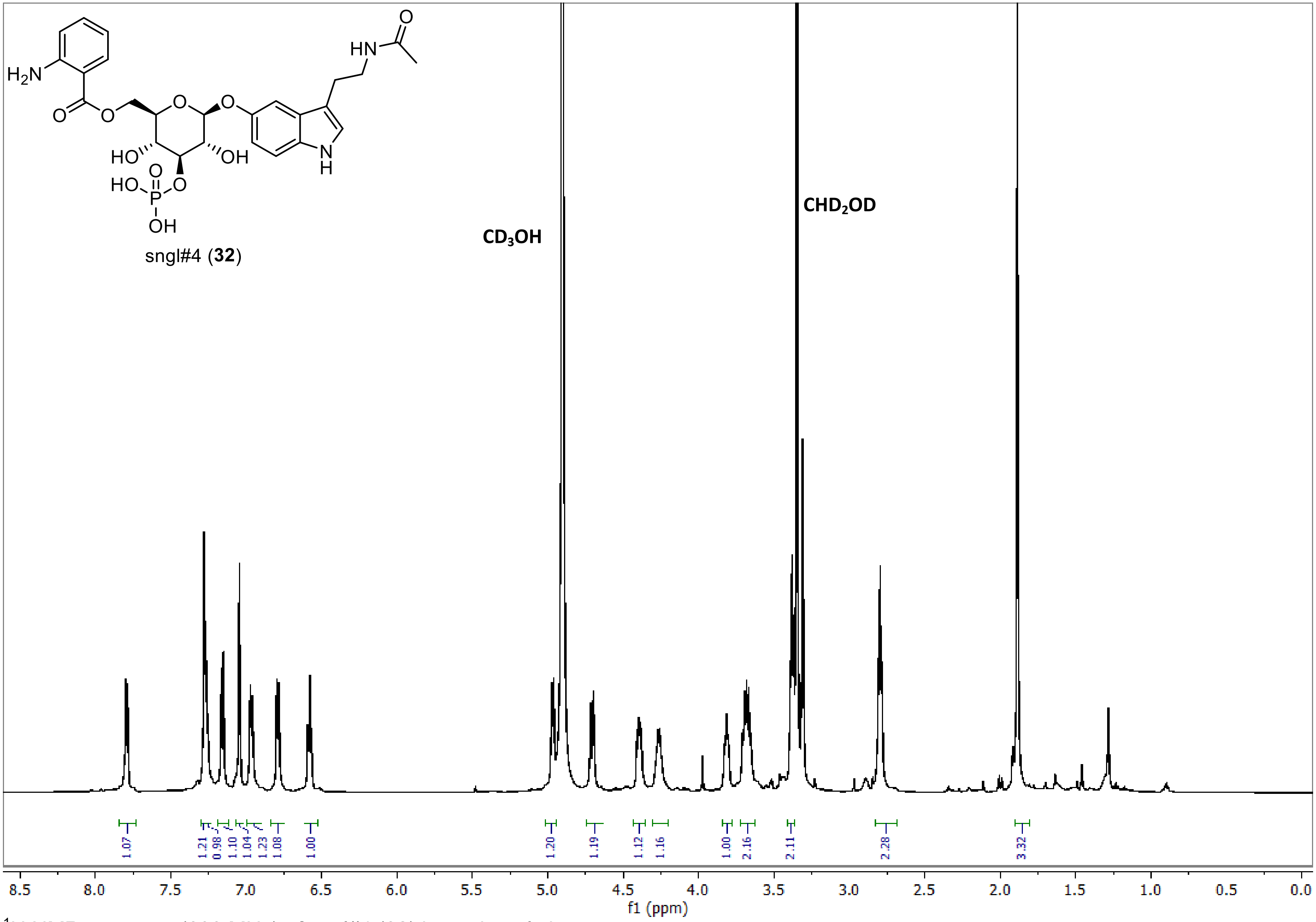

^{1}H NMR spectrum (600 MHz) of sngl#4 (**32**) in methanol-d_4.

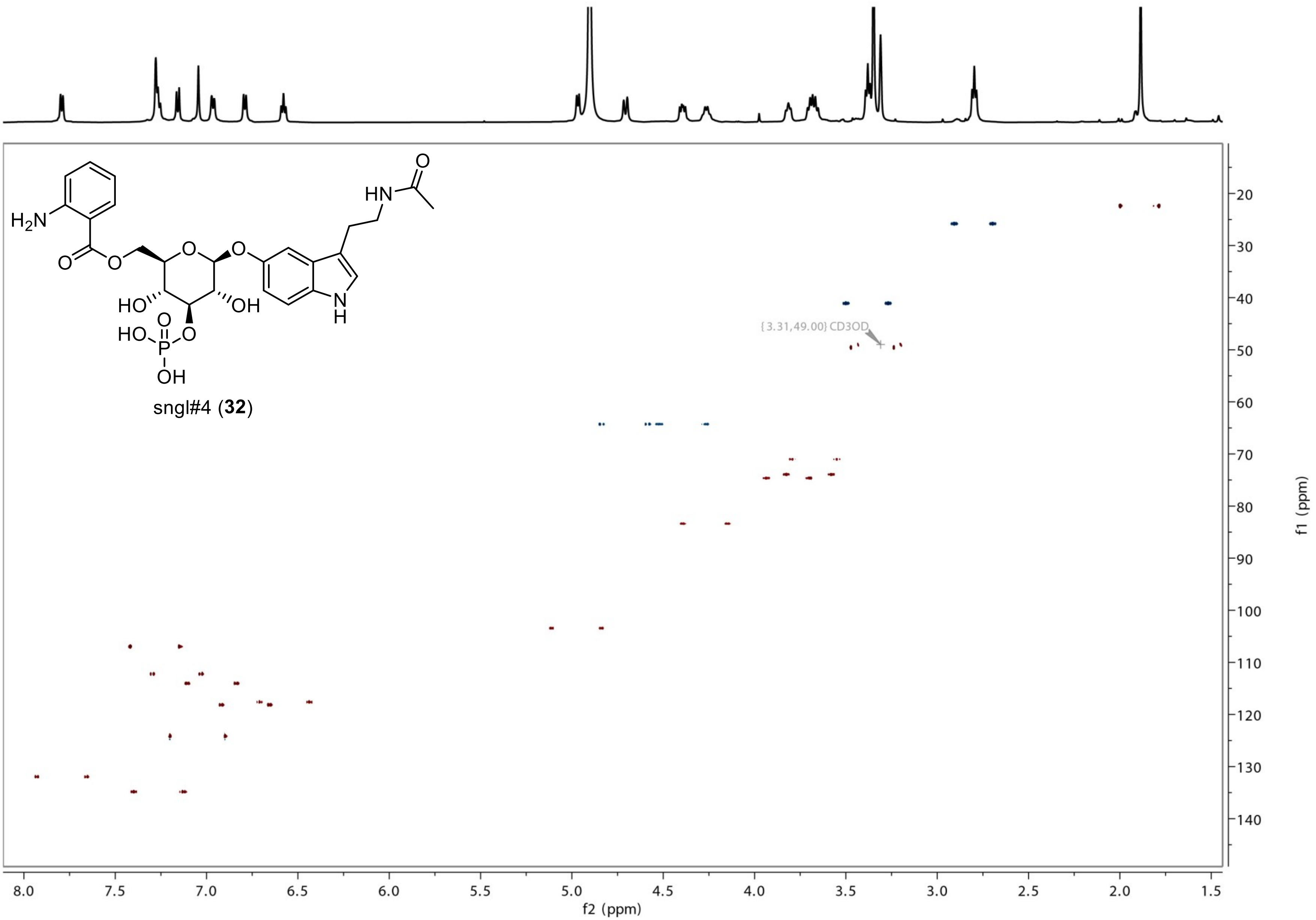

HSQC spectrum (600 MHz) of sngl#4 (**32**) in methanol-d_4.

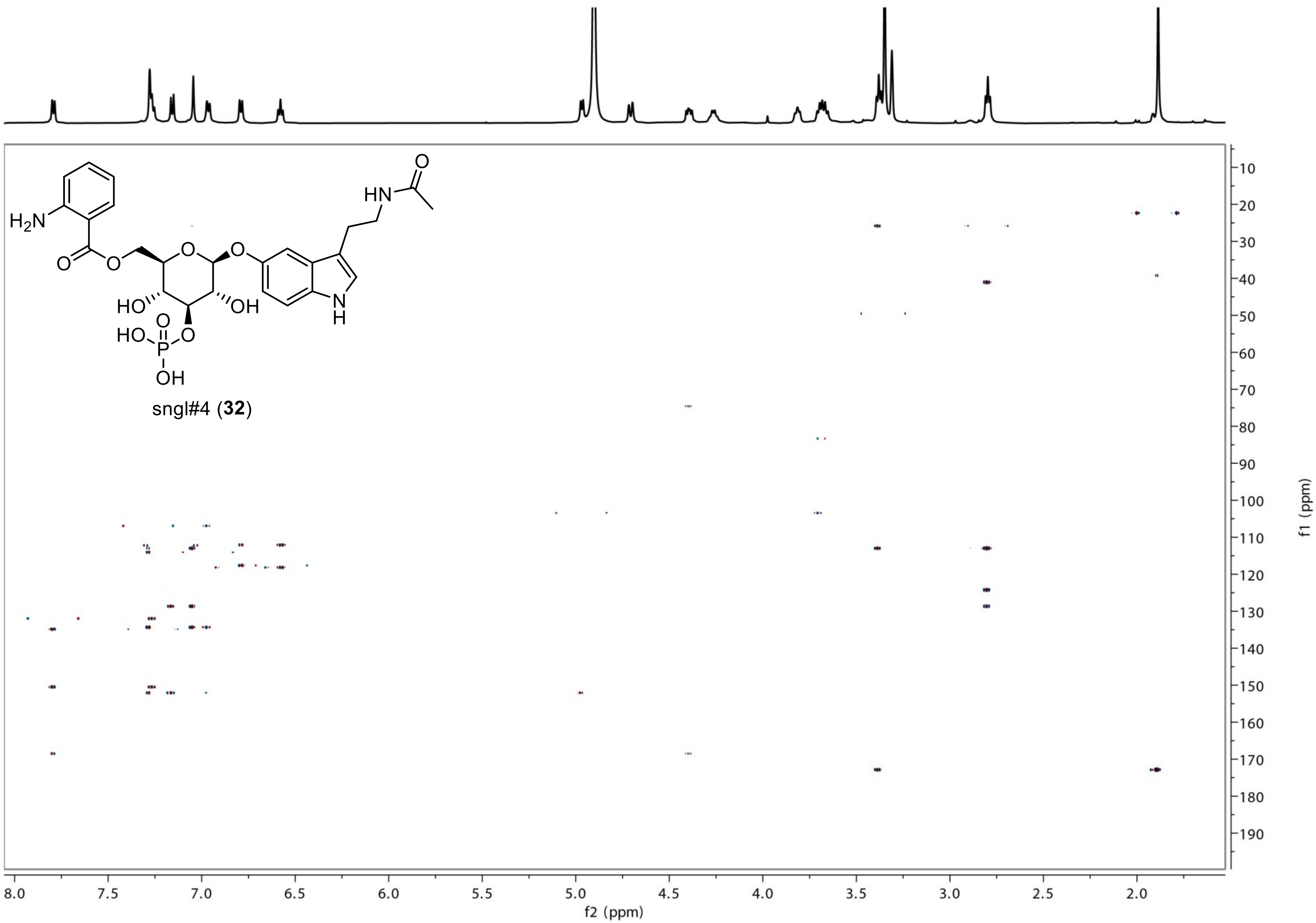

HMBC spectrum (600 MHz) of sngl#4 (**32**) in methanol-d_4.

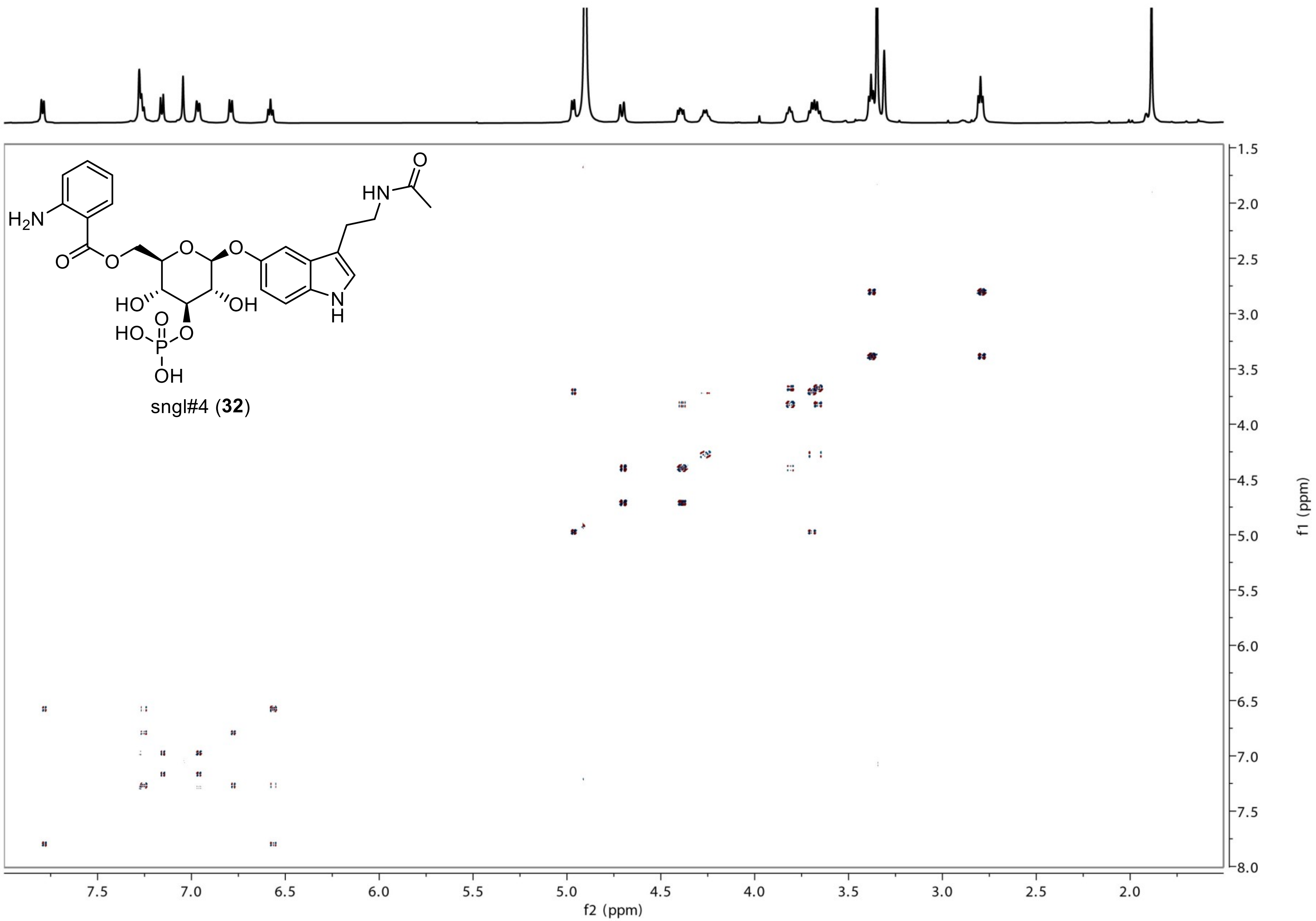

dqfCOSY spectrum (600 MHz) of sngl#4 (**32**) in methanol-d_4.

5. Supplementary References

1. Le, H.H. et al. Modular metabolite assembly in Caenorhabditis elegans depends on carboxylesterases and formation of lysosome-related organelles. *Elife* **9**(2020).
2. Wrobel, C.J.J. et al. Combinatorial Assembly of Modular Glucosides via Carboxylesterases Regulates C. elegans Starvation Survival. *J Am Chem Soc* **143**, 14676-14683 (2021).
3. Baba, T. et al. Construction of Escherichia coli K-12 in-frame, single-gene knockout mutants: the Keio collection. *Mol Syst Biol* **2**, 2006 0008 (2006).
4. Pelletier, G., Zwicker, A., Allen, C.L., Schepartz, A. & Miller, S.J. Aqueous Glycosylation of Unprotected Sucrose Employing Glycosyl Fluorides in the Presence of Calcium Ion and Trimethylamine. *J Am Chem Soc* **138**, 3175-82 (2016).
5. Wadzinski, T.J. et al. Rapid phenolic O-glycosylation of small molecules and complex unprotected peptides in aqueous solvent. *Nature Chemistry* **10**, 644-652 (2018).
